# Latent environment allocation of microbial community data

**DOI:** 10.1371/journal.pcbi.1006143

**Published:** 2018-06-06

**Authors:** Koichi Higashi, Shinya Suzuki, Shin Kurosawa, Hiroshi Mori, Ken Kurokawa

**Affiliations:** 1 Genome Evolution Laboratory, National Institute of Genetics, Mishima, Japan; 2 Department of Biological Information, Tokyo Institute of Technology, Ookayama, Meguro-ku, Tokyo, Japan; University of Trento, ITALY

## Abstract

As data for microbial community structures found in various environments has increased, studies have examined the relationship between environmental labels given to retrieved microbial samples and their community structures. However, because environments continuously change over time and space, mixed states of some environments and its effects on community formation should be considered, instead of evaluating effects of discrete environmental categories. Here we applied a hierarchical Bayesian model to paired datasets containing more than 30,000 samples of microbial community structures and sample description documents. From the training results, we extracted latent environmental topics that associate co-occurring microbes with co-occurring word sets among samples. Topics are the core elements of environmental mixtures and the visualization of topic-based samples clarifies the connections of various environments. Based on the model training results, we developed a web application, LEA (Latent Environment Allocation), which provides the way to evaluate typicality and heterogeneity of microbial communities in newly obtained samples without confining environmental categories to be compared. Because topics link words and microbes, LEA also enables to search samples semantically related to the query out of 30,000 microbiome samples.

## Author summary

In the past decade, microbiomes from various natural and human symbiotic environments have been thoroughly studied. However, our knowledge is limited as to what types of environments affect the structure of a microbial community. In the first place, how can we define “environments”, in particular, the environmental entities that are often continuously varying and difficult to discretely categorize? We assumed that environments could be represented from microbiome data because the structure of microbial communities reflect the state of the environment. We applied a probabilistic topic model to a dataset containing taxonomic composition data and natural language sample descriptions of >30,000 microbiome samples and extracted “latent environments” of the microbial communities, which are core elements of environmental mixtures. Integrating the training results of the model, we developed a web application to explore the microbiome universe and to place new metagenomic data on this universe like a global positioning system. Our tool shows what kinds of the environment naturally exist and are similar to each other on the perspective of the structural patterns of microbiome, and provides the way to evaluate the commonality and the heterogeneity of users’ microbiome samples.

## Introduction

Microbial communities are present worldwide in almost all possible environments. Because the composition (structure) of a microbial community and its surrounding environment are closely related to each other, it is important to understand what kinds of structural patterns are possible and how environmental factors affect community formations. Over the past decade, the structures of tens of thousands of microbial samples derived from various natural environments, including those in symbiosis with humans, have been analyzed. Using these datasets, global patterns of microbial diversity have been characterized that show that community structures constitute distinct clusters among at least certain environments[1–4]. In addition, the community structure of each examined environment has been evaluated using a clearly defined environmental ontology[5,6].

However, the granularities (i.e., the levels of detail) of human-classified environmental categories do not necessarily coincide with structural patterns of microbial communities, and this unavoidable arbitrariness in the granularities of environmental labels may bias the interpretation of results of comparative analysis, such as an enrichment analysis of environments among communities[1]. There are three types of incongruences between environmental labels and community structures. First, there are different subtypes in the microbial community structures associated with certain environments, e.g., enterotypes in the human gut and vaginal community types[7–9]. Second, in contrast to the first case, a nearly identical community structure may be observed across different environmental labels. For example, microbial communities of the surface of the home environment and their inhabitants show highly similar structural patterns[10]. Third, because an environment varies continuously over time and space, it is impossible to define it using a strict segmentation or hierarchical structure. For example, the brackish water of an estuary can have various mixtures of fresh water and seawater, for which the relative proportion continuously shifts[11]. Although an environment is difficult to definitively define owing to uncharacterized factors, these factors can potentially be defined indirectly using microbial community data because microbes respond quickly to environmental changes[12,13], and their community structure reflects the state of the environment[14–16].

Microbial community structures have been analyzed by various data clustering methods. Most of these approaches are based on the evaluation of data densities on high-dimensional feature space in which microbiome samples are distributed. Microbial community structures are complex as they are described by a large number of features (taxa), although not all the features necessarily vary independently. There are groups of taxa that show co-occurrence patterns in samples[17–19]. Herein, we refer to such co-occurrence relationships of microbes as “sub-communities”. Summarizing community structure data as mixing ratios of sub-communities makes it easier to interpret community dynamics according to environmental changes[20]. To extract such partial structures from mixed data, the machine-learning technique denoted a topic model has been extensively studied in recent years since introduction of the Latent Dirichlet Allocation (LDA) model[21]. The LDA model is a probabilistic generative modeling approach, mainly used in natural language processing, for discovering the latent (unobserved) structures of the dataset. The LDA model and its extended models have been used to analyze microbiomes[20,22,23], however, it is often difficult to interpret extracted sub-communities of microbial taxa. Sub-communities have been characterized by evaluating their relationships with occurrences of sample metadata (i.e., data describing information about the samples, such as body sites and gender) after modeling[24], or by explicitly modeling associations between metadata and sub-communities[20]. These methods cannot be applied unless all samples have standardized metadata with a uniform granularity, and thus tend not to be practical for comprehensive analyses of microbial samples from various environments.

All metagenomic data registered in public databases have such metadata, that is, natural language data described by the researchers who registered the samples. The paired dataset of community structures and description documents can be used for modeling the conditional relationship between them. However, natural language descriptions in databases are not always sufficiently described as their content for many samples is often incomplete and has widely variable resolution. For example, in a sample, various information on the host such as race and gender, experimental conditions, the purpose of the research project, etc. are described, but in another sample, it is described only as “human gut metagenome” and is needed to be treated as a sample with missing values. Therefore, for robust modeling, it is necessary to assume stochastic-generating processes not only for community structures but also for documents within the framework of the probabilistic generative model. For such purpose, the Correspondence-LDA (Corr-LDA)[25,26] model can be applied. Corr-LDA is a probabilistic modeling approach that is used to extract correspondence between various types of elements occurring in the same dataset, for example, the correspondence between sub-regions of pictures and their captions[25], between topics of blog documents and their annotation tags[26], or between brain regions and their cognitive functions[27][28].

In this research, we attempted to find relationships between patterns of microbial community structures and patterns of “environments” that the human recognizes and describes. To this end, we applied the Corr-LDA model to pairs of taxonomic compositions and natural language sample descriptions for tens of thousands of sequenced 16S rRNA gene amplicon samples reanalyzed by the unified analysis pipeline. Using this dataset, “topics” extracted by the Corr-LDA model would represent the core elements of environmental mixtures. By integrating training results, we developed an interactive web application denoted LEA (Latent-Environment Allocation), which is freely available at http://leamicrobe.jp. The extracted connections between microbial sub-communities and subsets of English words via topics are applicable to various analyses. LEA enables researchers to do the following: 1) clarify the relationship between environments and patterns of microbial community structures. 2) predict the “latent environments” of new samples from, for example, the ocean, a diseased gut, or another unexpected environment, and quickly compare new samples with tens of thousands of existing samples based on their environmental similarity, which makes it easy to detect dysbiosis of the microbiome in the human gut or contaminants in natural environments. 3) search for samples in the >30,000-sample dataset based on an ecological perspective, without depending on exact word matching of queries and sample descriptions. In this paper, we show the patterns found in the human gut and vaginal communities as an example of separations and connections of extracted “environments”, and show how the LEA global map and a semantic search method on the map make it easy to explore patterns in microbial community structures. In addition, as an example of environmental predictions for newly acquired microbiome samples, we show the LEA mapping results for the datasets of the human gut microbiome and the microbiome derived from various natural environments.

## Results

### Overview of the LEA global map

We collected sequenced 16S rRNA gene amplicon samples from the MicrobeDB.jp database and performed a phylogenetic analysis using VITCOMIC2[29], which is the metagenomic analysis pipeline improved from VITCOMIC[30], on all samples. This resulted in a dataset containing 30,718 samples with genus level information on their taxonomic composition linked to a document containing sample description information. For this dataset, model inference runs were performed by Corr-LDA with a varying number of topics. The perplexity, which is the performance evaluation index of the model (smaller values indicate a better performance), was sufficiently small for the model with 80 topics (S1 Fig). In the following sections, we discuss the results for the model inferred with 80 topics.

The inferred word subsets and microbial sub-communities for each topic are shown in S2 and S3 Figs. Each topic has a unique subset of words and a microbial sub-community. The structure of a microbial community sample that has a large proportion of a certain topic is likely to contain microbes in the sub-community of that topic, and the description of the sample is likely to contain words in the word subset of that topic. For most topics, the word subset associated with the topic represents a single natural environment or a symbiotic environment with humans.

**Fig 1:**
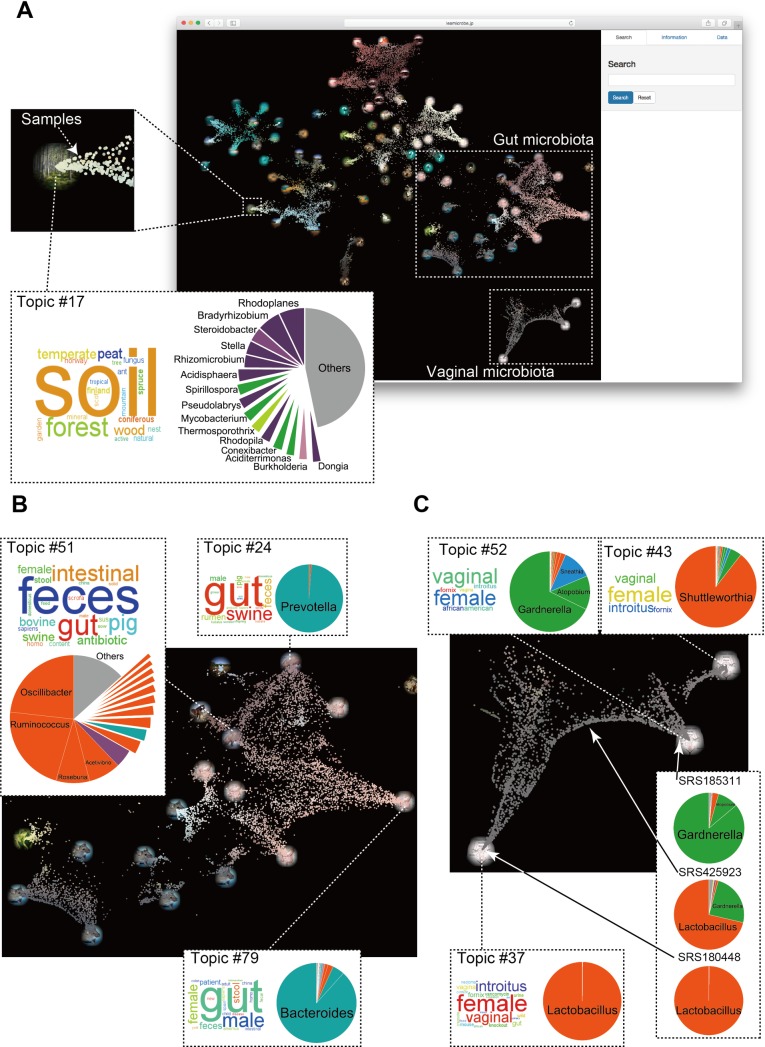
The LEA global map. (A) Scatter plot of the 30,718 samples from various environments. Coordinates were calculated by t-SNE based on the similarity of topic compositions among samples. Each dot represents a sample. Pictures correspond to topics and are mapped to coordinates where the mixing percentage of the topic is 100%. Each topic has its own word subset and microbial sub-community. As an example, for topic #17, a word subset related to “soil” is displayed by the word cloud (the size of each word is scaled by its probability of occurrence), and the microbial sub-community, which contains mainly soil bacteria, is displayed as a pie chart. (B) Enlarged view of the region related to the gut in the global map. (C) Enlarged view of the region related to the vagina in the global map. The colors of the genera in the pie charts are determined based on their related phyla (see S2 and S3 Figs, which shows the word clouds and pie charts for all topics).

Based on the topic composition of each sample, the similarities among the samples were visualized using parametric t-SNE[31]. In Fig 1, the dots represent the 30,718 samples and the pictures represent 80 topics. A sample that is mapped near a picture indicates that the sample has a large proportion of that topic. On the map in Fig 1C, the sample (SRS425923), which is located approximately midway between the two pictures, has the two topics (topic #37 and #52) mixed in similar amounts.

The 80 topics can be regarded as latent environments that affect the formation of microbial community structures. Topics form several clusters with dense connections formed by many samples but with sparse or no connections between clusters. Topics can be roughly divided into gut, skin, vagina, oral cavity, ocean, and soil. In addition, there are several isolated topics, which include a coral reef, a mosquito, phyllosphere, etc.

### Community types found in various environments

The gut microbiome in healthy adult humans are reported to consist of three[7] or four[24] community types. However, whether truly discrete clusters exist as individual gut microbial communities remains in doubt[32]. For the gut community types (enterotypes), the key genera characterizing each community type have been identified—*Bacteroides*, *Prevotella*, and *Ruminococcus*[7]—although the abundance of key genera varies between samples instead of being discretely clustered[32,33]. Therefore, unlike discrete clusters, e.g., blood types, the compositions of microbial communities are continuously shifting, perhaps as a result of environmental factors. Such continuous variation of the structure of a microbial community can be discerned by our method. 22 topics are related to the gut according to the word subsets associated with each of the topics (S8 Fig), including those with a large proportion of *Bacteroides*, *Ruminococcus*, and *Prevotella* (topics #79, #51, and #24. Fig 1B). However, most of the samples do not reside near a single topic but instead occupy an intermediate position between multiple topics, meaning that the samples are a mixture of several topics. Because many samples with intermediate properties owing to multiple topics exist, there is variability across a limited area of the gut microbiome. *Bacteroides* is often found in the guts of people who eat diets rich in protein and fat, and *Prevotella* is often found in the guts of vegetarians[12,34]. In fact, words denoting herbivores, e.g., “pig”, “swine”, “horse”, “bovine”, and/or “rumen”, are frequently found in the *Prevotella*-rich topic and in topics that are peripherally connected to the *Prevotella*-rich topic (Fig 1B).

Regarding the vaginal flora, three related topics were found (Fig 1C): the *Lactobacillus*-rich topic (#37); the topic including *Gardnerella*, *Sneathia*, and *Atopobium* (#52); and the *Shuttleworthia*-rich topic (#43). Vaginal community types (Community State Type; CST) have previously been examined in detail with five CSTs recognized to date: four (CSTs I, II, III, and V) in which *Lactobacillus* species dominates and one (CST IV) with various obligate or facultative anaerobes and very few *Lactobacillus*[9,35]. The two topics detected in our model are consistent with the above results (Fig 1C). Because we used community structure data found at the genus level, we cannot distinguish between CSTs I, II, III, and V, so these communities were identified as a single topic dominated by *Lactobacillus*. For the second topic corresponding to CST IV in which *Gardnerella* and *Atopobium* dominate, the associated samples likely were obtained from African-American women (as estimated by the word subset of topic #52). Interestingly, samples in which the *Lactobacillus*-rich and *Gardnerella*-rich topics are mixed in various proportions are frequently found, as indicated by the many dots that connect these two pictures (Fig 1C). Vaginal bacterial communities are known to be stable throughout pregnancy and to be relatively stable throughout the menstrual cycle although changes in the *Lactobacillus* spp. populations have been observed[36,37]. Therefore, an environmental gradient of unidentified factors may exist in the vagina, which would cause a community structure to exist as an intermediate state between two topics. Another topic related to the vaginal environment is a topic dominated by *Shuttleworthia*. The presence of *Shuttleworthia* may be related to bacterial vaginosis[38] or to squamous intraepithelial cervical lesions[39], but its ecology is not well understood. Interestingly, the continuous transition of samples to the *Shuttleworthia*-rich topic links only with the *Gardnerella*-rich topic (Fig 1C).

### Prediction of topic compositions for new samples

LEA can predict latent environmental topics of newly acquired samples using the Bayesian prediction method with the identified 80 topics (see Methods). By examining the word subsets associated with the mixed topics, the environment in which new samples are found can be estimated. In addition, by training the dimension-reduction function of t-SNE in our system using a neural network procedure, it is possible to arrange the locations of new samples on the global map (Fig 1A) according to their topic compositions without changing the coordinates of previously mapped samples. The topic prediction of a new sample and its placement on the map are implemented by the LEA web application. By uploading the taxonomic assignment file of VITCOMIC2, the placement of the sample on the map can be viewed in a few seconds.

As examples of LEA mapping results, we have analyzed the dataset of a time-series human gut microbiome analysis[40], which consists of fecal samples obtained every day from two male subjects from the US (subjects A and B). The results are shown in S4 Fig. The LEA visualization reproduces the results of David *et al*.[40], such as the stability of the gut microbiome of subject A over the course of the experiment with the exception of his time in Southeast Asia, and the change in the gut microbiome of subject B caused by an infection. Such results can be easily obtained using the LEA web application.

In a meta-analysis of a large-scale dataset, the existence of systematic bias due to the difference in methods across studies often becomes a problem. We tried to address this problem by processing all samples with a unified information analysis pipeline, but there is a possibility that a further upstream, sample preparation protocol could be a confounding factor. In particular, a bias due to differences in DNA extraction methods often becomes a problem[41]. To assess the impact of different DNA extraction methods of the human gut microbiome analysis on the locations on LEA global map, we conducted LEA environment predictions for the Microbiome Quality Control (MBQC) dataset[42]. This dataset contains 16S amplicon sequencing data from human stool samples, chemostats, and artificial microbial communities. For the same biological sample, there are multiple sequencing data analyzed with different wet laboratories or different DNA extraction methods. The results are shown in S10 Fig. First, most of the samples derived from human feces in the MBQC dataset were properly mapped to the “gut” area of the LEA global map (S10A Fig). As a whole, there was no tendency for samples processed with a specific DNA extraction kit to be mapped only to a specific topic. Therefore, separation of topics on LEA is not necessarily influenced by differences in experimental protocols. However, considering samples that have a same biological origin, some samples were mapped to the nearly same position on the map, and the others were mapped on the location biased by the DNA extraction kits (S10B–S10S Fig). The direction of biases probably differs depending on the position of the true taxonomy composition. Therefore, topics may partially contain systematic bias due to differences in studies, and caution is necessary for interpretation.

**Fig 2:**
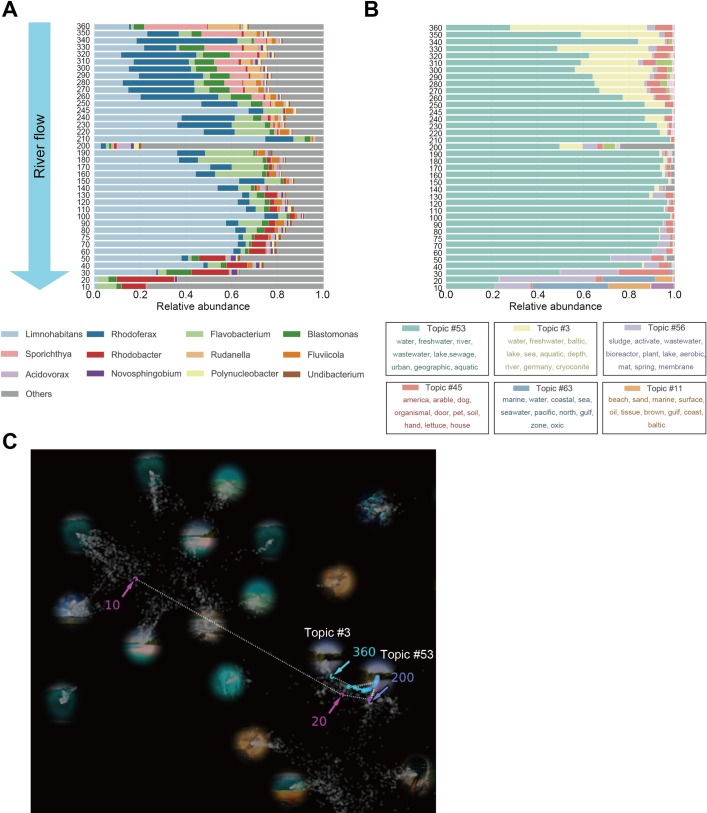
The Tamagawa river microbiomes. Samples taken from various points along the Tamagawa river, which were not used to train the model or in the construction of the initial global map, were mapped onto the global map based on the predictive results of their topic compositions. (A) The genus level taxonomic composition of samples from the surface water of 38 sites along the Tamagawa river. Site 360 is the most upstream site, and site 10 is at the estuary of the river. (B) Topic composition of the Tamagawa river microbiome. Each color represents a topic. The top 10 words with the greatest generation probability for each topic are also displayed. (C) Mapping of the Tamagawa river topic compositions onto the global map. Dark gray dots are samples that were part of the training set. Colored dots with a gradient from light blue to purple represent the sites along the Tamagawa river from which the samples were obtained, e.g., the site 360 sample (the upper most part of the river) is colored light blue, and the site 10 sample from the estuary is colored purple. The trajectory from the upstream to the downstream is indicated by a dotted line.

As an example of LEA involving a natural environment, Fig 2 shows the topic predictions for 38 samples of microbial communities obtained over a short period of time and at a high density from various points along the Tamagawa river in Japan. The upstream region of the river begins in a deep mountainous region; the middle region flows through a densely-populated zone where there is water from sewage treatment plants and from tributaries that joins the river; and the downstream-most region flows into Tokyo Bay. On May 26 and 27, 2015, we sampled the surface water of the river at 38 points (S5 Fig, S1 Table) and identified the microbes contained in the samples by VITCOMIC2 after sequencing of their PCR-amplified 16S rRNA genes. The genus level taxonomic compositions are shown in Fig 2A. *Limnohabitans* is the major genus found in the samples from the river. The microbial community structure of the river continuously shifted as it flowed to the estuary, but sample 200 had a greatly different structure. Sample 200 was obtained just under the sewage treatment facility, and its composition probably reflects the microbiome of the treated water. The community structures of samples 10, 20, and 30, which were obtained from brackish water in the estuary, also differed greatly from those collected elsewhere along the river.

The topic predictions for the river samples are shown in Fig 2B. After performing LEA, two topics related to “river” were found: one was topic #3, which frequently occurs together with words such as “Baltic sea,” “lake,” and “river,” and the second was topic #53, which is associated with the words, “river,” “wastewater,” and “urban.” The aforementioned words belong to the dominant topics in Fig 2B. Topic #3 is primarily associated with the upstream region of the river and topic #53 with all areas of the river. The relative proportions of these two topics gradually change as the river flows downstream. Given the word subsets associated with the two topics, topic #3 represents freshwater ecosystems, such as lakes and rivers, and topic #53 represents river areas adjacent to cities. Samples 10 and 20 (from the estuary) are largely associated with topics #11 and #63, which represent the ocean, and, along with sample 30, are associated with topic #56, which represents activated sludge. For the Tamagawa river, about half its water that flows into the estuary is treated water[43]. Thus, our results suggest that the mixing of the river water with seawater greatly changes the community structure and that the river’s ecosystem is greatly affected by it interaction with the urban area.

Topic #45 is associated with the upstream region of the Tamagawa river (sample 360 to 250); the words associated with this topic include “pet” and an indoor environment (Fig 2B). Many of the 16S rRNA sequences associated with topic #45 belong to *Blastomonas*, a genus associated with domestic wastewater, which is found in tap water, faucets, and shower hoses and is resistant to disinfection[44–46]. Advanced sewage treatment facilities are not found in this region of the Tamagawa river, suggesting that untreated household wastewater is being dumped into the river.

Most of the Tamagawa river samples were mapped near “freshwater” topics #53 and #3 on the global map (Fig 2C), although the topics of sample 200, taken near the sewage treatment plant, and samples 10, 20, and 30, taken from the estuary, diverged, to some extent, from the freshwater topic. Specifically, sample 10 were mapped within “ocean” topics. In this way, the LEA web application can place a new sample appropriately on the global map of existing samples and enables visual and intuitive operation to evaluate its characteristics, e.g., deviations from the expected environments.

For further testing of LEA using external dataset, we conducted environmental predictions of microbiome data derived from a highly diverse environment produced by the Earth Microbiome Project (EMP) [47]. One of the good points about this dataset is that every sample is given an environmental label based on a controlled vocabulary, the EMP Ontology (EMPO). EMPO is a hierarchical framework that captures the major axis of the microbial community diversity and is used to assign samples of EMP to its habitat[47]. Therefore, by comparing the result of LEA mapping with each EMPO label, we can estimate the accuracy of environmental prediction by LEA. For each of the lowest layer label (level 3: most specific habitat name) of EMPO, we examined the location of the samples given that label on the LEA map. The results are shown in S11 Fig. Environmental prediction results have well captured the influence of salinity known as the main axis that determines the community structure[2]. For most samples of water, sediments, biofilms, and soils, saline samples were mapped around the ocean area, and non-saline samples were mapped to freshwater or soil area (S11A–S11H Fig). Regarding the samples derived from the host-associated environments, it was observed that the mapping pattern varied depending on the host species even with the same EMPO label. Also, the EMPO label “Plant surface” intuitively evokes leaf surface of land plants, but most of the EMP samples labeled with “Plant surface” mapped to “ocean” area on LEA. This is because most of the EMP samples used in this study with the label “Plant surface” are derived from the kelp as the host (S11J Fig). In such a case, environmental prediction by LEA gives interpretable results (microbial communities on the kelp surface reflects the oceanic community structure pattern, etc.). When the environmental ontology and the community structure pattern seem to conflict, LEA can be used to infer the reason from the mapping results.

### LEA as a semantic search engine for metagenomic samples

The topic-model approach can be used to semantically search documents related to a user’s query[48,49]. By using the trained model parameters in LEA, existing samples can be searched using natural language such as “forest soil”, or “hot spring”. Instead of needing to search for samples by exactly matching the queried words and the description information associated with samples, we can find the sample using latent environmental topics, using the probability of each sample to generate the query sentence as the score of the sample.

As an example, Table 1 shows the top five scoring samples obtained by querying “What kind of microorganisms are in an oil sands tailings pond?” Oil sands tailings ponds are slag ponds accompanying oil sand development and are highly toxic environments as they contain heavy metals, naphtha, bitumen, and other toxic chemicals. Tailing ponds have heterogeneous environments, being aerobic at their surfaces and anaerobic at their bottoms. Many members of the class Methanomicrobia, including *Methanoculleus*, *Methanolinea*, *Methanosaeta*, *Methanobrevibacter*, and *Methanocorpusculum*, which are methanogenic archaea found in the sediment of tailing ponds, contribute to the decomposition of hydrocarbons[50,51]. Given the query, “What kind of microorganisms are in oil sands tailings ponds?”, LEA returned the samples derived from oil sand tailing ponds and oil-water mixtures (Table 1). In addition, LEA returned the sample derived from ocean sediments, although the description of this sample did not contain words such as “oil,” “sand,” or “pond.” Although the microbial community structures of these samples varied in terms of their taxonomy, almost all were composed of methanogenic archaea. Within the machine-learning process, these members of Methanomicrobia are considered simply as variables in the microbial community structure data and their shared characteristics are not recognized (although humans would recognize properties common to all of them given that “Methano-” is at the beginning of each of their names). All high-scoring samples were associated with a large proportion of topic #8 related to methanogenesis (S2 Fig). Therefore, the fact that samples containing many methanogenic archaea were retrieved after querying for “oil sands tailing ponds” indicates that LEA can automatically extract the following two linkages: 1) the association between words such as “hydrocarbon,” “oil,” “tailing,” and “methane,” and the latent environmental topic that represent “methanogenesis,” and 2) the association between methanogenic archaea of various genera and the latent environmental topic that represent “methanogenesis”.

**Table 1:** Top five results for the search query “What kind of microorganisms are in oil sands tailings ponds?”.

Sample accession number	Score^a^	NCBI taxonomy^b^	Abundant genera^c^
DRS011723	4.01471E–06	Marine sediment metagenome	*Methanosaeta* (100%)
SRS151248	4.00920E–06	Hydrocarbon metagenome	*Methanoregula* (57%), *Methanosaeta* (21%), *Methanolinea* (7%)
SRS151247	4.00584E–06	Hydrocarbon metagenome	*Methanoregula* (46%), *Methanosaeta* (31%), *Methanolinea* (7%)
SRS526819	4.00451E–06	Aquatic metagenome	*Methanoplanus* (41%), *Methanocorpusculum* (39%), *Methanolobus* (18%)
SRS151329	4.00358E–06	Hydrocarbon metagenome	*Methanoregula* (64%), *Methanosaeta* (16%)

^a^Scores of each sample were calculated as the probability to generate a query sentence based on topic compositions of each sample.

^b^NCBI taxonomy labels added to each sample. Abbreviation: NCBI, National Center for Biotechnology Information.

^c^Taxonomy names of abundant genera and their relative abundances in the taxonomic composition of the sample.

## Discussion

For this study, we applied a correspondence topic model to more than 30,000 samples of microbial community structure data and extracted the latent environments of each sample as topics. By doing so, we obtained microbial sub-communities that can be regarded as “base variables” to describe an entire dataset and associated word subsets that characterize the environments corresponding to the base variables. By visualizing each sample, which is expressed as a linear combination of these base variables, in two-dimensional space, LEA clarifies continuous variation of the microbial community structures linking two or more environments. The difference between continuously connected environments and an isolated environment might mean that only a few samples have been characterized that bridge the isolated environments. Such environments currently include wastewater, the phyllosphere, and environments related to insect symbiosis. Conversely, human-related environments have been vigorously sampled, and therefore we believe that the visualization reported in this manuscript represents a nearly complete picture of those environments related to healthy human adults.

Using the extracted environmental topics, LEA can infer what mixture of core environments influenced the taxonomic compositions of newly acquired samples. In the river microbiome analysis, we showed that samples taken from the brackish water area of the Tamagawa river can be expressed as a mixture of a “freshwater” topic, a “seawater” topic and a “wastewater” topic. Environmental prediction of new samples is performed by a Bayesian approach similar to that used in a microbial source tracking algorithm[52], but using topic sub-communities extracted from a large-scale dataset as source communities, instead of using the samples pre-specified by a user as sources. This allows to compare new samples virtually with tens of thousands of samples related to diverse natural environments and human body sites. Environmental prediction of new samples may be done by fixing the granularities of environmental labels to be used and comparing with samples to which those labels are added in advance[53]. In such a method, however, it is difficult to set the level of granularities, especially when there are multiple structural patterns of microbial communities in a single environment. When analyzing the dynamics of community structures in a single environment, for example, the time series analysis of human gut microbiome or the spatial distribution of river microbiome, it is more useful to use fine-grained environmental labels than to use simple labels such as “river” or “human gut”. Our method clarifies the structural patterns naturally existing in various environments and provides the way to evaluate how new samples transit among them.

By using a neural network algorithm that maps the data to a two-dimensional space, LEA can position new samples onto the existing global map. This mapping system can be regarded as a microbial global positioning system[54] used to specify the position of a new sample based on the positions of existing sample and allows a user to intuitively evaluate the properties of new samples. Dysbiosis, a deviation from the ordinary distribution of a microbial community structure that exists in symbiosis with humans, has been discussed in relation to diseases, especially those of the gut[55]. Because a newly acquired sample, such as one from an ill patient, can be located anywhere on the map, identifying the ideal end-point from a clinical perspective and defining its vector may be useful information when choosing a specific treatment that can transition its community structure to another state[54].

To perform comparative metagenomics based on environmental information, a huge amount of environmentally labeled data ordered as a dataset is required. However, manually labeling such data is nearly impossible, as the amount of available data is increasing too rapidly at present. In addition, as microbial community structures from new environments are characterized, much work will be needed to design the ontologies of the corresponding environmental labels at the appropriate granularities while incorporating all new environments. Furthermore, because binary environmental labels (presence or absence of an environmental property) are often used to characterize the samples, it is not possible to manually and appropriately evaluate samples that have intermediate properties associated with several environments. Our method automatically extracts the relationship between microbes and their environments by assuming that the microbial community structure and the natural language description for a given sample are both generated from a state in which several environments are mixed. The accuracy of the model should increase as more training data are incorporated.

Prior to extending our method for future work, several problems must be solved. First is how many topics are needed to model microbiomes in highly diverse environments. The number of topics in this study, 80, is an arbitrarily determined value in a sense. In fact, the prediction accuracy of the model for the validation set shows that 80 topics are still inadequate and that a more accurate model can be constructed by setting the more number of topics (perplexity, S1 Fig). However, increasing the number of topics may lead to overfitting, and too large a number of topics may make the map difficult to visualize and interpret. Therefore, we aimed to explain the data with as few topics as possible while keeping the overall prediction accuracy. We are not claiming that microbial communities can be explained by a combination of 80 patterns. The model used in this study is a practical choice to facilitate the interpretation of the whole picture of the microbial community structures and to provide a tool to explore interesting patterns. In the future, as the number of samples acquired from various environments increases, it will be necessary to set a larger number of topics. Nevertheless, from the results of experiments with a large number of topics, the characteristics of the environments considered in this paper (i.e., the gut and vagina) are robust, and an increase in the number of topics would mainly lead to the generation of a topic related to a single research study (e.g., “whale skin” as part of a new topic separated from topic #14, which contains “coral reef”). Second is a sampling bias among environments. Depending on the environment, the number of samples studied so far varies greatly. Since LEA determines topics based on the prediction accuracy of the validation set samples, LEA tends to express an environment with a large number of samples in high resolution by placing many topics in it, while environments with a small number of samples tend to be compressed into a limited number of topics. Currently, the environment related to humans, especially to the intestinal tract, have high resolution, but the environment related to freshwater or hot springs has relatively few samples and the separation of topics can be insufficient (S9 Fig). Therefore, attention should be paid to interpretation concerning LEA predictions for such natural environments in which relatively a few number of samples were studied. By incorporating large-scale data of microbiomes sampled from the natural environment, such as data from the Earth Microbiome Project[47], high-resolution topics will be obtained for those environments in the future. The third problem is, though common to any meta-analysis, a bias due to the difference in experimental methods. In the environmental prediction results of the MBQC dataset by LEA, it was hardly the case that the difference in experimental methods yields a large difference in coordinates on the LEA global map. Nonetheless, depending on the area on the map, such a bias may have the effect of placing the sample close to a particular topic. Ideally, there should be as much data as possible processed in a unified experimental protocol.

We do not know if all topics used for our research specifically express a set of related environmental parameters, except for topics such as those representing “methanogenesis.” In the future, the environmental parameters that determine the microbial community structure will need clarification that can be accomplished by examining the relevance of various metadata and topic compositions and transitions.

## Materials and methods

### Dataset construction

Genus-level taxonomic composition data for 48,873 sequenced 16S rRNA gene amplicon samples were obtained from MicrobeDB.jp (http://microbedb.jp/MDB/), which is an integrated, publically available database for microbes in which all metagenomic data registered in the International Nucleotide Sequence Database Collaboration Sequence Read Archive (SRA) prior to August 2014 are stored. MicrobeDB.jp includes reanalyzed taxonomic compositions of all samples using the unified analysis pipeline. Briefly, for all original sequence data registered in the SRA, we trimmed adapter sequences and filtered out low-quality sequences, sequences derived from the PhiX genome, and sequences derived from the human genome. For all high-quality sequences in each sample, we searched for similar sequences in the VITCOMIC2[29] database (http://vitcomic.org) using CLAST[56] and then performed taxonomic assignments on these sequences. Finally, the number of sequences assigned to a specific genus were summed for each sample. As a result, we obtained a dataset for which each SRS ID (identifier associated with a sample in SRA) had a genus-level taxonomic composition. All samples with <1,000 sequences with an assigned genus were discarded, and samples with >10,000 assigned sequences were subsampled as 10,000 sequences.

To obtain metadata for the samples, XML files for the SRS IDs registered prior to August 2014 were downloaded from the SRA database. The XML file for each SRS ID contains descriptions of the properties of the corresponding sample, such as pH values and a description of the environment from which the sample was obtained. We extracted the text sandwiched between all tags (e.g., research titles, scientific names, geographical locations, and sample descriptions) in those XML files, lemmatized all words, and organized them according to a bag-of-words model (with counts of the number of times each word appears). We removed all English stop words from the extracted text. Additionally, all words corresponding to any of the following conditions were removed: 1) words that contain numbers or symbols that are not alphabetic (in many cases these were the project-specific sample identifiers); 2) words in which the letters “A”, “T”, “C”, or “G” occupied ≥70% of the word length (often tag sequences or primer sequences); 3) words generally used in many samples, e.g., genome and metagenome (S2 Table); 4) words used only for a single research study; and 5) words that appeared <20 times or in >30% of the samples in the dataset. Samples with all words removed according to the aforementioned conditions were themselves discarded.

Finally, we integrated the taxonomic composition and sample description datasets to construct a dataset consisting of only samples containing both features. This dataset contains 30,718 samples, each of which has a taxonomic composition (a count of each genus in the sample) and a description of the properties of the sample (bag-of-words). The number of genera in the final dataset (vocabulary of taxonomic composition data; S3 Table) is 1675, and the number of unique words in the dataset (vocabulary of sample description data; S4 Table) is 764. The final dataset is publicly available at http://palaeo.nig.ac.jp/Resources/lea2018/.

### Corr-LDA model inference

We assumed that each sample had a multinomial distribution of latent environments and that both the observed genera and the sample description document were generated according to the mixing ratio of those environments or topics. To infer topics, we applied Corr-LDA[25,26] to the dataset.

In our LEA system, Corr-LDA model was applied to our dataset as described below. Each sample *d* (*d* = 1 …*D*) has taxonomic composition data *w*_*d*_ = {*w*_*dn*_} (n = 1…*N*_*d*_) and description data *t*_*d*_ = {*t*_*dm*_} (m = 1…*M*_*d*_). Here, *N*_*d*_ is the number of sequences with an assigned taxonomy in sample *d*, *w*_*dn*_ is the taxonomy assigned to the *n*th sequence in sample *d*, *M*_*d*_ is the number of words in the description given to sample *d*, and *t*_*dm*_ is the *m*th word in sample *d*. In a topic model, it is assumed that all elements in the data have a latent topic. The latent topic of the *n*th sequence in sample *d* is defined *z*_*dn*_ ∈ {1 …*K*}, and *K* is the number of latent topics that have been pre-specified. The latent topic of the *m*th word in sample *d* is defined *c*_*dm*_ ∈ {1 …*K*}, with *K* again representing the number of latent topics, which is the same for both sequences and words. Topics assigned to each sequence and each word is undefined prior to analysis and inferred using the entire dataset. For the entire dataset, the joint probability distribution for the taxonomic composition data *W*, the description data *T*, the latent topics *Z* for sequences, and the latent topics *C* for words was written as follows:

P(W,T,Z,C|α,β,γ)=P(Z|α)P(W|Z,β)P(C|Z)P(T|C,γ)

where α, β, and γ are hyper-parameters of prior distributions for topics, taxa, and words, respectively.

We assumed that latent topics *Z* for genera appearing in each sample *d* is generated according to the multinomial distribution θ_*d*_ and that the prior of θ_*d*_ is the asymmetric Dirichlet distribution having α_*z*_ (*z* = 1 …*K*) as hyper-parameters. θ_*d*_ can be integrated out and P(Z|α) can be written as follows:

$$P(Z|\alpha )=\prod_{d=1}^{D}\int P(Z_{d}|\theta_{d})P(\theta_{d}|\alpha )d\theta_{d}={\left(\frac{\Gamma (\sum_{z=1}^{K}\alpha_{z})}{\prod_{z=1}^{K}\Gamma (\alpha_{z})}\right)}^{D}\prod_{d=1}^{D}\frac{\prod_{z=1}^{K}\Gamma (N_{zd}+\alpha_{z})}{\Gamma (N_{d}+\sum_{z=1}^{K}\alpha_{z})}$$

where *N*_*zd*_ is the number of sequences that are assigned to a topic *z* in sample *d* and Г is the gamma function.

The genera appearing in a sample is generated according to a multinomial distribution φ_z_ when its latent topic is *z*. φ_z_ can be interpreted as a sub-community of genera associated with latent topic *z*. We assumed the symmetric Dirichlet prior for φ_z_. φ_z_ can also be integrated out and P(*W*|*Z*, *β*) can be written as follows:

$$P(W|Z,\beta )=\prod_{z=1}^{K}\int P(W|z,\varphi_{z})P(\varphi_{z}|\beta )d\varphi_{z}={\left(\frac{\Gamma (\beta V)}{{\Gamma (\beta )}^{V}}\right)}^{K}\prod_{z=1}^{K}\frac{\prod_{w=1}^{V}\Gamma (N_{zw}+\beta )}{\Gamma (N_{z}+\beta V)}$$

where *N*_*zw*_ is the number of sequences assigned to genus *w* with a topic assigned to *z*, *N*_*z*_ is the number of sequences assigned to a topic *z*, and *V* is the number of unique genera in the dataset.

Topics for words in sample description data were generated according to the following:

$$c_{dm}∼Multinomial\left({\{\frac{N_{zd}}{N_{d}}\}}_{z=1}^{z=K}\right)$$

where *N*_*d*_ is the number of genus-assigned sequences in sample *d*. Thus, topics for words are conditional on topics assigned for genera.

A word appearing in a sample is generated according to the multinomial distribution ψ_c_ when its latent topic is *c*. ψ_c_ can be interpreted as a subset of words representing latent topic *c*, and we assumed the symmetric Dirichlet prior for ψ_c_. As in the case of P(*W*|*Z*, *β*), P(T|C,γ) can be written as follows:

$$P(T|C,\gamma )=\prod_{z=1}^{K}\int P(T|c,\psi_{c})P(\psi_{c}|\gamma )d\psi_{c}={\left(\frac{\Gamma (\gamma T)}{{\Gamma (\gamma )}^{T}}\right)}^{K}\prod_{z=1}^{K}\frac{\prod_{t=1}^{S}\Gamma (M_{zt}+\gamma )}{\Gamma (M_{z}+\gamma S)}$$

where *M*_*zt*_ is the number of words *t* with a topic assigned to *z*, *M*_*z*_ is the total number of words assigned to a topic *z*, and *S* is the number of unique words in the dataset.

We assumed the asymmetric Dirichlet prior only for the topic multinomial distribution, and the symmetric Dirichlet prior for the genera and word multinomial distributions because samples for which their microbial community structure had been analyzed previously are more likely to have been acquired from human symbiotic environments, suggesting that a bias might also exist for the topic occurrence probabilities. It was reported that setting an asymmetric Dirichlet prior for a topic distribution is effective for robust inference of a topic model[57].

The posterior distributions of the latent topic *Z* for genera and the latent topic *C* for words were approximated by the collapsed Gibbs sampling method[26,58]. Hyper-parameters (α, β, and γ) were updated in each step during the Gibbs sampling by the fixed-point iteration method[59].

To determine the number of topics *K*, we randomly divided the dataset into 25,718 samples as a training set and a group of 5,000 samples as a test set, and then the test set perplexity was calculated using the results from the training set with varying numbers of topics. Perplexity is an index for measuring the predictive performance of a held-out test set, and the smaller the value, the better the performance. We ran five Markov chains using different initial values and inferred the model parameters. Next, using the model parameters inferred from the training set, the topic composition of each sample in the test set was estimated using 50% of the sequences in each sample, and the generation probability of genera assigned to remaining 50% of the sequences was calculated. S1 Fig shows the average perplexities and standard deviations obtained by inference with five Markov chains for 5 to 300 topics. Setting too large a number of topics reduces the interpretability of the training results and might cause overfitting for existing samples, so we fixed the number of topics as 80.

Finally, we ran a Markov chain using 30,718 samples with the number of topics set at 80 and acquired the topic composition θ for each sample, the genera probability φ for each topic (microbial sub-community in each topic), and the word probability ψ for each topic (word subset corresponding to each topic) when the joint likelihood converged after a sufficient number of Gibbs iterations. The implementation of Corr-LDA used in this study is available at https://github.com/khigashi1987/CorrLDA. Parameters used were -I 1000 -K 80 (1000 Gibbs iterations and 80 topics).

Word subsets and microbial sub-communities associated with each topic are listed in S2 and S3 Figs. All word-cloud images were generated using the word-cloud generator in Python (https://github.com/amueller/word_cloud).

The structure of sub-communities differs greatly between most topic pairs (S6A Fig). The topic pair with the most similar sub-communities is formed by the soil-environment topics #19 and #54, although the structures of these topics are still greatly different (S6B Fig). Similar comparison on word subsets of topics shows that there are many more similar topic pairs with respect to word probabilities (S6C Fig). The topic pair with the most similar word subsets is formed by the vagina-associated topics #43 and #52 (S6D Fig). In both topics, two words, “female” and “vaginal”, dominate greatly in their probabilities and reflect similar environmental concepts (“vagina”). However, in a topic #52, two words, “african” and “american”, have relatively large probabilities, whereas in a topic #43 the probabilities of those words are very small (S6D Fig). Unlike microbial sub-communities of topics, it is natural that there are multiple similar topics for word-subsets. That is because there are some environments which have different patterns of the community structures but are difficult for the human to distinguish and describe those, as with the case of the human gut environment (enterotypes[7]). Nonetheless, like these vagina topics, some topics may reflect slight differences that appear in the sample descriptions.

As for what kinds of environment the topics show, it is strongly influenced by the sampling bias by the previous research. The vigorously studied environment can be modeled at the high resolution, resulting in a large number of topics associated with the environment. We investigated the number of topics related to a specific environment by calculating the generation probability of specific words for each topic. If the topic had a probability of generating the word “gut” above 5%, we considered that topic to be related to the gut-associated environment. Similarly, we calculated the sum of the generation probabilities the words “oral”, “cavity”, “dental”, “plaque”, “gingiva”, “tonsil” and “saliva” for the oral cavity-associated environment, the word “skin” for the skin-associated environment, the words “vaginal” and “vagina” for the vagina-associated environment, the words “marine”, “sea”, “ocean”, “seawater” and “saline” for the ocean-associated environment, the words “freshwater”, “lake” and “river” for the freshwater-associated environment, the words “soil”, “agricultural” and “field” for the soil-associated environment, and the words “hot” and “spring” for the hot spring-associated environment for each topic. As a result, there were many topics associated with the gut environment (22 topics), and the number of topics related to the natural environment tended to be small (S8 Fig). When examining the number of samples with more than 50% of each “environment-associated topics”, similar trends were observed (S9 Fig).

### Visualizing the model parameters

After the above process, all samples in the dataset were expressed as 80-dimensional, real-valued vectors showing topic compositions. In general, for visualization of 80-dimensional vectors, it is effective to arrange sample points in two or three-dimensional space by dimensionality reduction, and various dimensionality reduction methods, such as principal component analysis or the multidimensional scaling method, can be applied. For this approach, we used t-SNE (t-distributed Stochastic Neighbor Embedding)[60], which embeds sample points in a low-dimensional space while maintaining the local structures between sample points in the original high-dimensional space. However, simple t-SNE method is inadequate for newly acquired samples. When predicting the topic composition of a new sample and comparing it with existing samples, the distance calculation and the cost minimization for the entire dataset must be performed again with the new sample, which might cause a random change of the coordinates in the low-dimensional space each time a new sample is added. Therefore, we adopted the parametric t-SNE method[31], which trains a function with the same behavior as t-SNE using a neural network. This neural network inputs an 80-dimensional vector and outputs two-dimensional coordinates. Weights of the neural network are trained with the same loss function as for the normal t-SNE. We constructed a four-layer, feed-forward neural network (the number of nodes is 80, 160, 160, 640, and 2). We used the Rectified Linear Unit as the activation function of the nodes in all layers except the last one and the linear activation function in the last layer and trained the weights using the mini-batch stochastic gradient descent method. We implemented parametric t-SNE by Theano[61] and Keras (https://github.com/fchollet/keras) and used Adam[62] as the optimizer. After a sufficient number of epochs, we obtained the coordinates of all samples by inputting topic compositions of samples into the neural network and then visualized samples in a scatter plot. At the same time, we obtained the coordinates of topics in two-dimensional space by inputting one-hot vectors (80-dimensional vectors with a single 1 value and 0 for all other values) into the neural network. On those coordinates in a scatter plot, we mapped pictures corresponding to the word subset of each topic. Thus, the sample plotted in a position close to a given picture means that the mixing ratio of that topic is very high for the sample. All pictures used in this study are in the public domain.

### Estimation of topic compositions for new samples

The topic composition of a new sample was estimated using the taxonomic composition of the new sample, the hyper-parameter α, and the genera generation probability φ, learned from the training samples. First, the DNA sequence data from a new sample was analyzed by VITCOMIC2 and converted into taxonomic composition data. The estimation of the topic composition in a Bayesian approach requires consideration of all possible assignments of sequences to topics, but direct estimation of the posterior distribution of the topic composition is intractable[52]. In LEA, the posterior distribution of the topic composition of a new sample is approximated by performing Gibbs sampling with randomly initializing topics assigned to sequences in the new sample. For the genus *w* assigned to the *n*th sequence of a new sample *d*, the latent topic *z* was sampled according to the following conditional distribution:

$$P(z_{dn}=k|W,Z_{\setminus dn},\varphi ,\alpha )\propto \varphi_{kw}\frac{N_{kd\setminus dn}+\alpha_{k}}{N_{d\setminus dn}+\sum_{z=1}^{K}\alpha_{z}}$$

where φ_*kw*_ is the generative probability of genus *w* when *z*_*dn*_ is *k*, α_*k*_ is the hyper-parameter of the topic probability; *N*_*kd\dn*_ is the number of sequences assigned to topic *k*, except for the *n*th sequence in sample *d*; and *N*_*d\dn*_ is the total number of sequences in sample *d* minus one. This distribution is similar to the sampling formula used in SourceTracker[52], but LEA uses microbial sub-communities of 80 topics as source communities, instead of using a sample set specified by the user as the source in SourceTracker. LEA estimates the topic composition of the new sample using only sequence information because we assume that the primary use of LEA is to predict the environment of the sample for which description information is not available, or, more importantly, to predict contamination of the unexpected environment. After a sufficient number of Gibbs-sampling iterations, the topic composition of the new sample was expressed as an 80-dimensional vector and converted to the coordinates on a two-dimensional map by the feed-forward neural network learned with training samples. By arranging the transformed coordinates of the new sample on the same two-dimensional map as the existing samples, we could compare the features of the new sample with those of all samples in our database.

We provide REST (representational state transfer)-style APIs (application programming interfaces) to access all the model parameters of LEA and the environmental prediction function of the user sample at http://snail.nig.ac.jp/leaapi/. With the HTTP POST method, environmental predictions for the new samples can be calculated from the command line interface without going through the web application. There is also an API that provides information on the instability of the posterior distribution calculation of topic proportions for the new samples. The function computes topic proportions by drawing 100 samples from 100 independent Gibbs sampling chains with different initial settings and by averaging 100 samples. This also provides standard deviations of each topic proportion estimates. These values can be regarded as “goodness of fit” of the new samples to the LEA model. In the calculation results on the Tamagawa river microbiome, the topic proportions shown in Fig 2 was stable at any point (S7 Fig).

### Implementation of LEA as a web application and a semantic search engine of metagenomics samples

The training results from the 30,718 samples and their visualization are available as an interactive web application at http://leamicrobe.jp. The back end of the application is written in C language and Python, and the front end is written in JavaScript. Users can examine the taxonomic and topic compositions of each sample, and the sub-community and word subset of each topic on the global map. By uploading the taxonomic composition data obtained via VITCOMIC2, it is possible to place user samples on the global map after several seconds of calculation for estimating topic compositions. Because LEA accepts only taxonomic composition data generated by VITCOMIC2, VITCOMIC2 must be used to obtain taxonomic compositions from raw sequence data.

Furthermore, with the use of the training results in our model, it is possible to perform a sample search that does not depend on exact matching between the query text and sample metadata. The sample-retrieval process uses the word generation probability ψ in each topic and the topic composition θ in each sample as learned with the existing samples. The search query consists of free words, i.e., several English words or English sentences. First, the search query is divided into words. If the search query contains words that do not exist in the vocabulary of LEA (764 words), those words are simply discarded. LEA also carries out preprocessing of query words (removal of English stop words and lemmatization) same as the training step of LEA model. After that, the score of sample *d* is calculated using the following equation:

$$Score(d)=P(q|d)=\prod_{n=1}^{N}\sum_{z=1}^{K}P(q_{n}|z)P(z|d)=\prod_{n=1}^{N}\sum_{z=1}^{K}\psi_{zq_{n}}\theta_{dz}$$

where *q* = {*q*_*n*_} (*n* = 1 to *N*) is a search-word set and *N* is the number of valid words in the search-word set. This means that we use the probability that the sample *d* generates the search query *q* as the score of the sample *d*. LEA calculates scores of all samples in LEA dataset (30,718 samples) and sorts them in descending order. By entering free words in the search window of the web application, it is possible to highlight samples with a high score, meaning that those samples are semantically related to the search query. If the taxonomy name of a microbe (or its synonym(s)) is included in the search query, the score is scaled according to the abundance of that microbe in each sample. If the search query contains the name of higher taxa than a genus level, the score is scaled according to the sum of abundances of microbes below the input taxa (taxonomy based on NCBI Taxonomy). This makes it possible to search samples by queries such as “Gemmatimonadetes in the ocean”.

### Data preparation for LEA mapping

As examples that show how LEA mapping can be used, we acquired four microbiome datasets. 1) Intestinal microbiota data from two subjects over the course of a year[40]. 2) Intestinal microbiota data obtained by various DNA extraction methods from Microbiome Quality Control Project[42]. 3) Microbiota data from various natural environments from Earth Microbiome Project[47]. 4) Microbiota data from the upper region to the estuary of the river Tamagawa.

The intestinal microbiota data are those reported by David *et al*. (PRJEB6518)[40]. In that study, every day for all or most of a year, the microbial 16S rRNA genes from the feces of two healthy adult men (subjects A and B) were PCR amplified and sequenced. The sequence data were downloaded from the European Nucleotide Archive (ENA), and the taxonomic compositions of the samples were analyzed by VITCOMIC2. LEA was used to predict the topic compositions from the taxonomic composition data and input the topic compositions into the neural network to obtain coordinates of the samples on the global map. For the topic composition prediction, samples with <1,000 sequences assigned to genera were discarded, and samples with >10,000 sequences were sub-sampled to 10,000 sequences. For comparison, the dataset of Japanese gut microbiome samples by Nishijima *et al*. (PRJDB3601)[63] was also downloaded, their taxonomic compositions were estimated by VITCOMIC2, their topic compositions were estimated by LEA, and the samples were mapped on the global map. The number of gut samples was 314 for subject A, 184 for subject B, and 265 for the Japanese population.

To assess the impact of different DNA extraction methods of the human gut microbiome analysis on the locations on LEA global map, we used the Microbiome Quality Control (MBQC) dataset reported by Sinha *et al*. (PRJNA260846)[42]. This dataset contains 16S amplicon sequencing data from human stool samples, chemostats, and artificial microbial communities. For the same biological sample, there are multiple sequencing data analyzed with different wet laboratories or different DNA extraction methods. The sequence data were downloaded from the SRA, and the taxonomic compositions of the samples were analyzed by VITCOMIC2. LEA was used to predict the topic compositions and obtain coordinates of the samples on the global map. For the topic composition prediction, samples with <1,000 sequences assigned to genera were discarded, and samples with >10,000 sequences were sub-sampled to 10,000 sequences. The number of samples was 1558 for human stool samples, 228 for chemostats, 133 for fecal artificial communities, and 130 for oral artificial communities.

For the microbiome data from the highly diverse natural environments, we used the subset of Earth Microbiome Project (EMP) dataset[47]. The original dataset of EMP contains more than 27,000 samples of 97 studies, but they also provide the subset of the sample list which gives as equal as possible representation across EMP Ontology (EMPO)-level 3 sample types and across studies within those sample types. We downloaded the table of sample identifiers for the EMP subset of 2,000 samples (emp_qiime_mapping_subset_2k.tsv) from the FTP site of the EMP project (ftp://ftp.microbio.me/emp/release1). Raw sequence data of each sample were downloaded from the ENA, and the taxonomic compositions of the samples were analyzed by VITCOMIC2. LEA was used to predict the topic compositions and obtain coordinates of the samples on the global map. For the topic composition prediction, samples with <1,000 sequences assigned to genera were discarded, and samples with >10,000 sequences were sub-sampled to 10,000 sequences. The number of samples used in the LEA mapping was 1760.

For the spatial microbiome distribution, we sampled the Tamagawa river in Japan. Its source is at the peak of Mt. Kasatori, and it flows through the Yamanashi, Tokyo, and Kanagawa prefectures. The river’s basin area is 1,240 km^2^, its total length is 138 km, and the altitude of its source is 1,953 m. We sampled the river’s water on May 26 and 27, 2015 at 38 points from its upper region to its estuary (S5 Fig, S1 Table). Sampling was carried out during sunny weather and no precipitation had occurred for the 4 days before sampling. Samples were collected from the river’s surface water (defined as flowing water no deeper than ~30 cm from the river surface) using a ladle at the riverbank without disturbing the sediment. At least 500 ml of water was sampled at each site and then immediately injected into a sterile polypropylene bottle. Samples were transported in an insulated box containing a refrigerant and were moved to a refrigerator at 4°C within the day. The water of each sample was filtered through a 0.2-μm membrane within 5 days of sampling. Each filter was quickly placed in a sterile tube and stored frozen at –20°C. DNA was extracted from the material on each filter using the PowerWater DNA Isolation Kit (MO BIO Laboratories, Carlsbad, CA) with bead homogenization on a Micro Smash MS-100R (TOMY, Tokyo) at 3,000 rpm for 30 s. PCR amplification was performed using the primers 342F and 806R, which are V3–V4 region universal primers for prokaryotic 16S rRNA genes[64], and Ex Taq DNA polymerase (Takara, Shiga, Japan) with a denaturation step at 98°C for 2 min, followed by 30 cycles at 98°C for 10 s, 50°C for 30 s, and 72°C for 40 s, and a final extension step at 72°C for 10 min. PCR products were purified with the Agencourt AMPure XP system (Beckman Coulter) and sequenced using an Illumina MiSeq sequencer (Illumina, San Diego, CA, USA). The taxonomic composition of each sample was analyzed by VITCOMIC2, and topic compositions for the samples were predicted by LEA. The Tamagawa river microbiome data has been deposited in DDBJ BioProject database; accession number: PRJDB5936.

## Supporting information

S1 Fig Relationship between the perplexity value of the model and the number of topics. The perplexity of a 5,000-microbe sample test set was calculated using results from the Corr-LDA model inference for the training set with different numbers of topics. For each number of topics, the average value of the calculated perplexities from five independent Markov chains is shown. The error bars represent the standard deviation. The perplexity decreased monotonically as the number of topics increases, and ~80–100 topics are sufficient to obtain a sufficiently small perplexity value.
(PDF)

S2 Fig Word sets and microbial sub-communities for topics #0 - #39. For each of the topics, the picture used in the LEA global map, 20 words with the highest generation probabilities, and the pie chart for the genus generation probability are shown. In each pie chart, the genera with the 15 highest generation probabilities are color-coded by their phylum level taxonomy, and other less probable genera are in gray.
(PDF)

S3 Fig Word sets and microbial sub-communities for topics #40 - #79. (PDF)

S4 Fig **LEA mapping of the time series of gut microbiome samples from subjects A and B on the global map.** The maps of the daily gut microbial community structures retrieved from two individuals are shown (data from David *et al*.; PRJEB6518). Most of the samples were mapped to “the area of the gut” as learned by pre-existing samples. (Top) The color gradation represents when the samples were taken from the gut of each subject. (Bottom) The samples (colored dots) on the map are colored according to their associated times. Samples during a particular event (traveling and infection) are highlighted by the star-shaped plots. The trajectory of samples is indicated by a dotted line. The dark gray dots on the map represent the samples used for training. (A) The map of the gut microbiome of subject A. The gut environment of subject A was very stable and had a similar taxonomic composition and topic composition over much of the year. However, this stability was disturbed when subject A traveled from his home in an urban area in the United States to Southeast Asia where he stayed from day 72 to day 122 and ate the local diet. Most of the samples that lay outside the cluster are those collected during the subject’s stay in Southeast Asia. We assume that the disturbance of the gut microbiome of the subject during his stay in Southeast Asia reflects his exposure to the local diet and environment. As a comparison, gut microbiomes taken from 106 healthy Japanese subjects are mapped as pink dots (data from Nishijima *et al*.; PRJDB3601). Interestingly, at the beginning of subject A's stay in Southeast Asia, the positions of some of his samples were near those of the Japanese subjects (Day 87). (B) The map of the gut microbiome of subject B. The topic composition of the subject was stable and near topic #28 during the first half of the year. However, because subject B was infected with Salmonella between days 151 and 159, the microbial sample taken on the day 155 mapped near topic #68. After recovery from the infection, subject B's samples were nearer to topic #16.
(PDF)

S5 Fig Locations where water from the Tamagawa river was sampled. The red points identify the sampling sites of the surface water of the Tamagawa river. Black lines indicate drainage systems around the Tamagawa river. This figure is created based on the base map data downloaded from Geospatial Information Authority of Japan.
(PDF)

S6 Fig Evaluation of similarities of microbial sub-communities between topics. (A) Similarities of topics were assessed by the Jensen-Shannon divergence of genera probabilities between pairs of the 80 topics. Most of the values were distributed near or at 1.0, which represents maximum divergence. The lowest value, identified by the arrow, was 0.3970 for topics #19 and #54. (B) The probabilities of genera among the most similar pair of topics, topics #19 and #54. (C) Similarities of topics were assessed by the Jensen-Shannon divergence of word probabilities between pairs of the 80 topics. Compared to the case of genera probabilities, there are many more similar topic pairs in word probabilities. The lowest value, identified by the arrow, was 0.0490 for topics #43 and #52. (D) The probabilities of words among the most similar pair of topics, topics #43 and #52.
(PDF)

S7 Fig Stability of the topic proportion calculations for the Tamagawa river microbiome. Topic proportions were calculated by drawing 100 samples from 100 independent Gibbs sampling chains with different initial settings. Bars represent the average values and the error bars represent standard deviations of 100 samples. Topic proportions were stable among different chains at any point on the Tamagawa river.
(PDF)

S8 Fig Topics associated to specific environments. Each bar represents a probability of the environment associated words of each topic. Topics that can generate a specific word with above a probability of 5% (red line) was decided as environment-associated topics.
(PDF)

S9 Fig The bias of the number of samples associated with each environment. The vertical axis shows the proportion of environment-associated topics per sample, and the horizontal axis shows 30,718 samples. Samples were ordered by the proportion of the corresponding topics.
(PDF)

S10 Fig LEA mapping of microbiome quality control dataset. LEA mapping of Microbiome Quality Control (MBQC) dataset. (A) Colored crosses represent all 2,049 samples of Microbiome Quality Control Project on the LEA global map. These contain human-derived samples (pink), chemostat samples (light blue), artificial fecal communities (green), and artificial oral communities (orange). (B-S) LEA mapping results of samples for each subject. Colored crosses indicate samples derived from each subject. Different colors indicate the samples obtained with different DNA extraction methods.
(PDF)

S11 Fig LEA mapping of earth microbiome project dataset. LEA mapping results of 2,000 subset of Earth Microbiome Project (EMP) dataset. Groups of samples were separately displayed according to their EMP Ontology-Level 3 labels. (A-Q) Colored crosses indicate EMP samples. In panels J, K, M, N, O, P, and Q, samples were colored by their host species.
(PDF)

S1 Table Sampling sites of the Tamagawa river. (XLSX)

S2 Table Additionally removed words. (XLSX)

S3 Table Vocabulary of taxonomic composition data. (XLSX)

S4 Table Vocabulary of sample description data. (XLSX)
