# Supplementary figures and images for: Latent environment allocation of microbial community data
Source: PLoS Comput Biol. 2018 Jun 6;14(6):e1006143. doi: 10.1371/journal.pcbi.1006143 (PMC6005635; doi:10.1371/journal.pcbi.1006143)

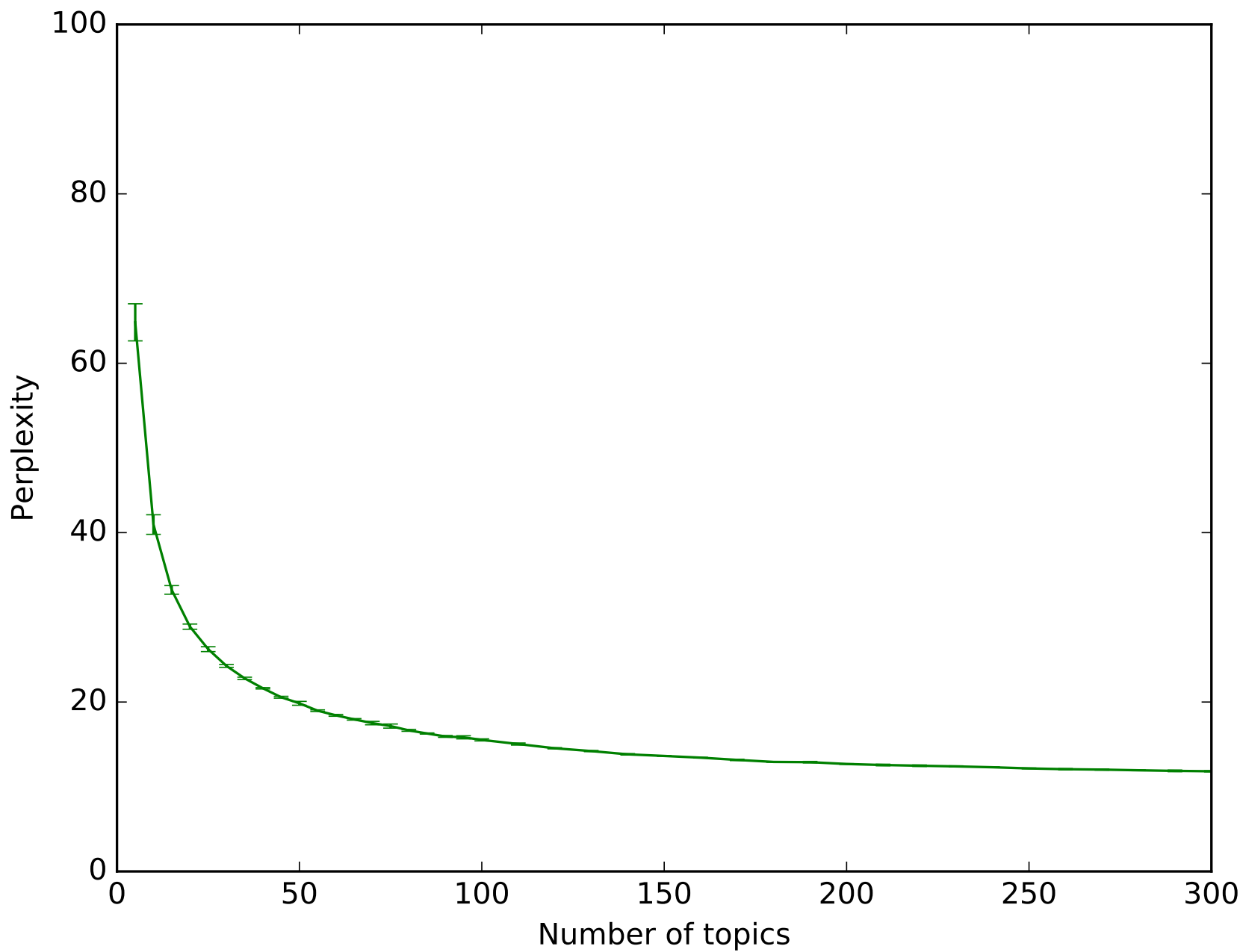

Supplement: S1 Fig — The perplexity of a 5,000-microbe sample test set was calculated using results from the Corr-LDA model inference for the training set with different numbers of topics. For each number of topics, the average value of the calculated perplexities from five independent Markov chains is shown. The error bars represent the standard deviation. The perplexity decreased monotonically as the number of topics increases, and ~80–100 topics are sufficient to obtain a sufficiently small perplexity value. (PDF) [file pcbi.1006143.s001.pdf]

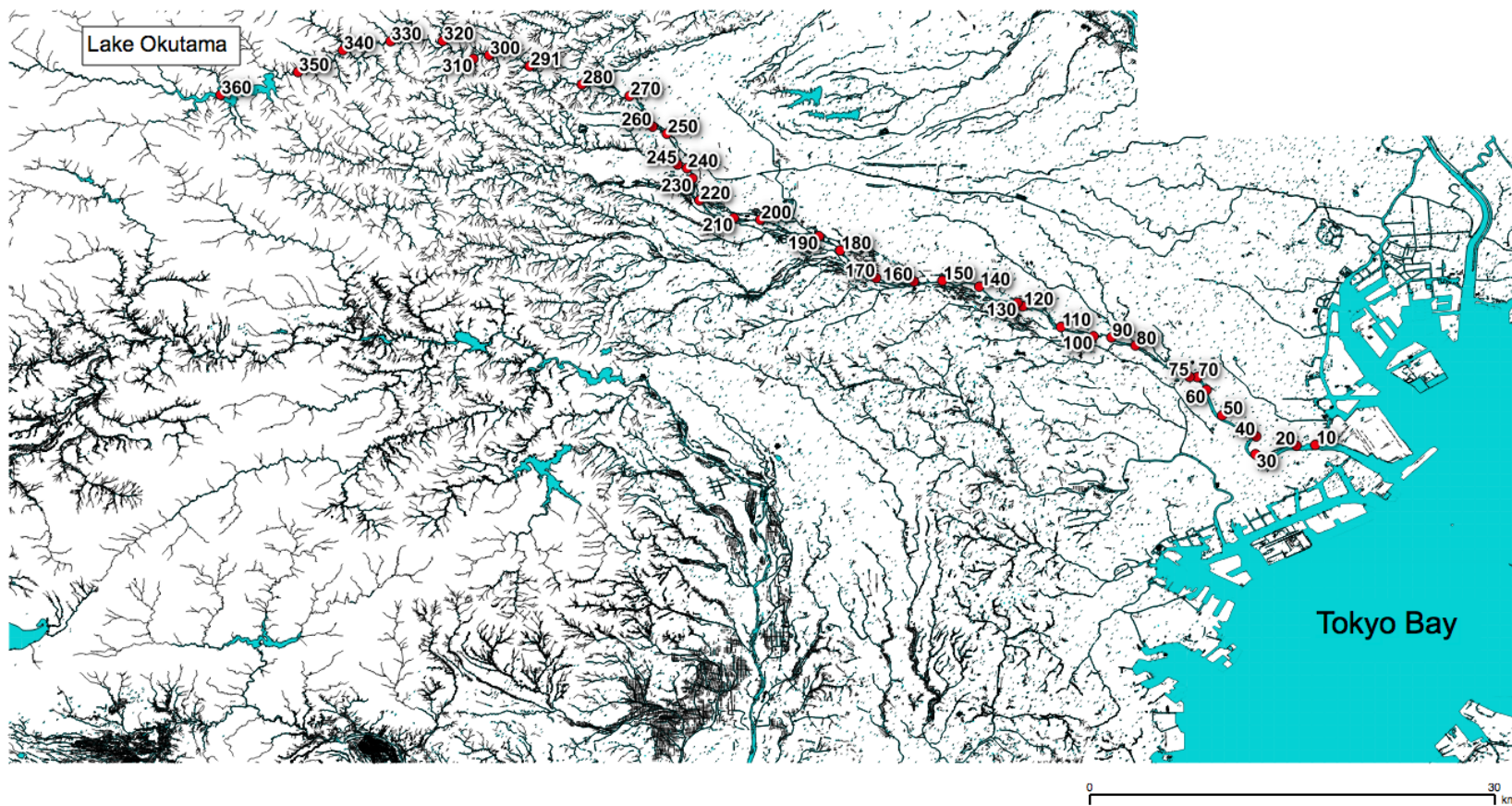

Supplement: S5 Fig — The red points identify the sampling sites of the surface water of the Tamagawa river. Black lines indicate drainage systems around the Tamagawa river. This figure is created based on the base map data downloaded from Geospatial Information Authority of Japan. (PDF) [file pcbi.1006143.s005.pdf]

**A**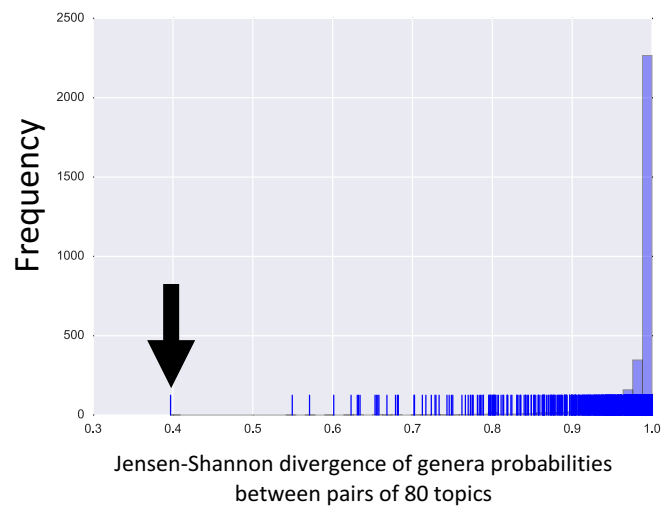**B****Topic #54**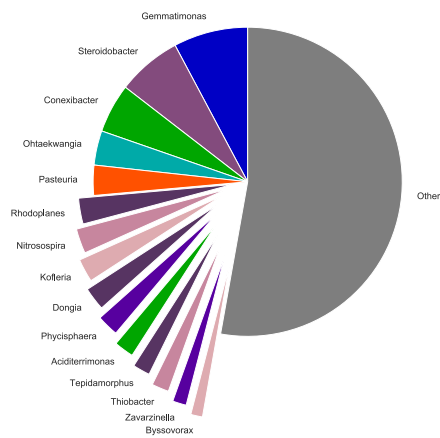**Topic #19**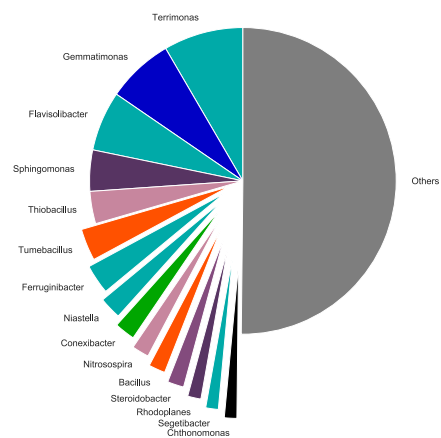**C**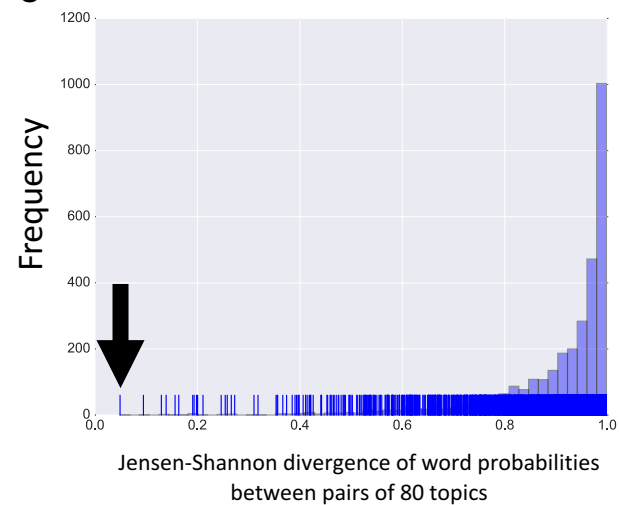**D****Topic #43**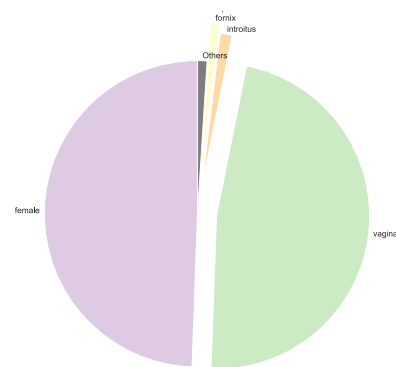**Topic #52**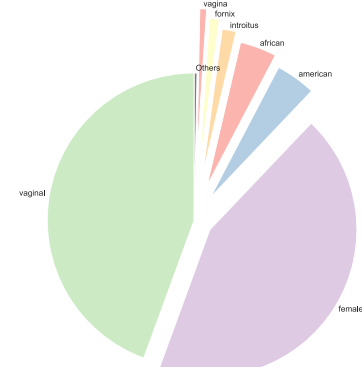

Supplement: S6 Fig — (A) Similarities of topics were assessed by the Jensen-Shannon divergence of genera probabilities between pairs of the 80 topics. Most of the values were distributed near or at 1.0, which represents maximum divergence. The lowest value, identified by the arrow, was 0.3970 for topics #19 and #54. (B) The probabilities of genera among the most similar pair of topics, topics #19 and #54. (C) Similarities of topics were assessed by the Jensen-Shannon divergence of word probabilities between pairs of the 80 topics. Compared to the case of genera probabilities, there are many more similar topic pairs in word probabilities. The lowest value, identified by the arrow, was 0.0490 for topics #43 and #52. (D) The probabilities of words among the most similar pair of topics, topics #43 and #52. (PDF) [file pcbi.1006143.s006.pdf]

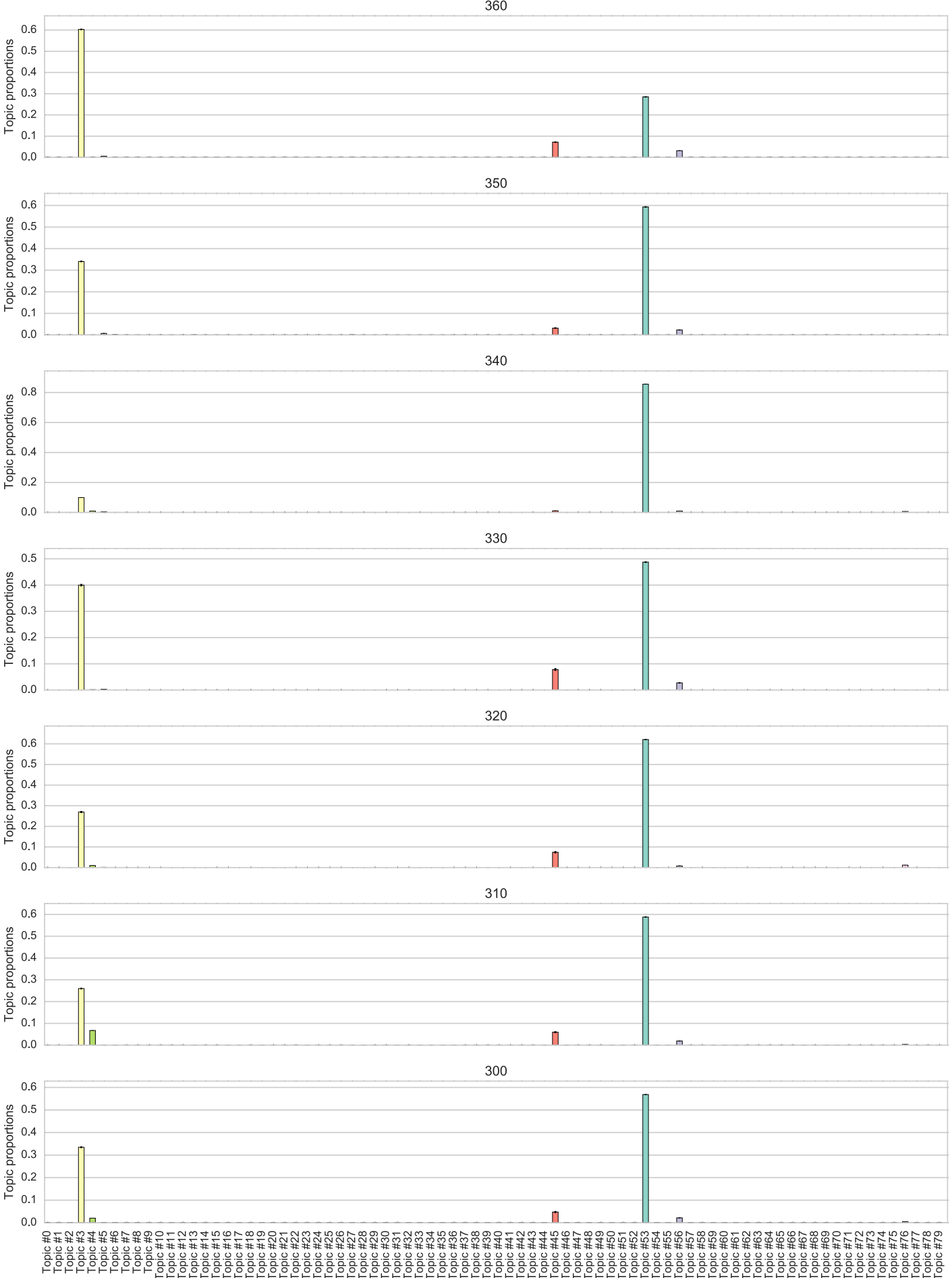

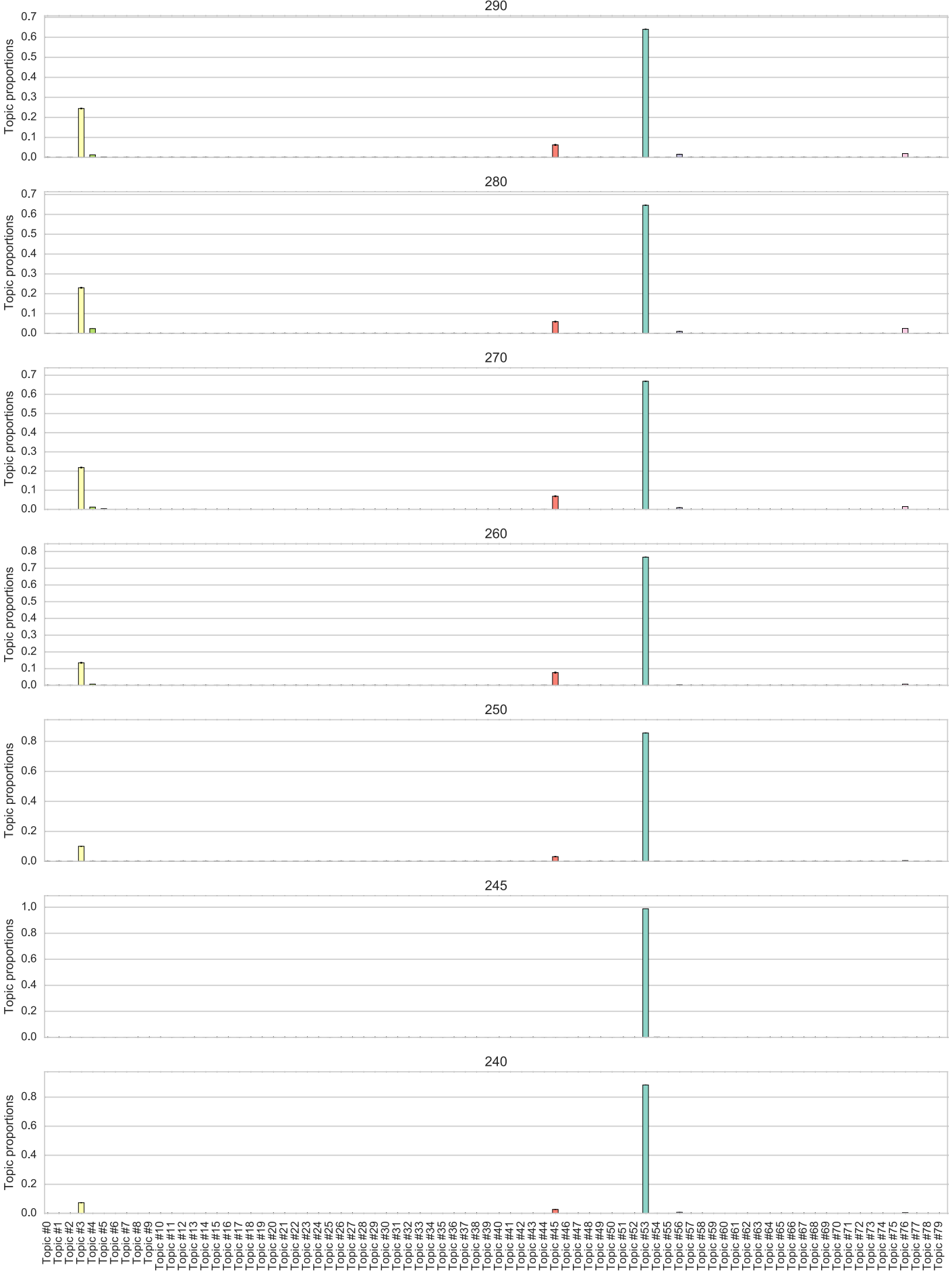

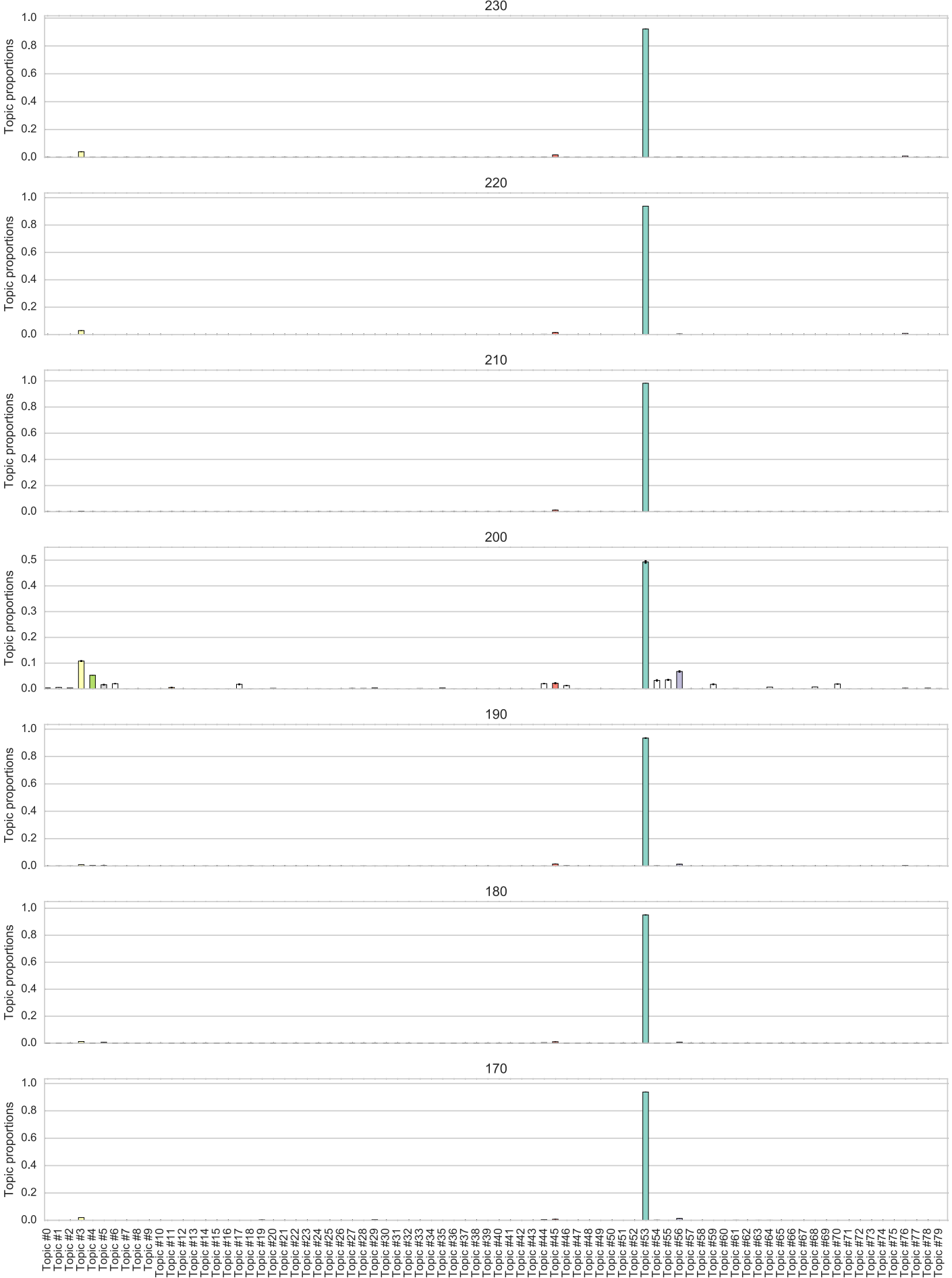

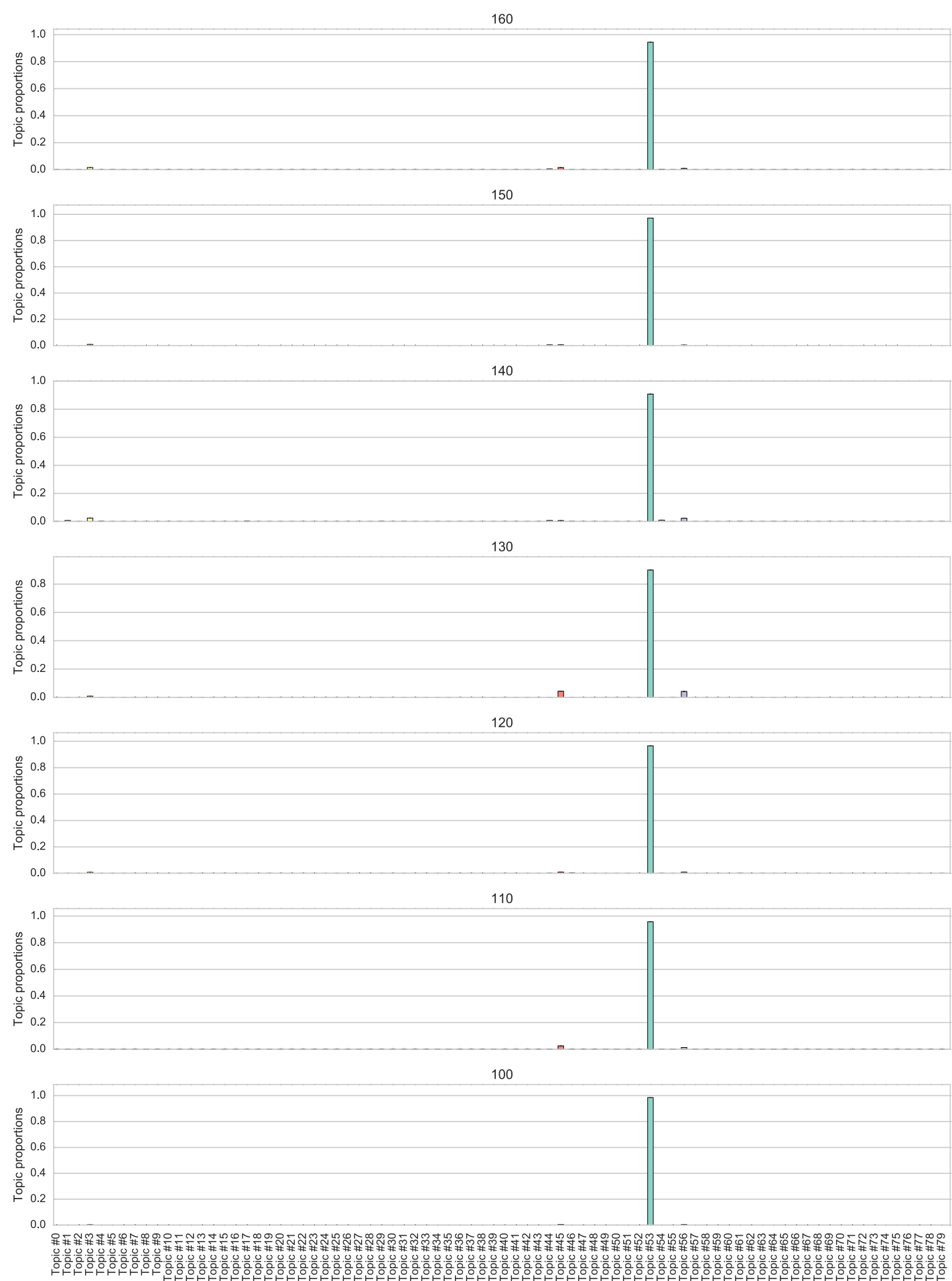

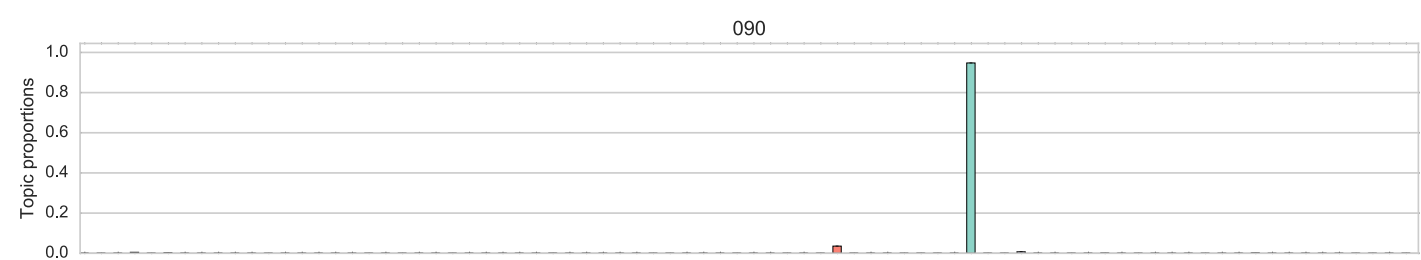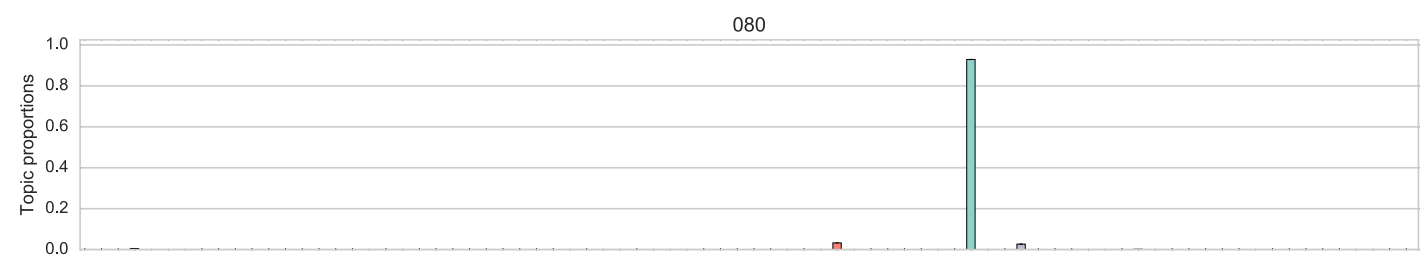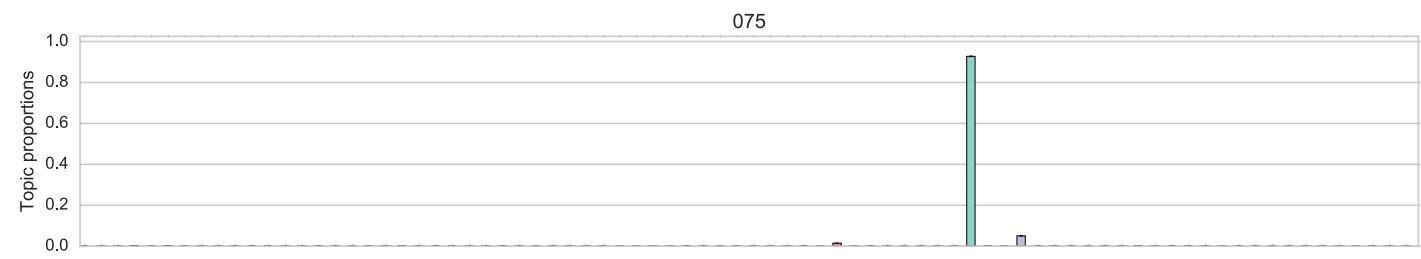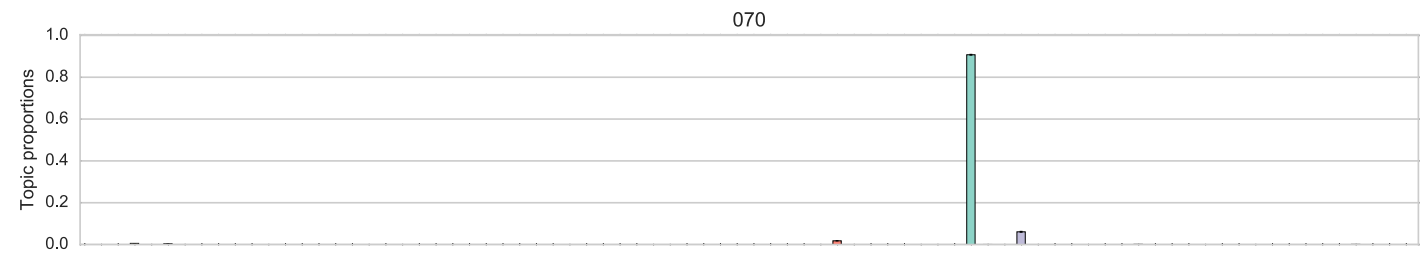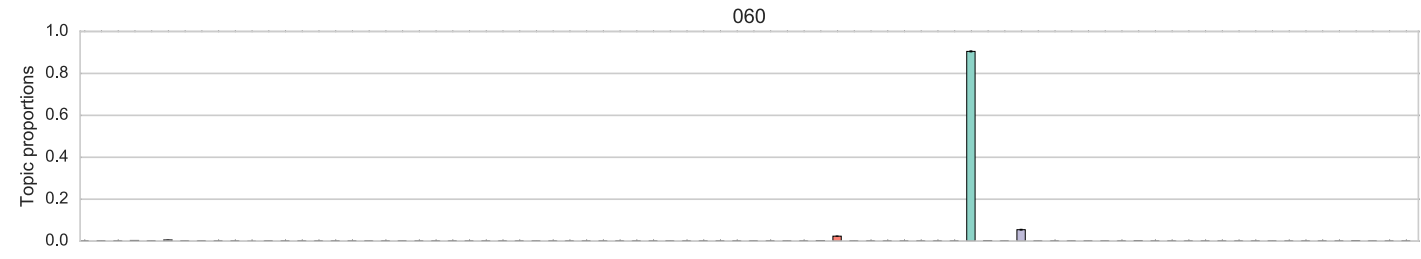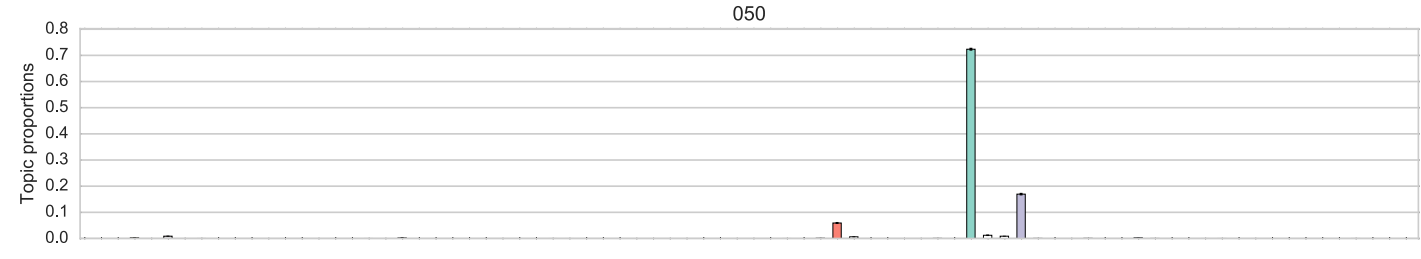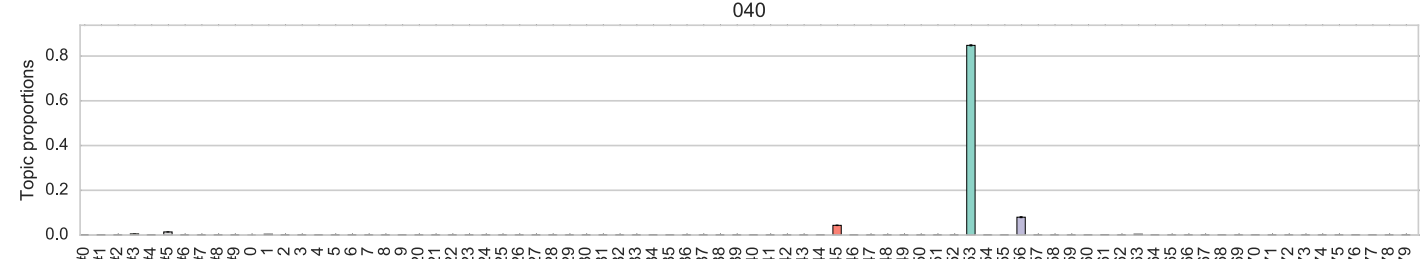

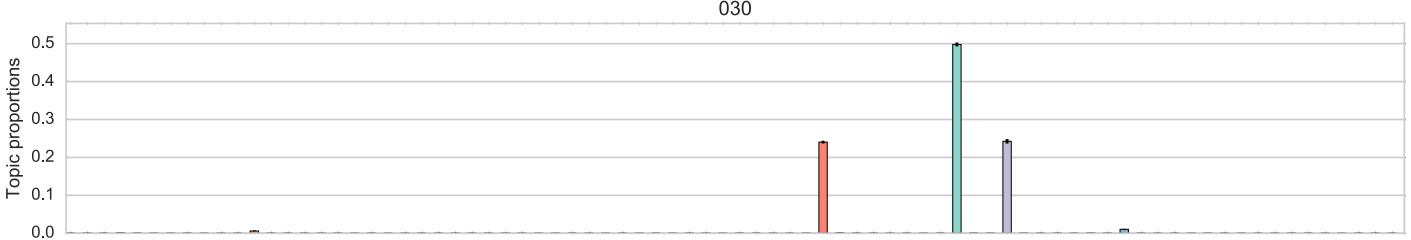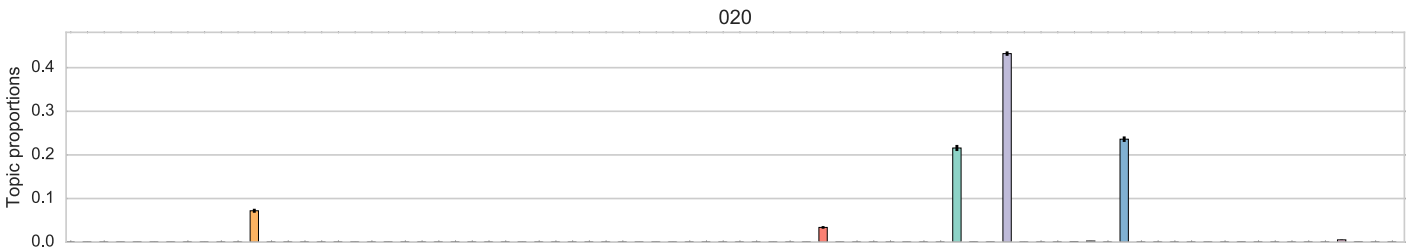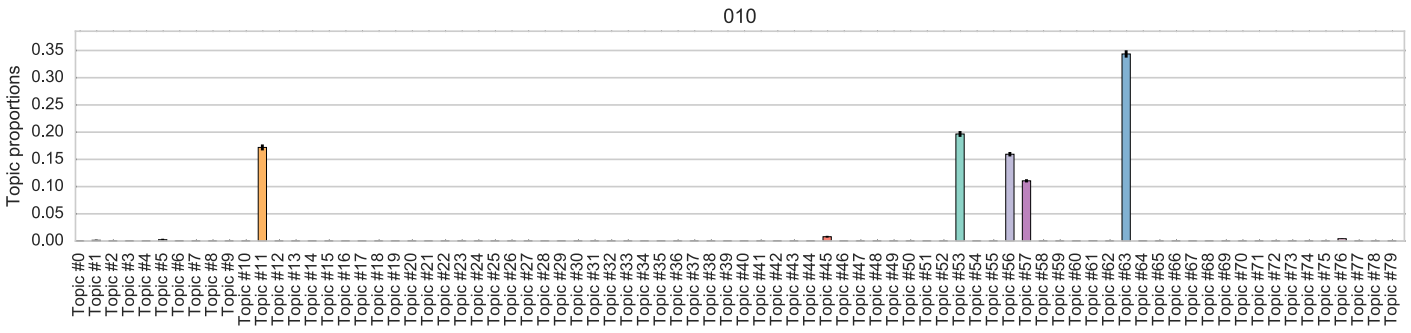

Supplement: S7 Fig — Topic proportions were calculated by drawing 100 samples from 100 independent Gibbs sampling chains with different initial settings. Bars represent the average values and the error bars represent standard deviations of 100 samples. Topic proportions were stable among different chains at any point on the Tamagawa river. (PDF) [file pcbi.1006143.s007.pdf]

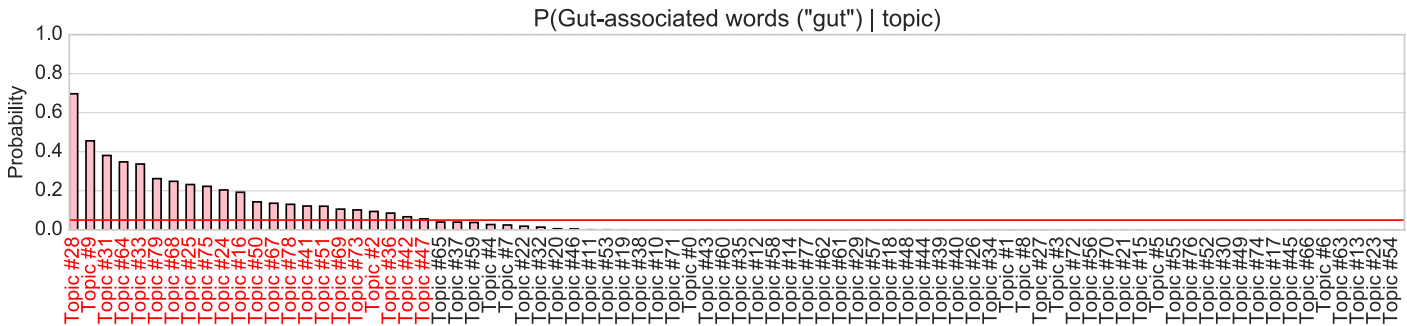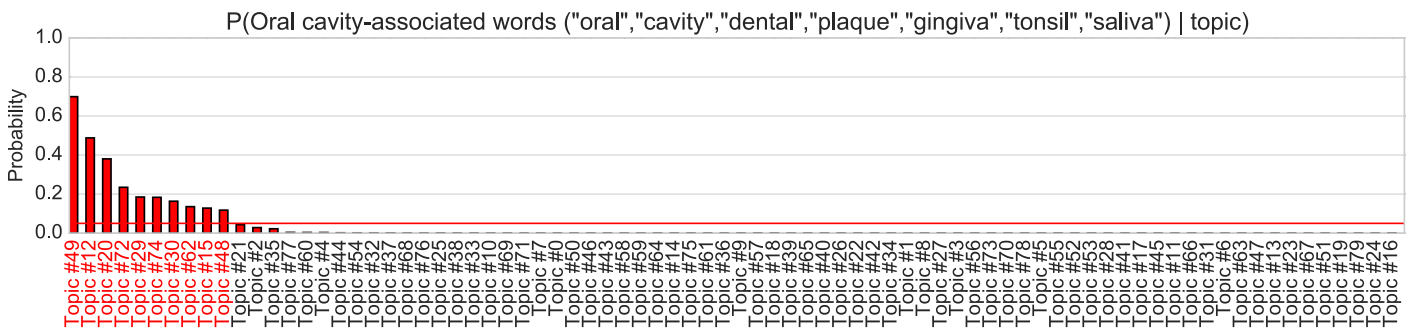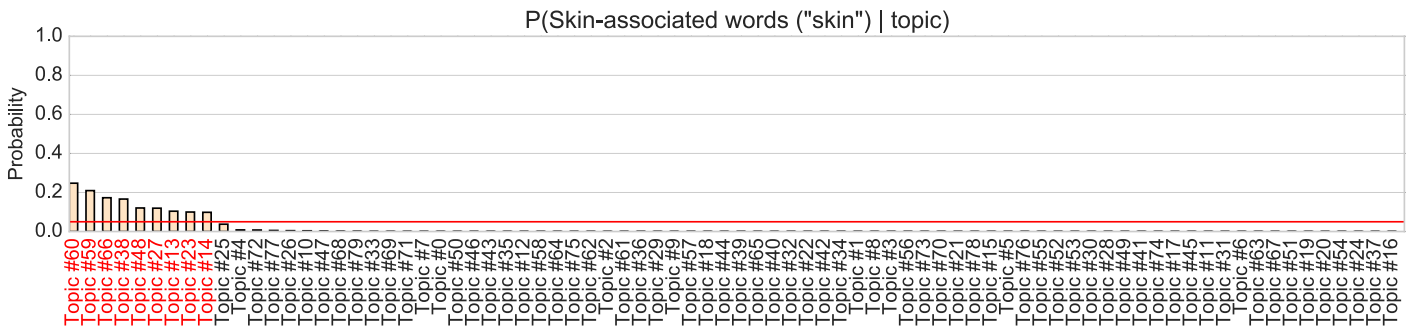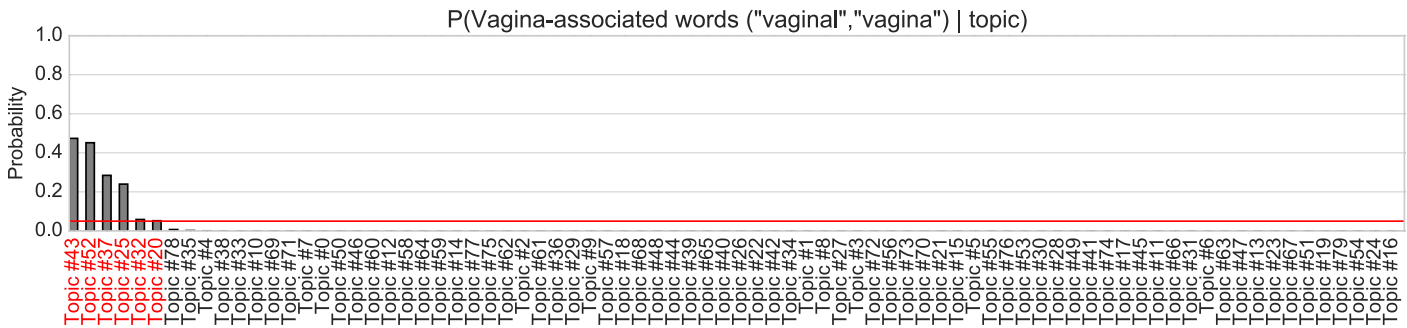

Supplement: S8 Fig — Each bar represents a probability of the environment associated words of each topic. Topics that can generate a specific word with above a probability of 5% (red line) was decided as environment-associated topics. (PDF) [file pcbi.1006143.s008.pdf]
