# Supplementary material for: Latent environment allocation of microbial community data
Source: PLoS Comput Biol. 2018 Jun 6;14(6):e1006143. doi: 10.1371/journal.pcbi.1006143 (PMC6005635; doi:10.1371/journal.pcbi.1006143)
Supplement: S2 Fig — For each of the topics, the picture used in the LEA global map, 20 words with the highest generation probabilities, and the pie chart for the genus generation probability are shown. In each pie chart, the genera with the 15 highest generation probabilities are color-coded by their phylum level taxonomy, and other less probable genera are in gray. (PDF) [file pcbi.1006143.s002.pdf]

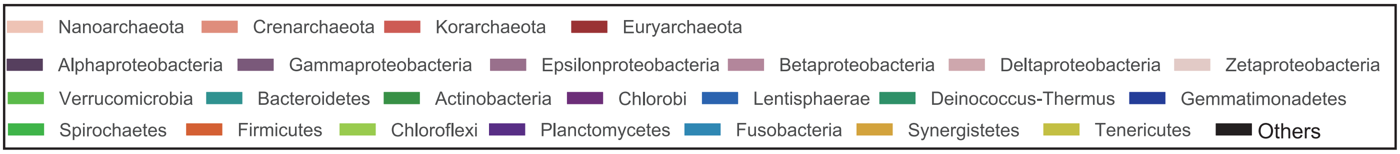

Topic #0

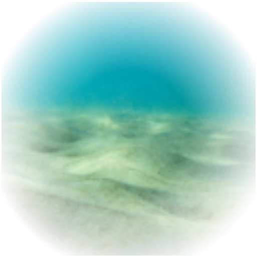

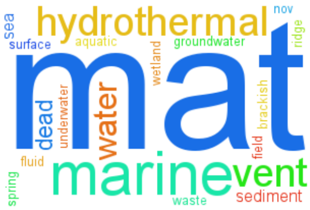

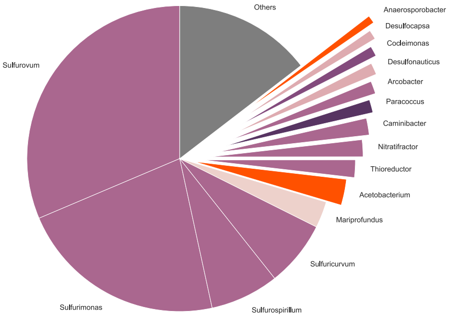

Image from:
https://pixabay.com/en/background-blue-floor-ocean-sand-17520/

Topic #1

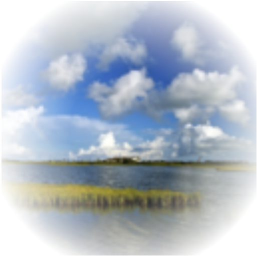

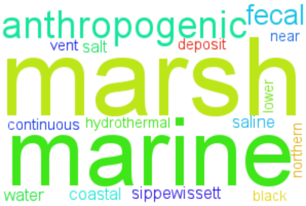

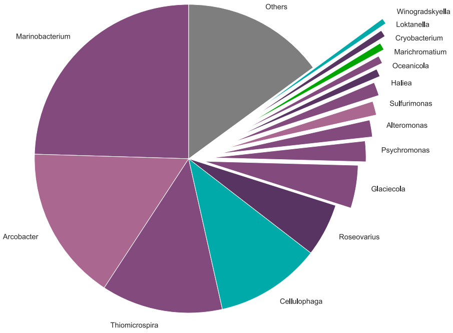

Image from:
https://pixabay.com/en/landscape-scenic-water-outdoors-1593808/

Topic #2

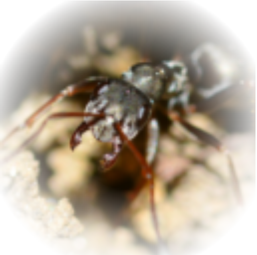

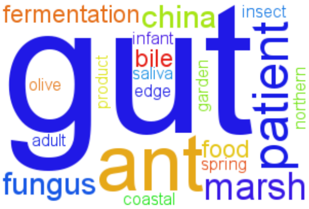

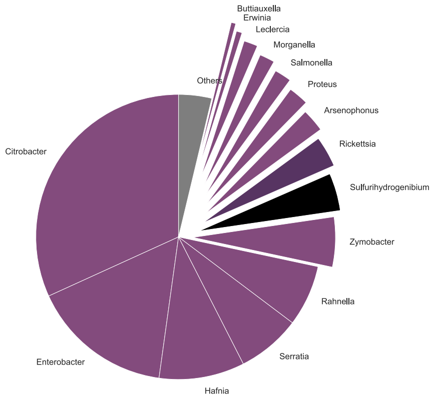

Image from:
https://pixabay.com/en/insects-serviformica-cunicularia-827787/

Topic #3

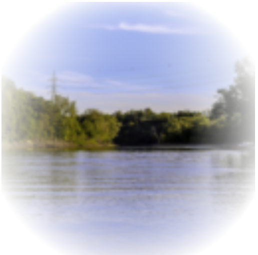

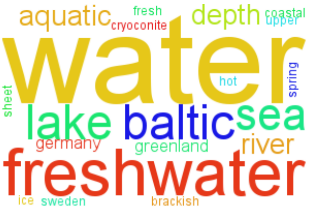

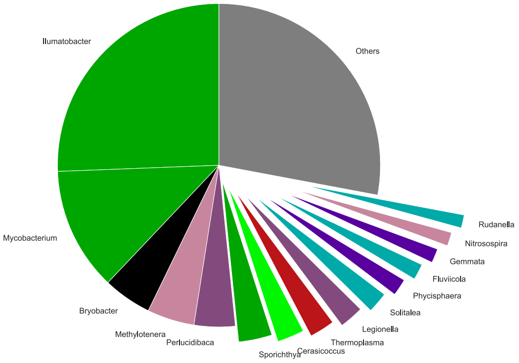

Image from:
https://pixabay.com/en/mississippi-river-landscape-forest-958586/

Topic #4

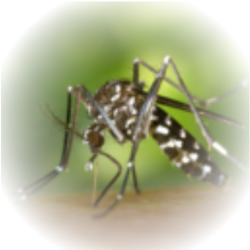

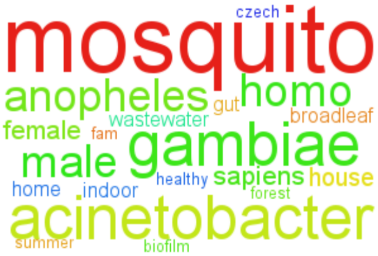

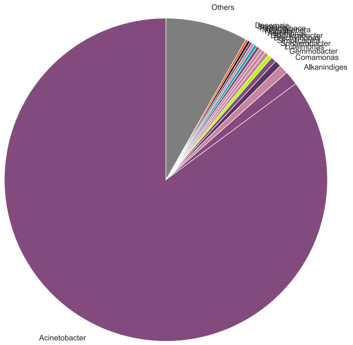

Image from:
https://pixabay.com/en/tiger-mosquito-mosquito-49141/

Topic #5

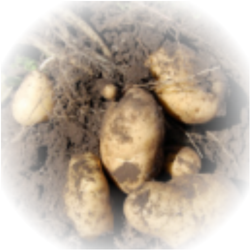

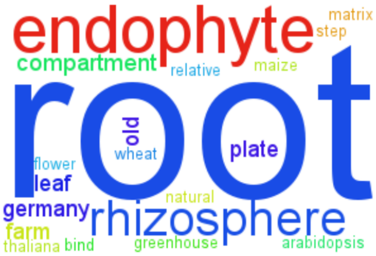

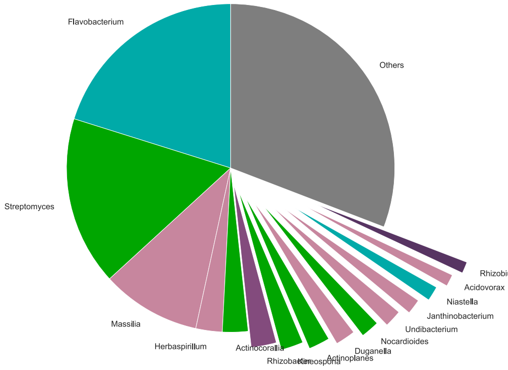

Image from:
https://pixabay.com/en/potatoes-land-fruit-why-potato-1637280/

Topic #6

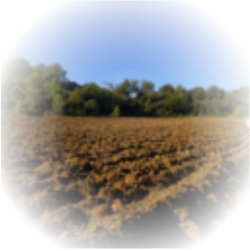

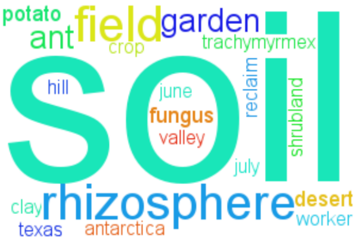

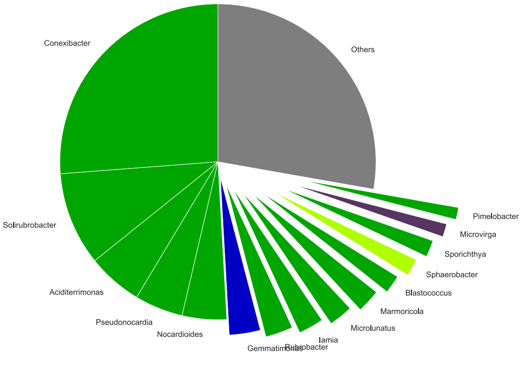

Image from:
https://pixabay.com/en/tractor-labour-agricultural-machine-1732125/

Topic #7

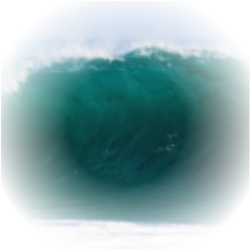

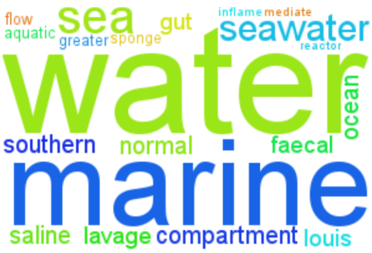

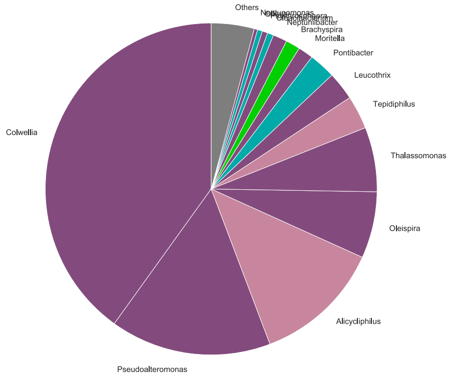

Image from:
https://pixabay.com/en/ocean-wave-sea-water-tide-tidal-918999/

Topic #8

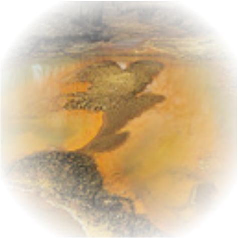

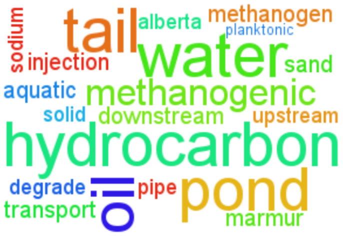

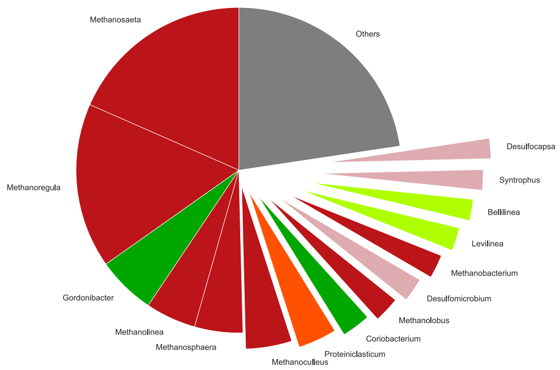

Image from:
https://commons.wikimedia.org/wiki/File%3AIron_hydroxide_precipitate_in_stream.jpg

Topic #9

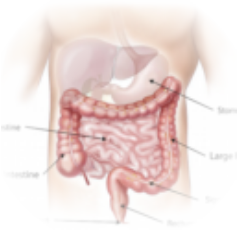

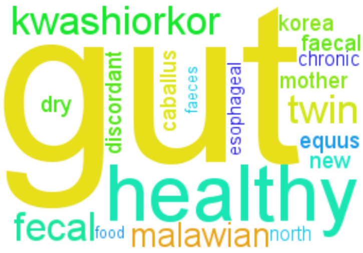

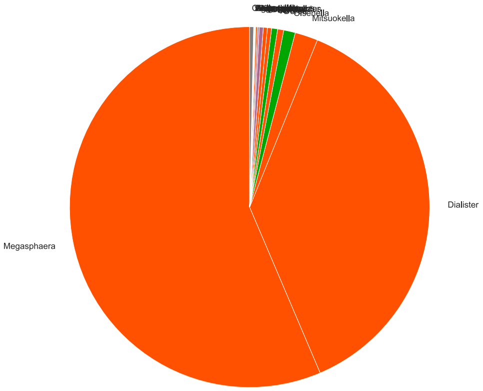

Image from:
https://pixabay.com/en/abdomen-intestine-large-small-1698565/

Topic #10

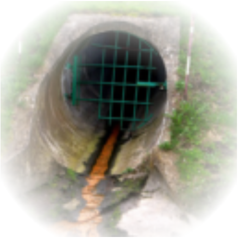

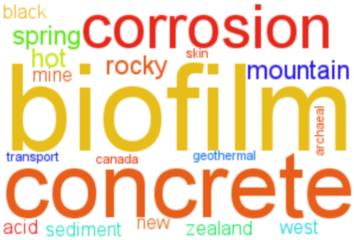

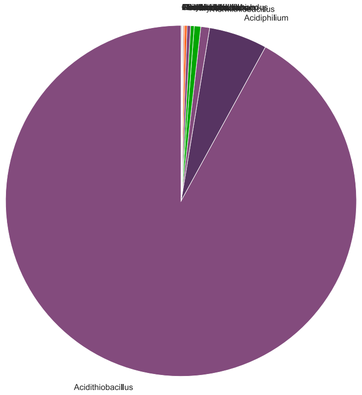

Image from:
https://pixabay.com/en/channel-sewage-sludge-unsanitary-1692671/

Topic #11

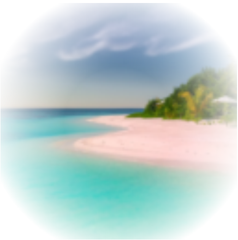

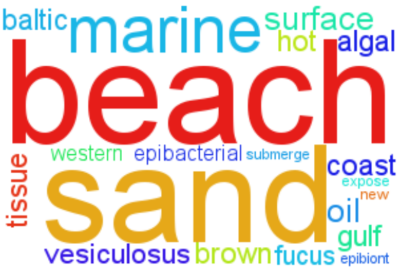

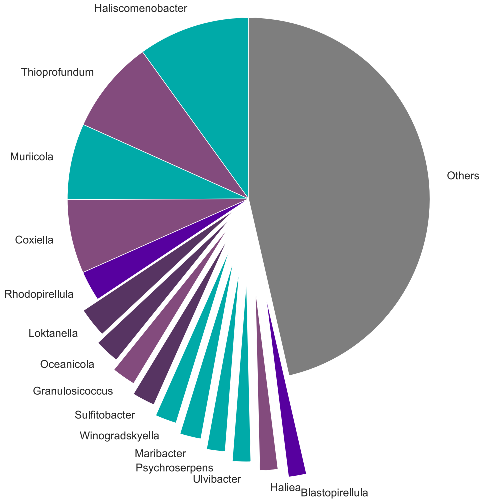

Image from:
https://pixabay.com/en/pink-beach-beach-paradise-1761410/

Topic #12

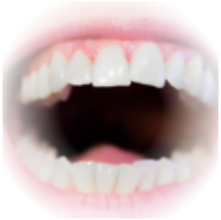

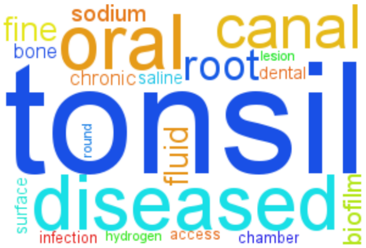

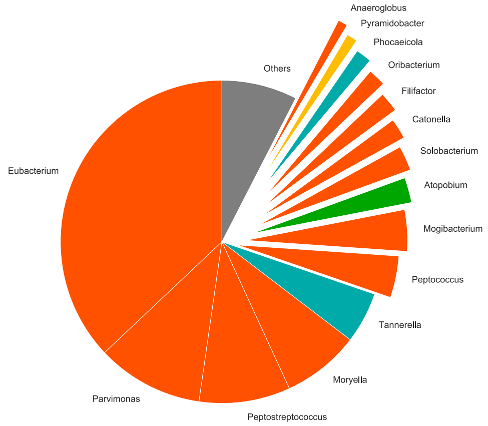

Image from:
https://pixabay.com/en/teeth-dentist-dental-mouth-white-1652937/

Topic #13

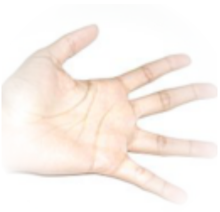

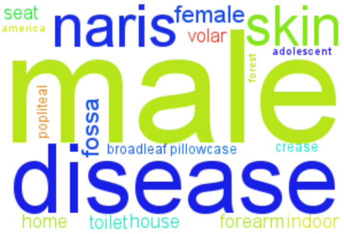

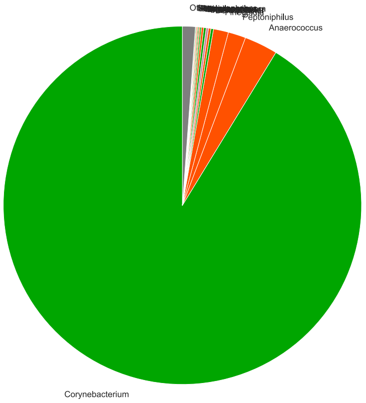

Image from:
https://commons.wikimedia.org/wiki/File%3AHuman_Hand.JPG

Topic #14

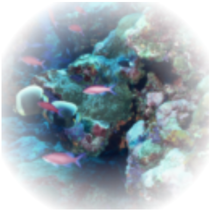

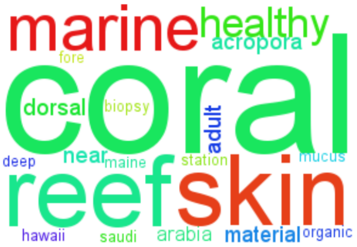

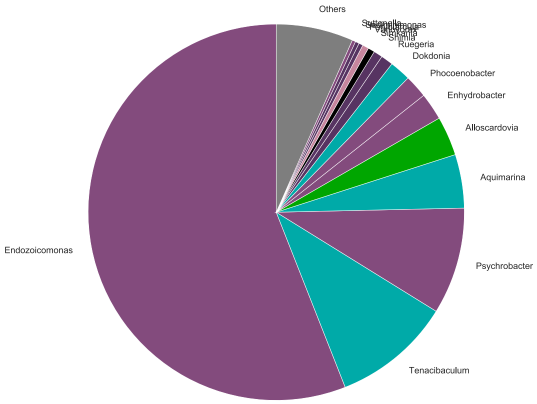

Image from:
https://pixabay.com/en/marine-harbour-underwater-world-1629758/

Topic #15

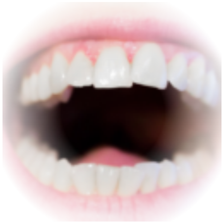

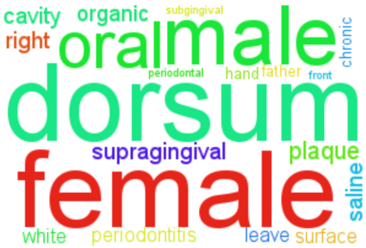

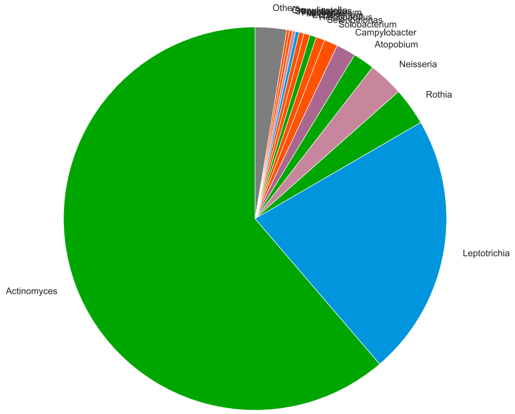

Image from:
https://pixabay.com/en/teeth-dentist-dental-mouth-white-1652937/

Topic #16

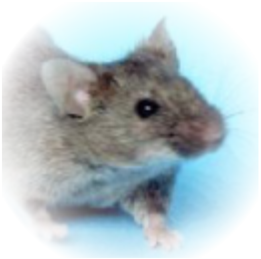

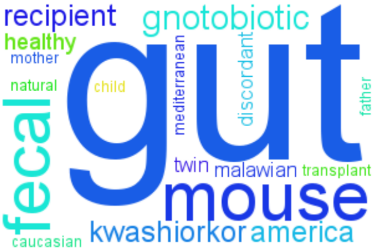

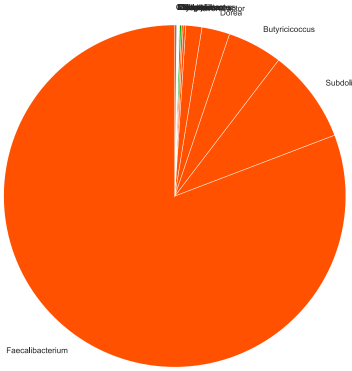

Image from:
https://pixabay.com/en/teeth-dentist-dental-mouth-white-1652937/

Topic #17

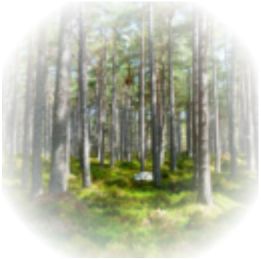

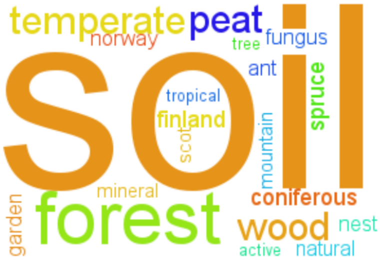

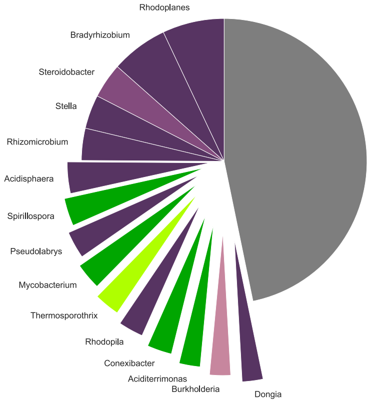

Image from:
https://pixabay.com/en/forest-trees-ecology-environment-272595/

Topic #18

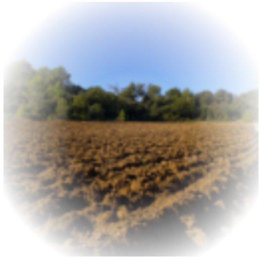

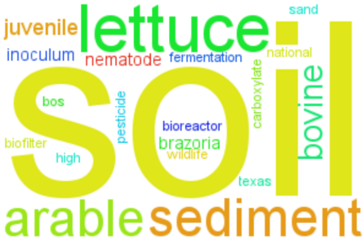

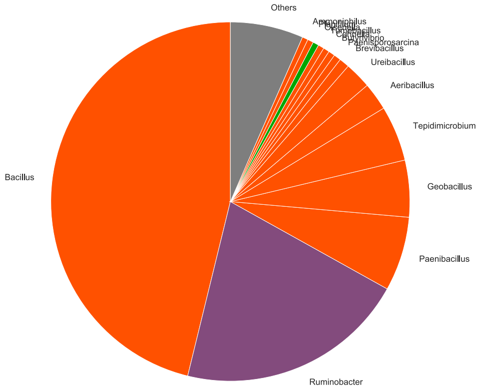

Image from:
https://pixabay.com/en/tractor-labour-agricultural-machine-1732125/

Topic #19

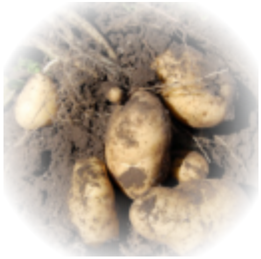

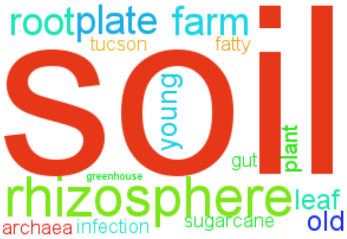

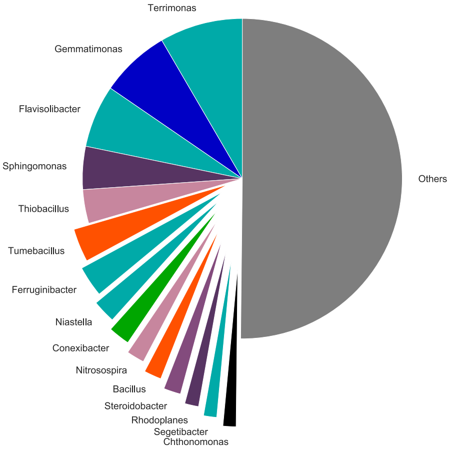

Image from:
https://pixabay.com/en/potatoes-land-fruit-why-potato-1637280/

Topic #20

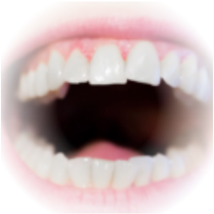

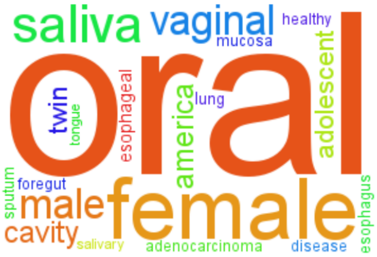

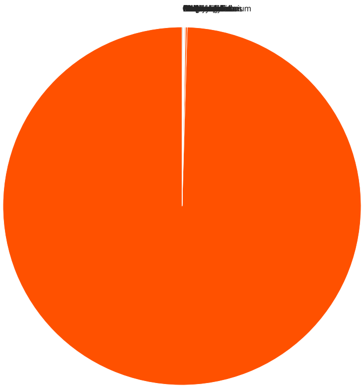

Image from:
https://pixabay.com/en/teeth-dentist-dental-mouth-white-1652937/

Topic #21

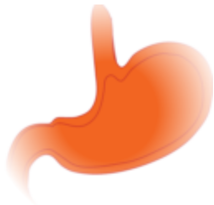

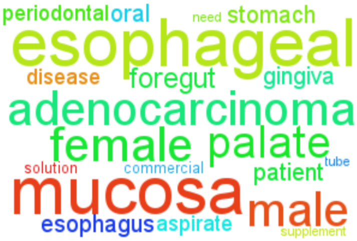

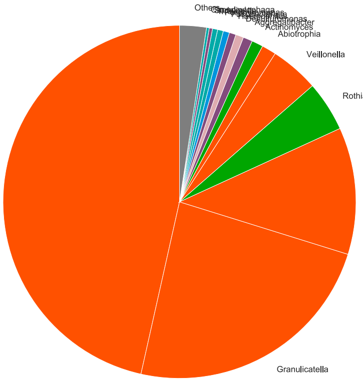

Image from:
https://pixabay.com/en/stomach-anatomy-human-body-biology-310730/

Topic #22

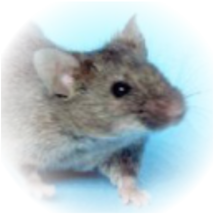

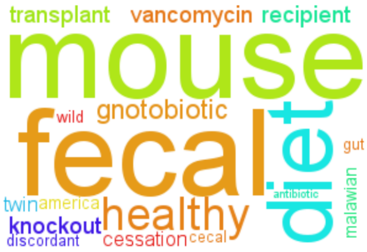

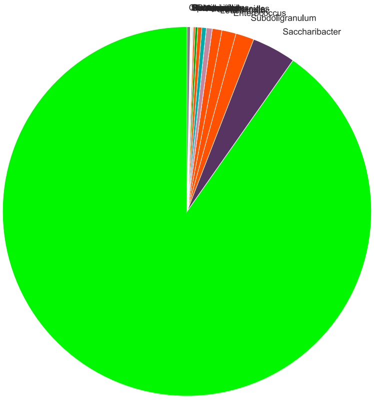

Image from:
https://commons.wikimedia.org/wiki/File%3AHouse_mouse.jpg

Topic #23

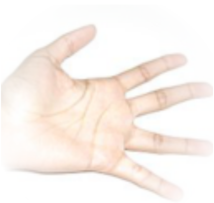

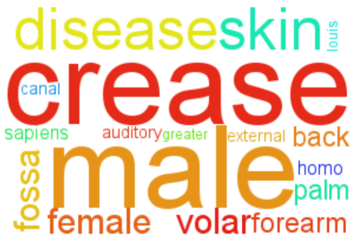

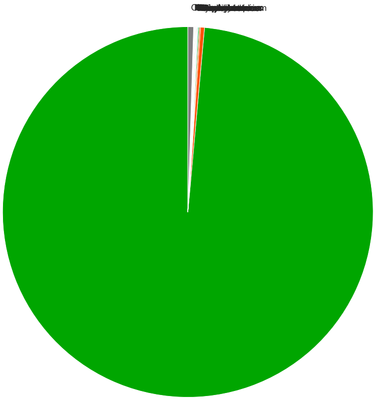

Image from:
https://commons.wikimedia.org/wiki/File%3AHuman_Hand.JPG

Topic #24

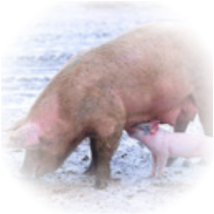

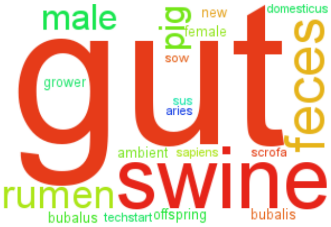

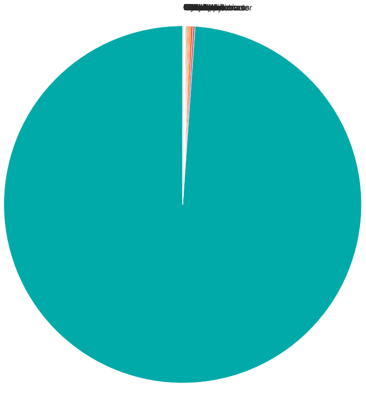

Image from:
https://commons.wikimedia.org/wiki/File%3ASow_with_piglet.jpg

Topic #25

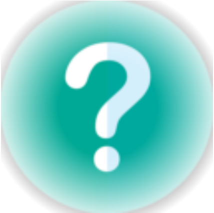

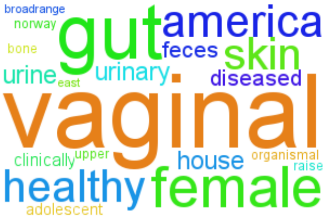

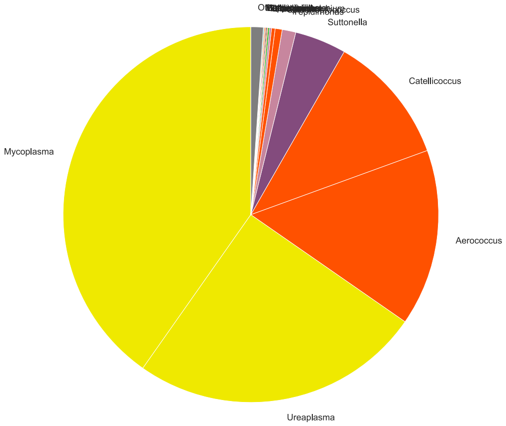

Image from:
https://pixabay.com/en/help-information-question-tip-1724292/

Topic #26

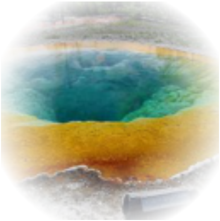

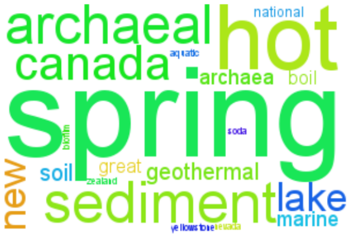

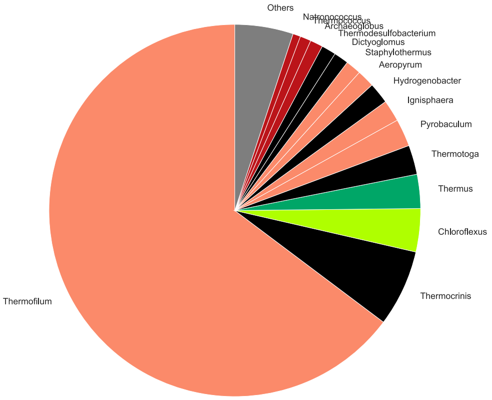

Image from:
https://commons.wikimedia.org/wiki/File%3AThermal_hot_spring.jpg

Topic #27

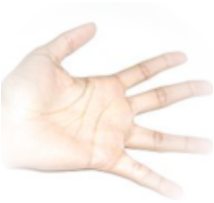

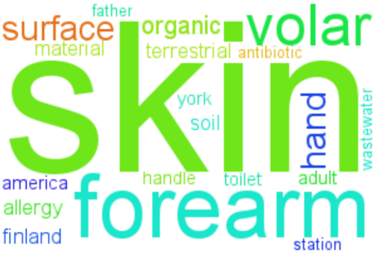

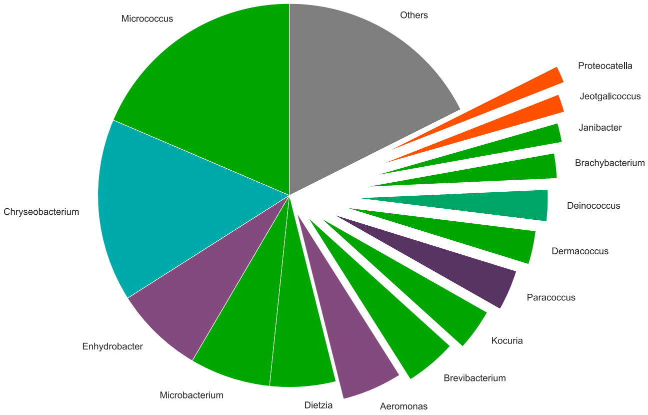

Image from:
https://commons.wikimedia.org/wiki/File%3AHuman_Hand.JPG

Topic #28

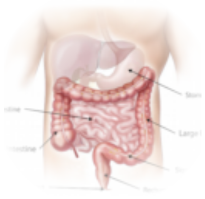

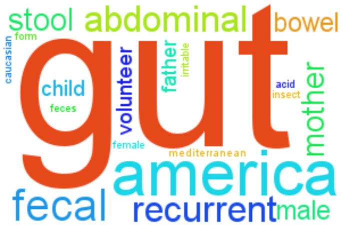

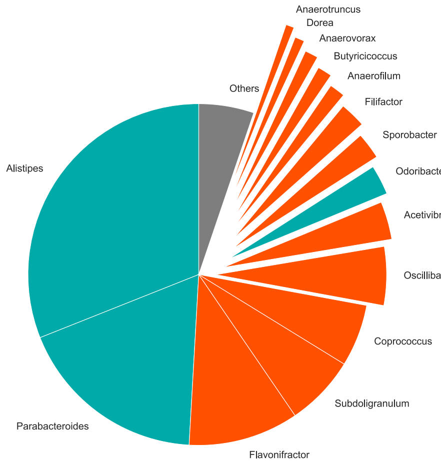

Image from:
https://pixabay.com/en/abdomen-intestine-large-small-1698565/

Topic #29

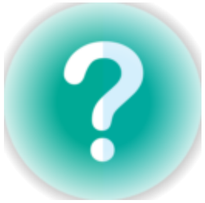

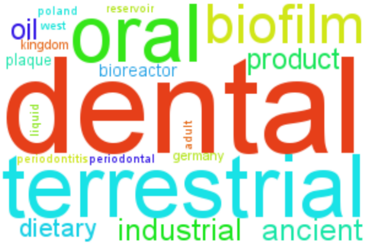

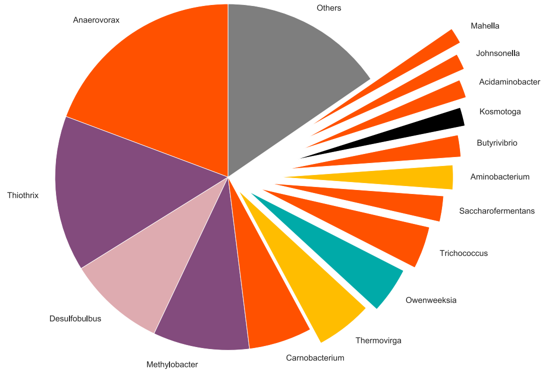

Image from:
https://pixabay.com/en/help-information-question-tip-1724292/

Topic #30

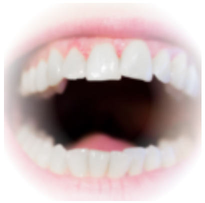

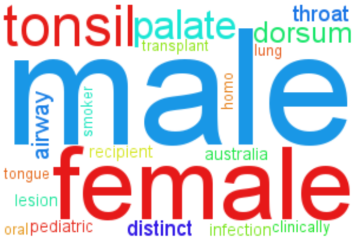

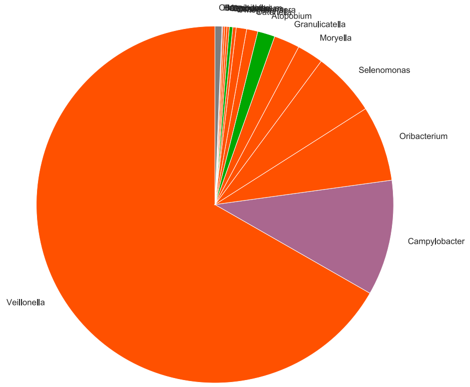

Image from:
https://pixabay.com/en/teeth-dentist-dental-mouth-white-1652937/

Topic #31

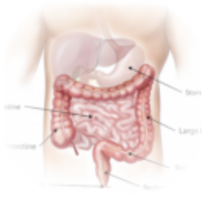

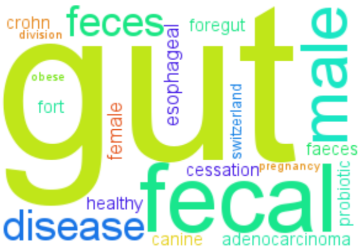

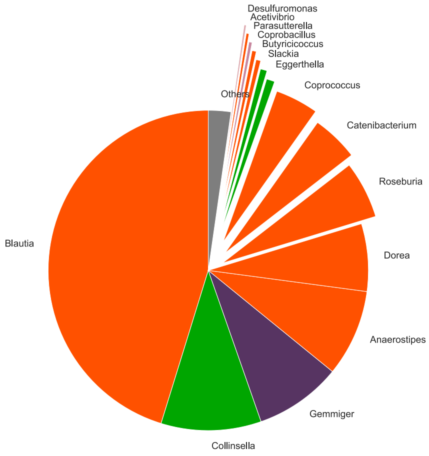

Image from:
https://pixabay.com/en/abdomen-intestine-large-small-1698565/

Topic #32

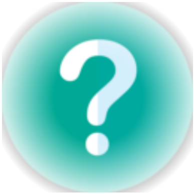

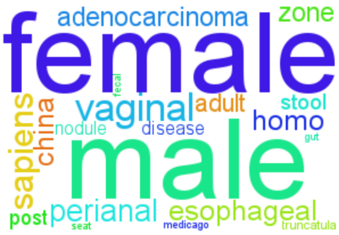

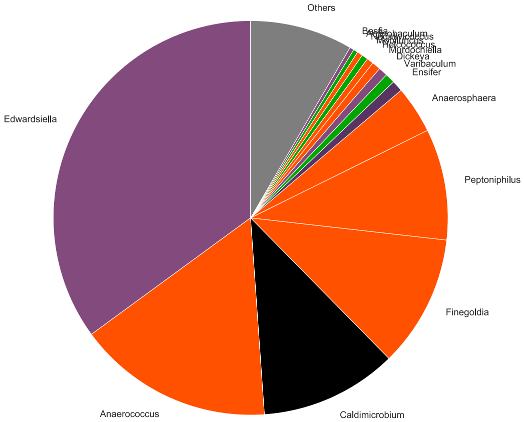

Image from:
https://pixabay.com/en/help-information-question-tip-1724292/

Topic #33

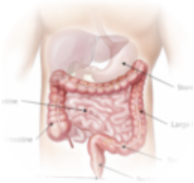

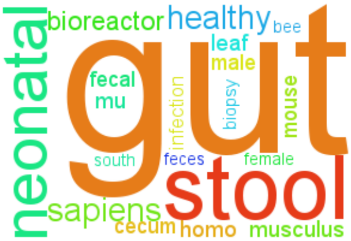

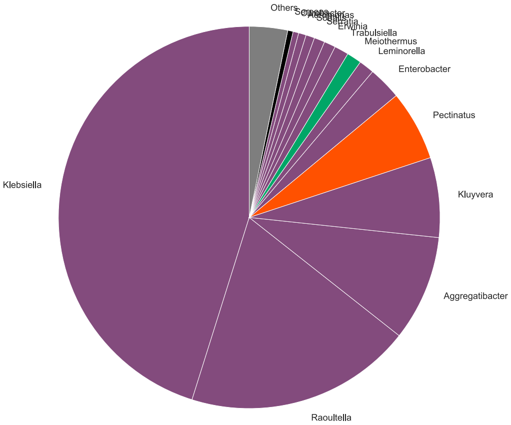

Image from:
https://pixabay.com/en/abdomen-intestine-large-small-1698565/

Topic #34

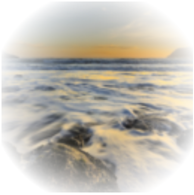

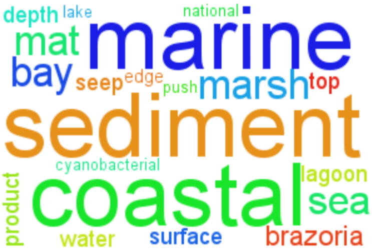

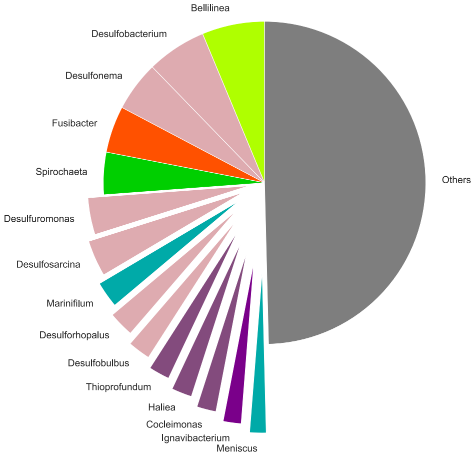

Image from:
https://pixabay.com/en/sunrise-ocean-sea-coast-1239728/

Topic #35

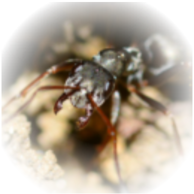

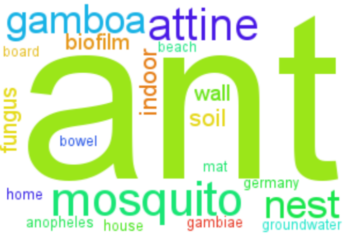

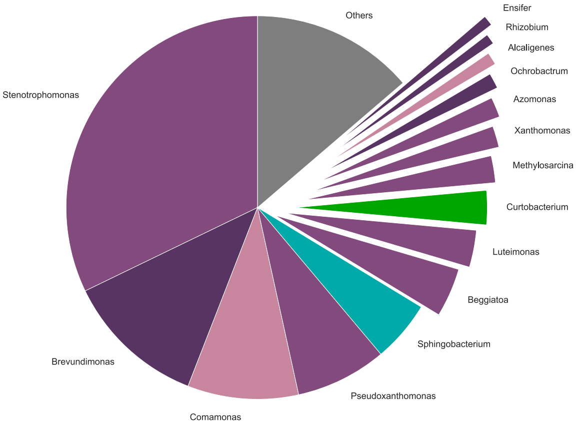

Image from:
https://pixabay.com/en/insects-serviformica-cunicularia-827787/

Topic #36

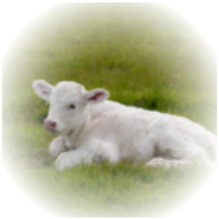

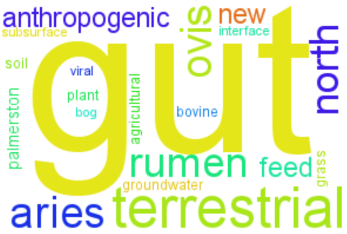

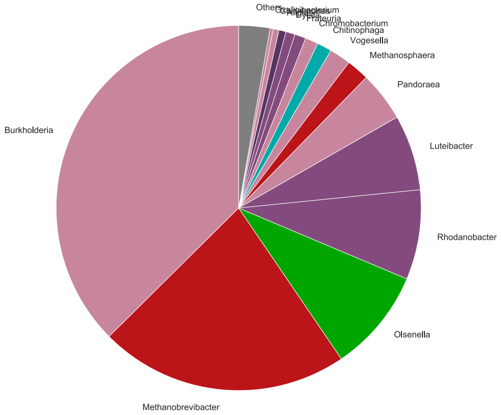

Image from:
https://pixabay.com/en/calf-field-farm-rural-agriculture-1726620/

Topic #37

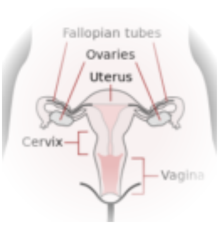

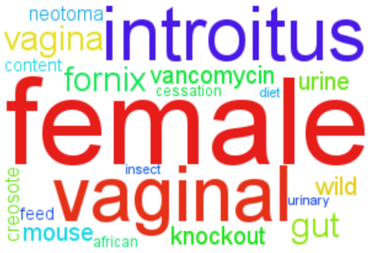

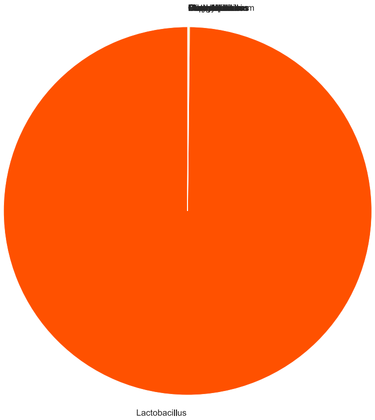

Image from:
https://commons.wikimedia.org/wiki/File%3AScheme_female_reproductive_system-en.svg

Topic #38

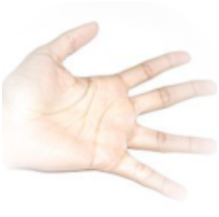

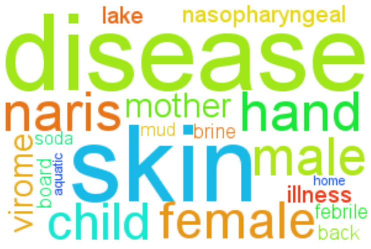

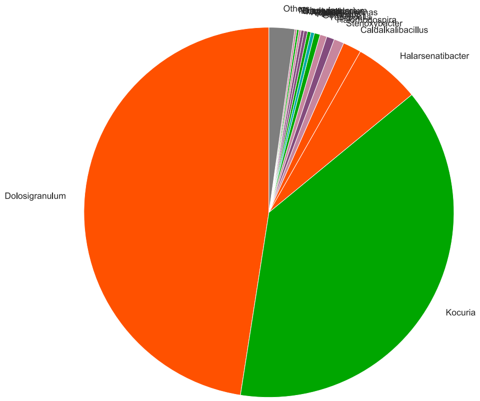

Image from:
https://commons.wikimedia.org/wiki/File%3AHuman_Hand.JPG

Topic #39

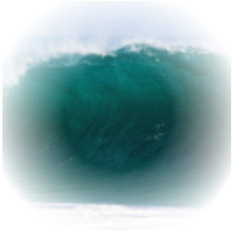

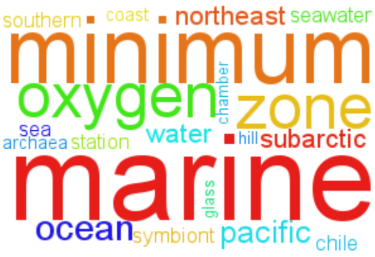

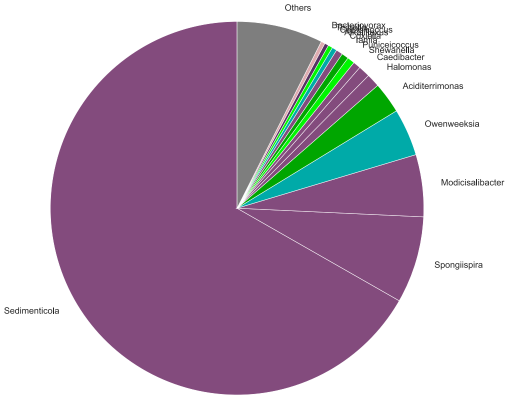

Image from:
https://pixabay.com/en/ocean-wave-sea-water-tide-tidal-918999/
