# Supplementary material for: Latent environment allocation of microbial community data
Source: PLoS Comput Biol. 2018 Jun 6;14(6):e1006143. doi: 10.1371/journal.pcbi.1006143 (PMC6005635; doi:10.1371/journal.pcbi.1006143)
Supplement: S3 Fig — (PDF) [file pcbi.1006143.s003.pdf]

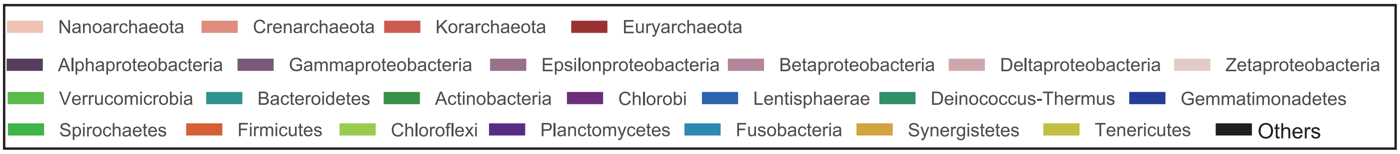

Topic #40

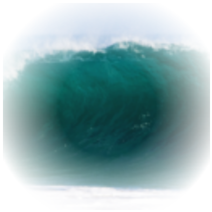

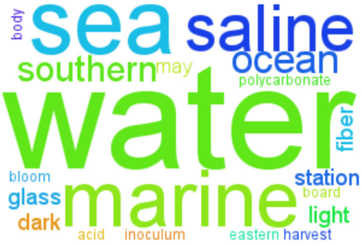

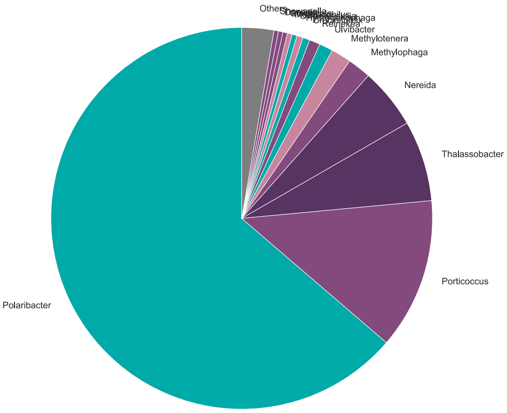

Image from:
https://pixabay.com/en/calf-field-farm-rural-agriculture-1726620/

Topic #41

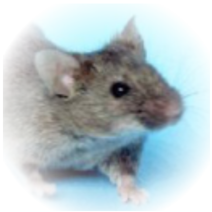

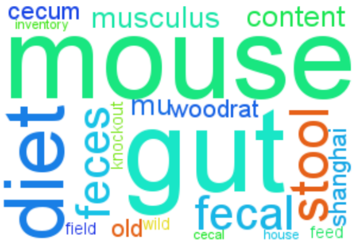

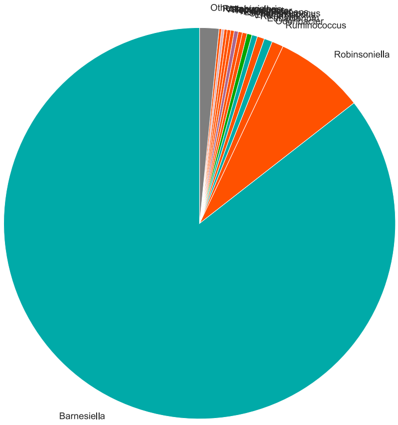

Image from:
https://commons.wikimedia.org/wiki/File%3AHouse_mouse.jpg

Topic #42

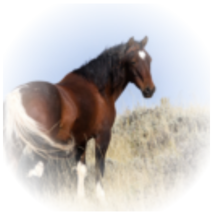

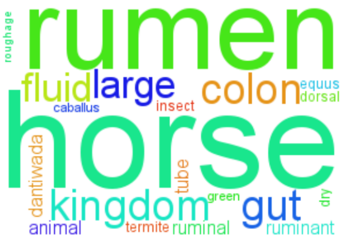

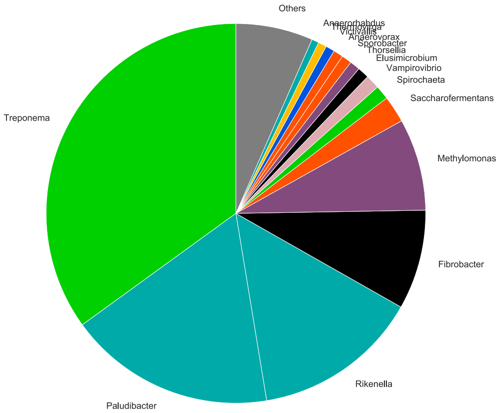

Image from:
https://pixabay.com/en/wild-horses-wild-mustangs-mustangs-1760581/

Topic #43

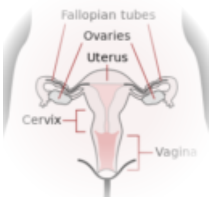

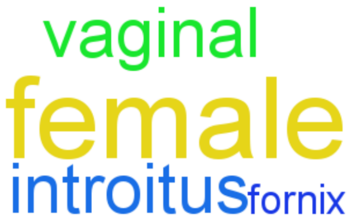

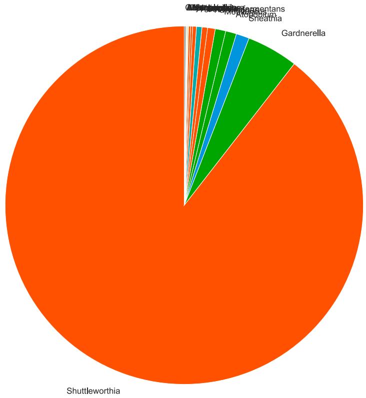

Image from:
https://commons.wikimedia.org/wiki/File%3AScheme_female_reproductive_system-en.svg

Topic #44

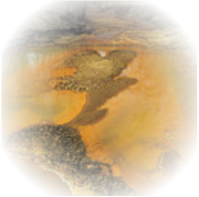

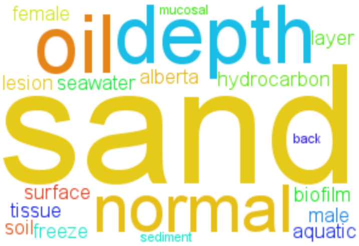

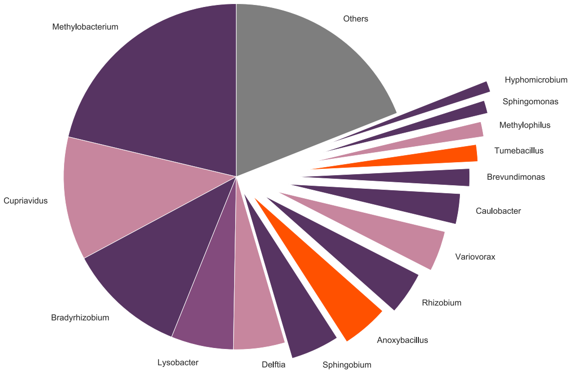

Image from:
https://commons.wikimedia.org/wiki/File%3AIron_hydroxide_precipitate_in_stream.

Topic #45

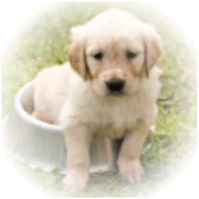

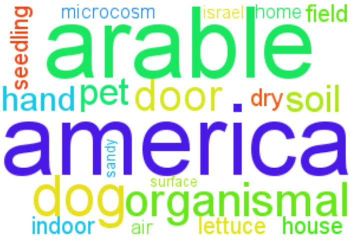

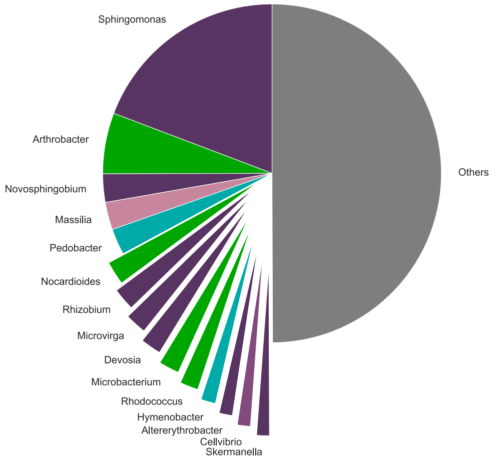

Image from:
https://pixabay.com/en/puppy-golden-retriever-dog-1207816/

Topic #46

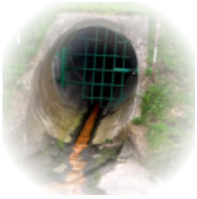

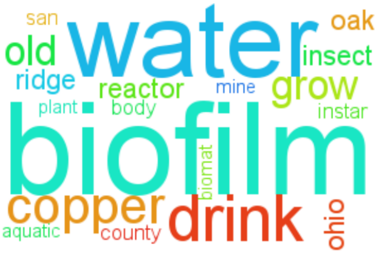

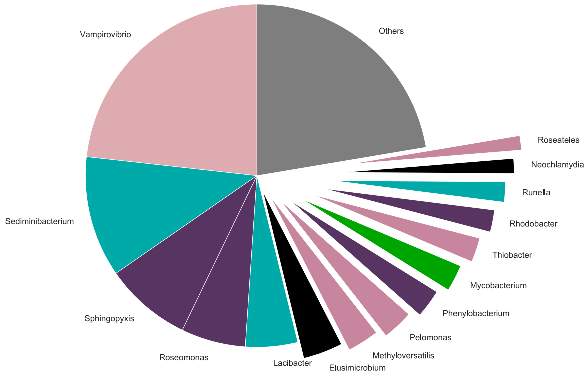

Image from:
https://pixabay.com/en/channel-sewage-sludge-unsanitary-1692671/

Topic #47

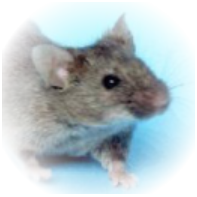

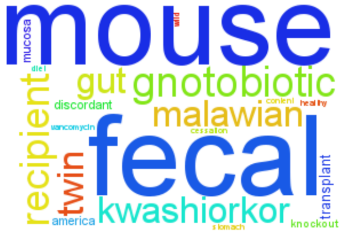

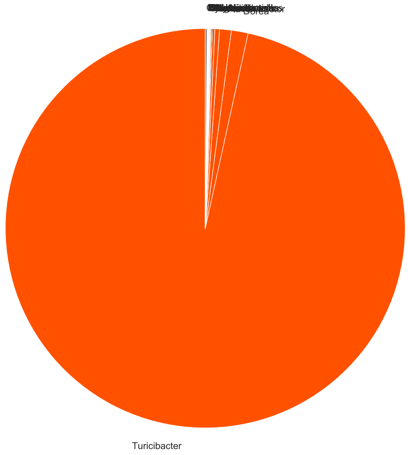

Image from:
https://commons.wikimedia.org/wiki/File%3AHouse_mouse.jpg

Topic #48

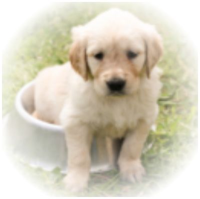

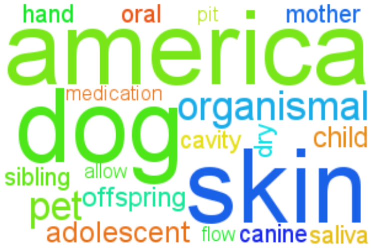

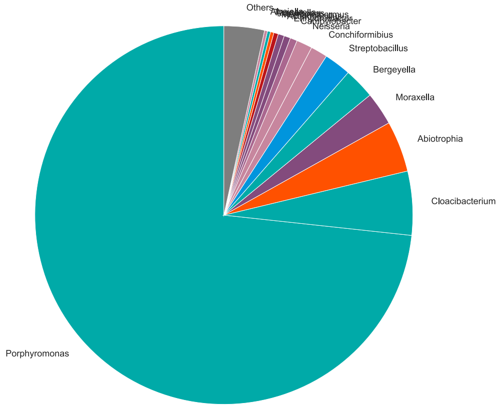

Image from:
https://pixabay.com/en/puppy-golden-retriever-dog-1207816/

Topic #49

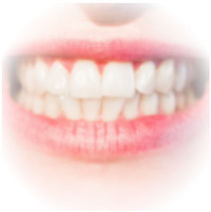

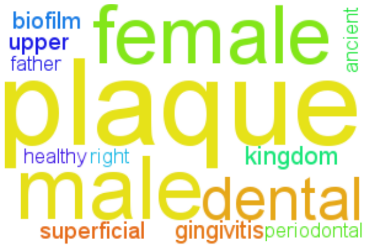

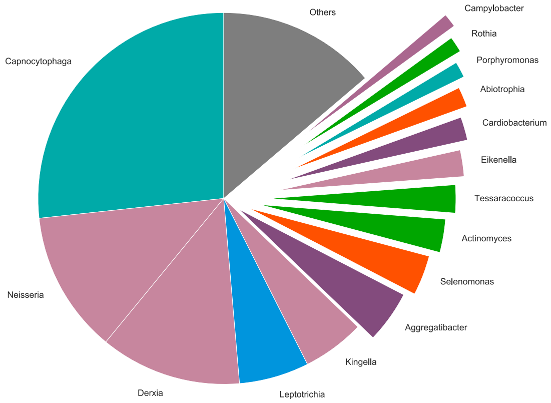

Image from:
https://pixabay.com/en/teeth-dentist-dental-mouth-tooth-1652976/

Topic #50

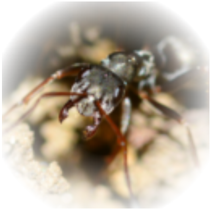

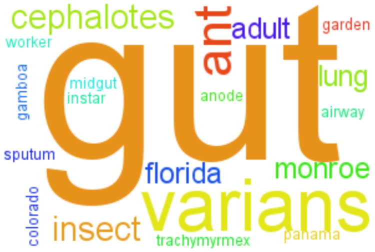

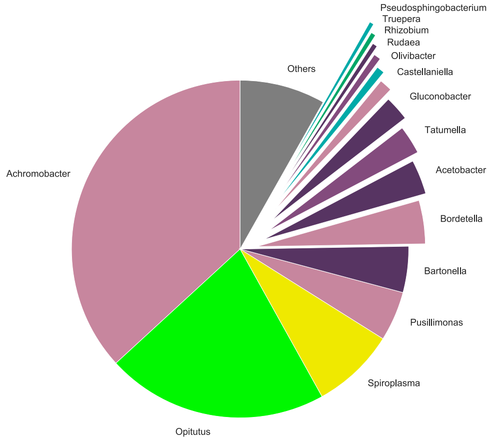

Image from:
https://pixabay.com/en/insects-serviformica-cunicularia-827787/

Topic #51

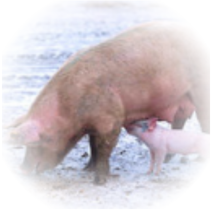

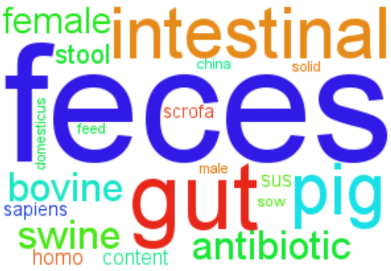

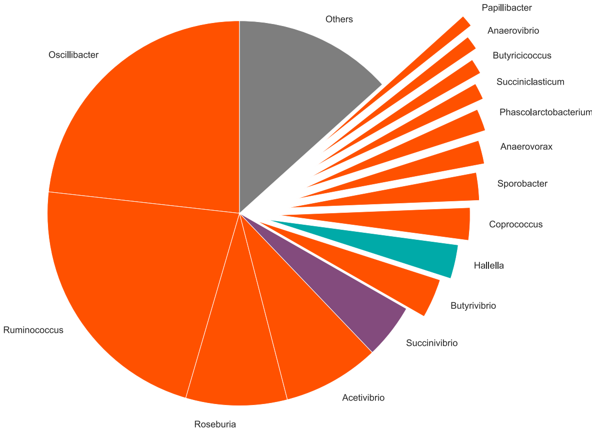

Image from:
https://commons.wikimed[illegible]

Topic #52

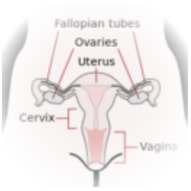

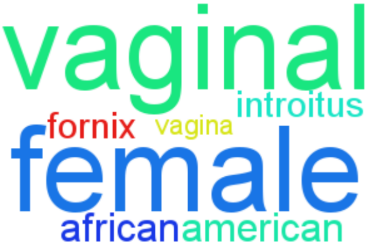

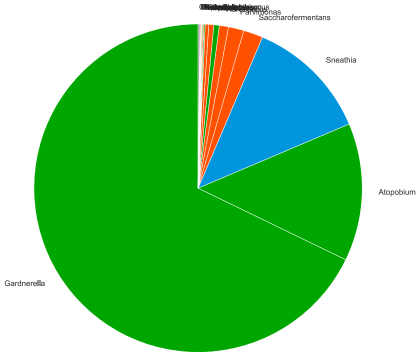

Image from:
https://commons.wikimedia.org/wiki/File%3AScheme_female_reproductive_system-en.svg

Topic #53

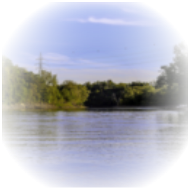

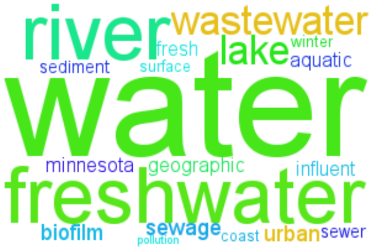

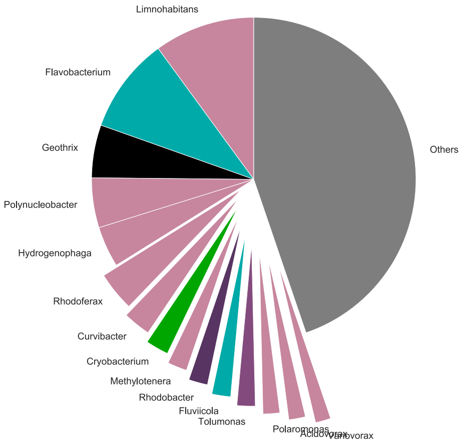

Image from:
https://pixabay.com/en/mississippi-river-landscape-forest-958586/

Topic #54

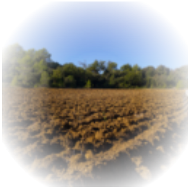

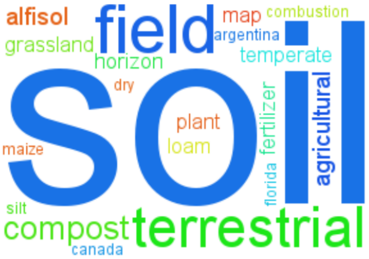

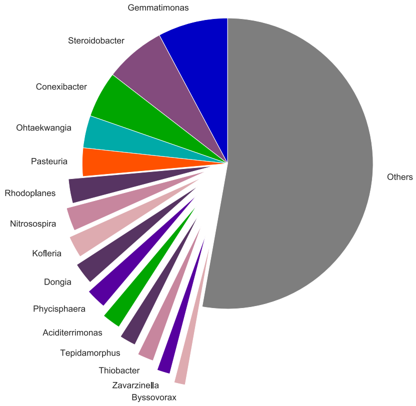

Image from:
https://pixabay.com/en/tractor-labour-agricultural-machine-1732125/

Topic #55

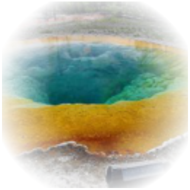

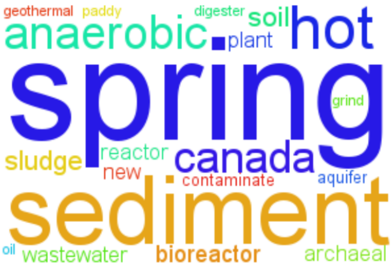

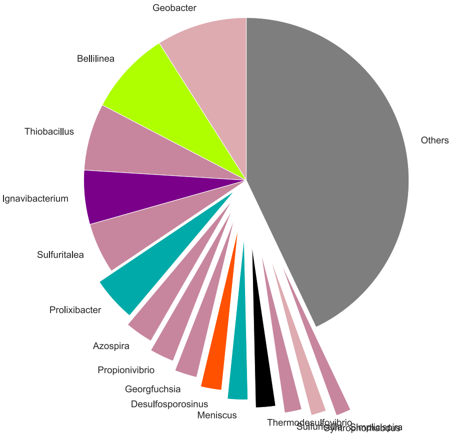

Image from:
https://commons.wikimedia.org/wiki/File%3AThermal_hot_spring.jpg

Topic #56

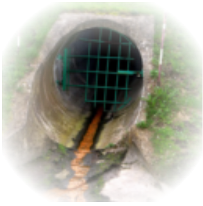

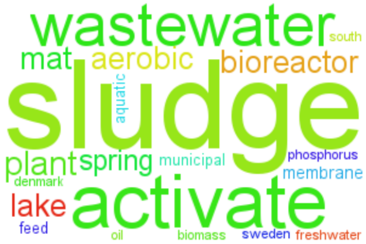

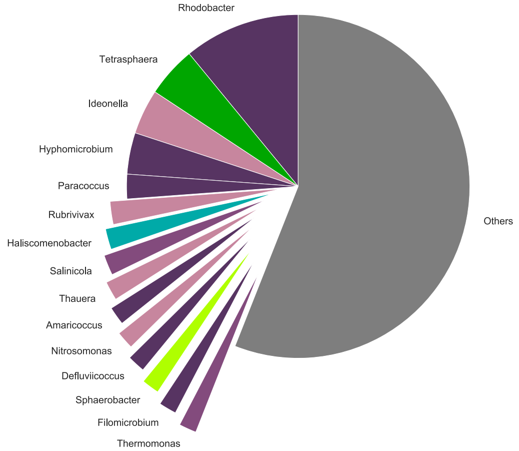

Image from:
https://pixabay.com/en/channel-sewage-sludge-unsanitary-1692671/

Topic #57

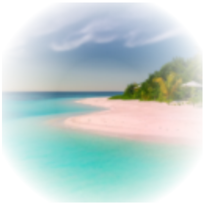

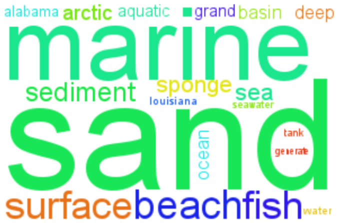

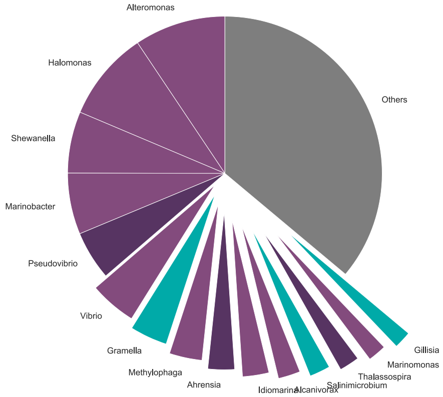

Image from:
https://pixabay.com/en/pink-beach-beach-paradise-1761410/

Topic #58

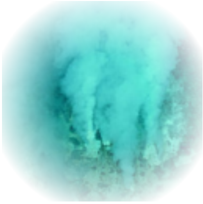

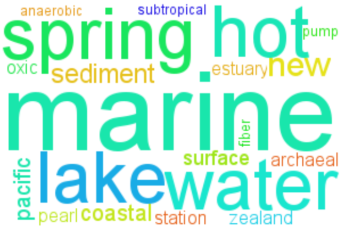

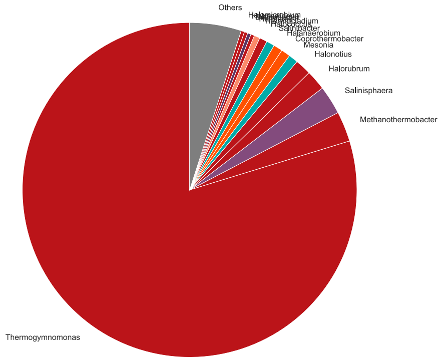

Image from:
https://commons.wikimedia.org/wiki/File%3AChampagne_vent_white_smokers.jpg

Topic #59

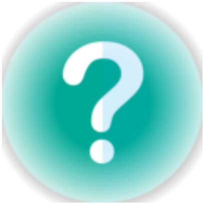

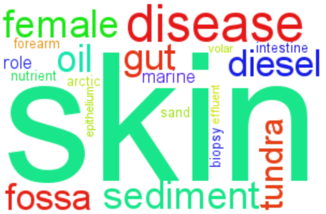

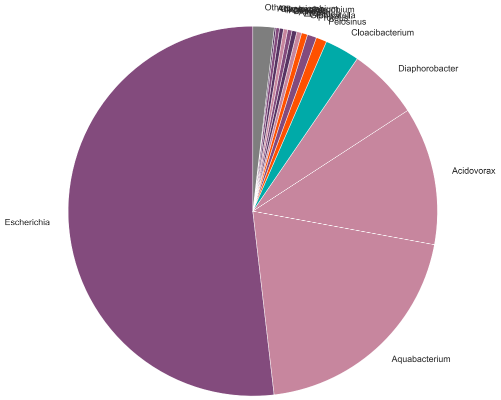

Image from:
https://commons.wikimedia.org/wiki/File%3AChampagne_vent_white_smokers.jpg

Topic #60

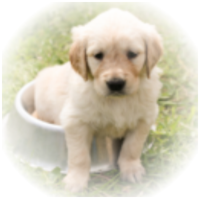

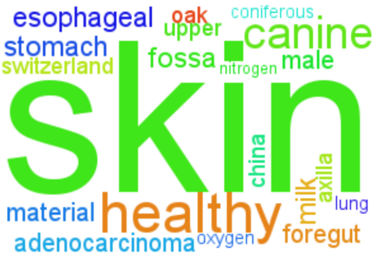

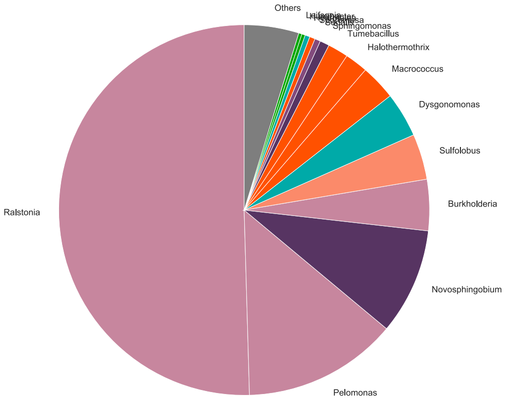

Image from:
https://pixabay.com/en/puppy-golden-retriever-dog-1207816/

Topic #61

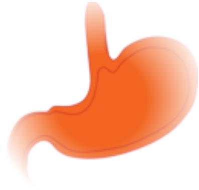

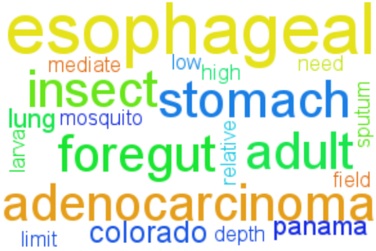

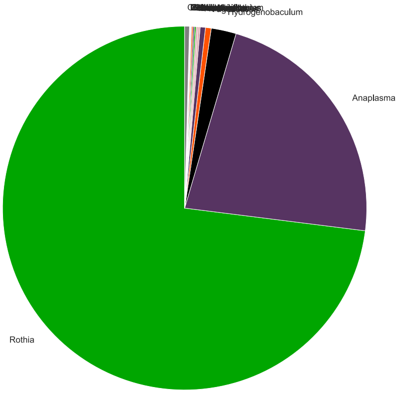

Image from:
https://pixabay.com/en/stomach-anatomy-human-body-biology-310730/

Topic #62

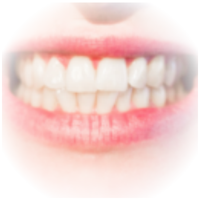

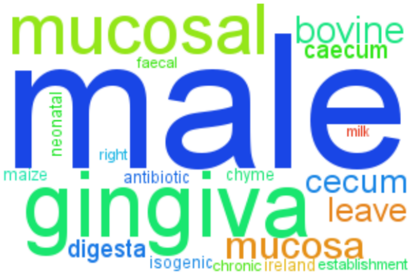

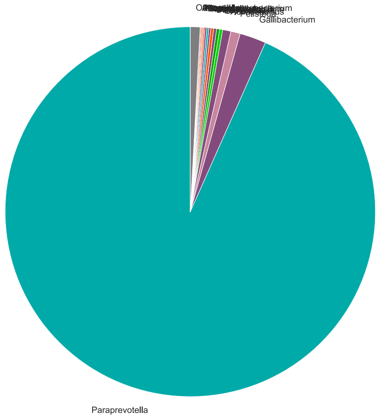

Image from:
https://pixabay.com/en/teeth-dentist-dental-mouth-tooth-1652976/

Topic #63

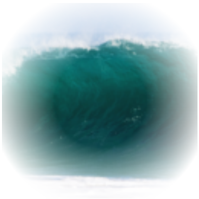

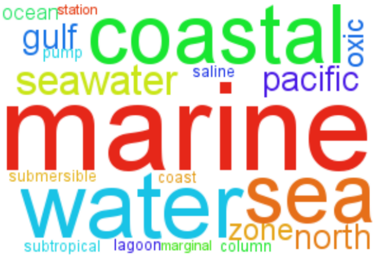

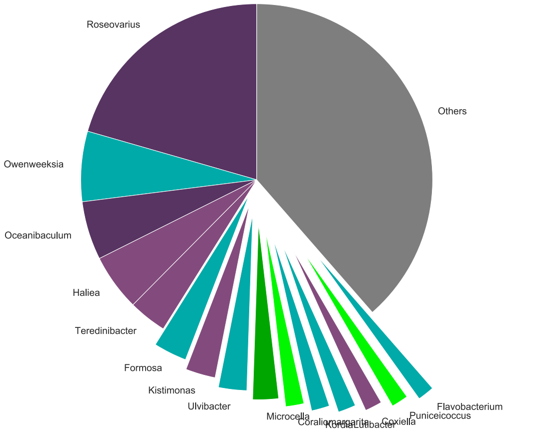

Image from:
https://pixabay.com/en/ocean-wave-sea-water-tide-tidal-918999/

Topic #64

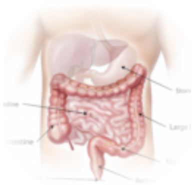

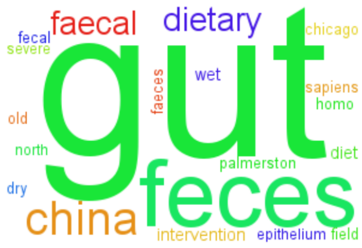

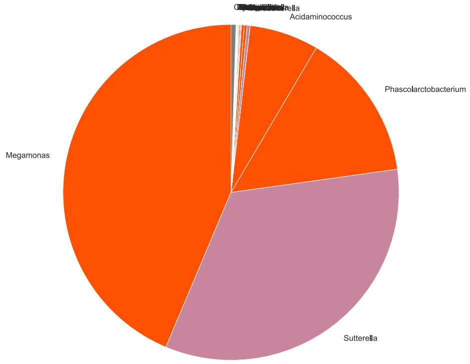

Image from:
https://pixabay.com/en/abdomen-intestine-large-small-1698565/

Topic #65

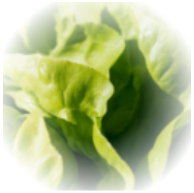

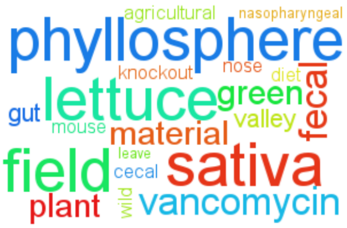

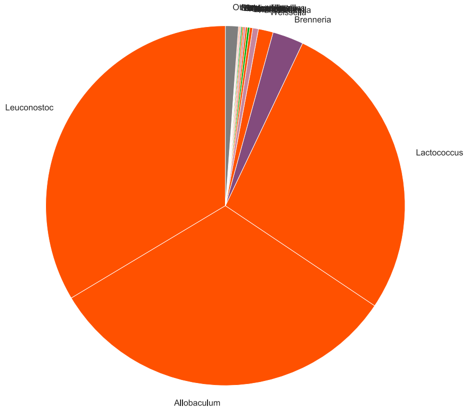

Image from:
https://pixabay.com/en/salad-green-leaf-lettuce-garden-1710328/

Topic #66

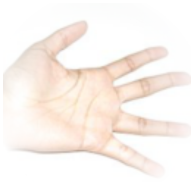

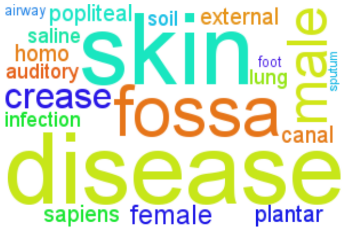

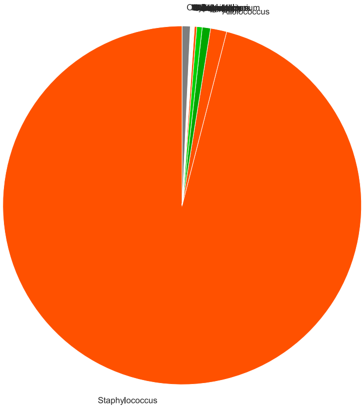

Image from:
https://commons.wikimedia.org/wiki/File%3AHuman_Hand.JPG

Topic #67

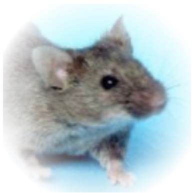

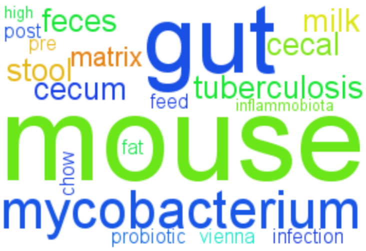

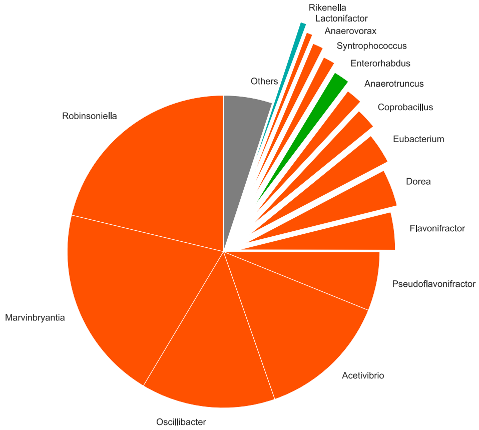

Image from:
https://commons.wikimedia.org/wiki/File%3AHouse_mouse.jpg

Topic #68

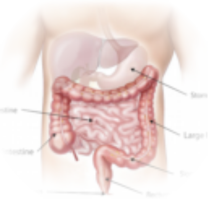

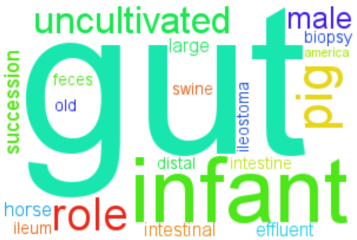

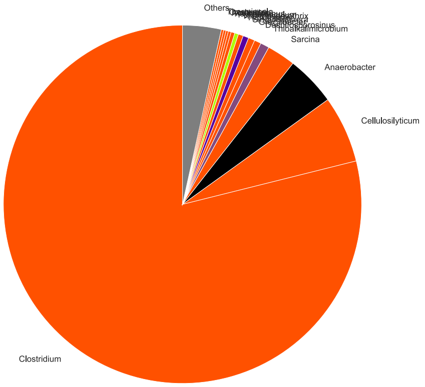

Image from:
https://pixabay.com/en/abdomen-intestine-large-small-1698565/

Topic #69

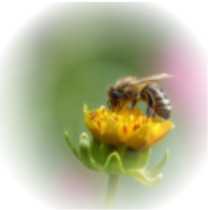

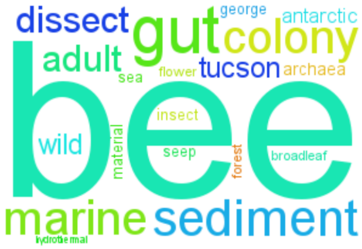

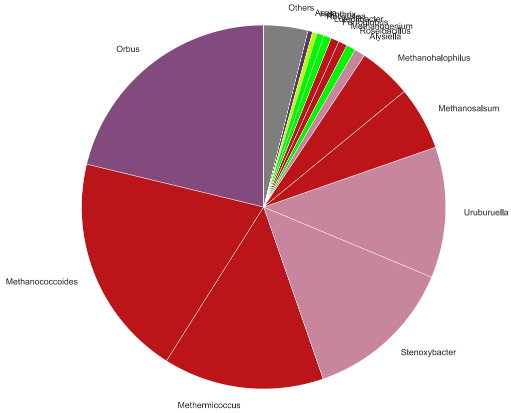

Image from:
https://pixabay.com/en/animal-insect-honey-bee-bee-summer-1685865/

Topic #70

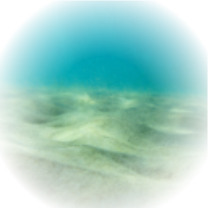

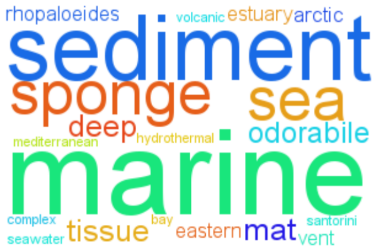

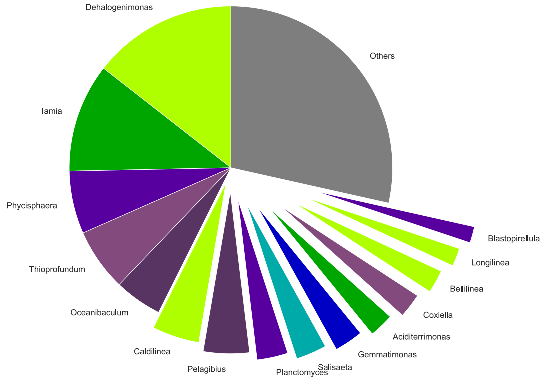

Image from:
https://pixabay.com/en/background-blue-floor-ocean-sand-17520/

Topic #71

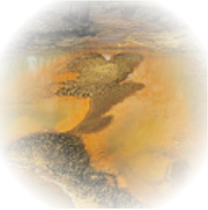

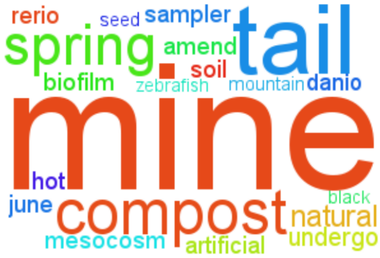

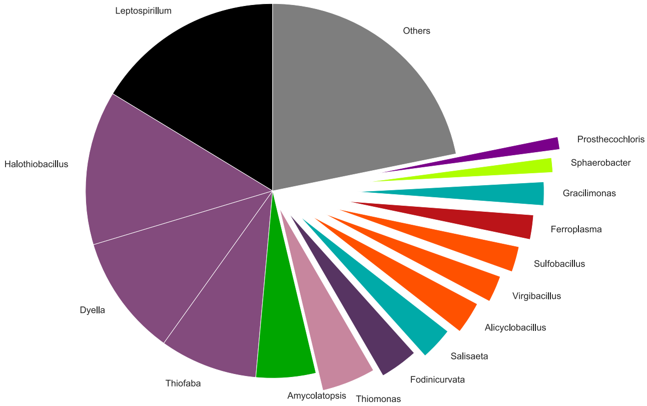

Image from:
https://commons.wikimedia.org/wiki/File%3AIron_hydroxide_precipitate_in_stream.jpg

Topic #72

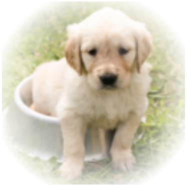

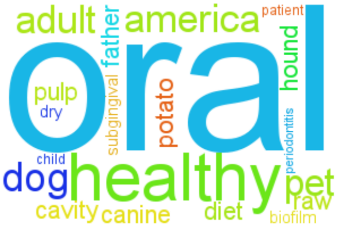

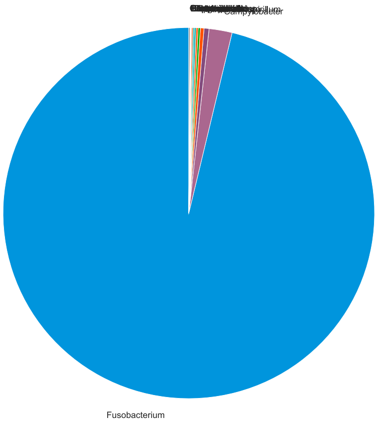

Image from:
https://pixabay.com/en/puppy-golden-retriever-dog-1207816/

Topic #73

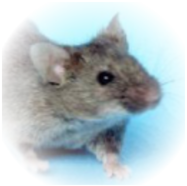

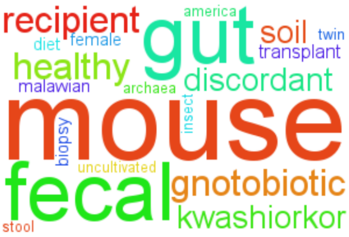

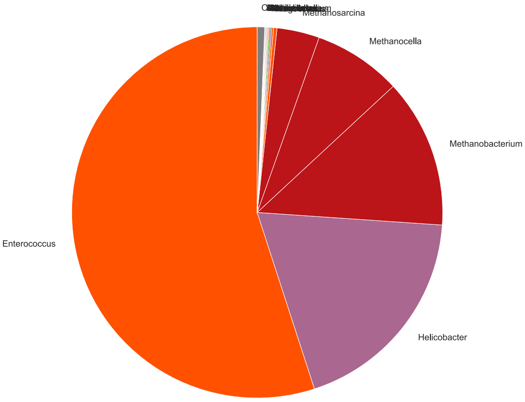

Image from:
https://pixabay.com/en/puppy-golden-retriever-dog-1207816/

Topic #74

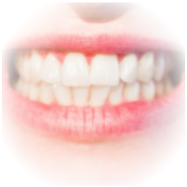

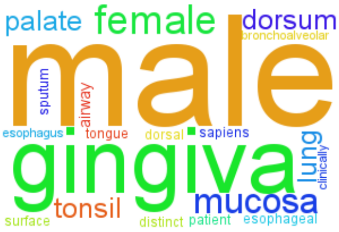

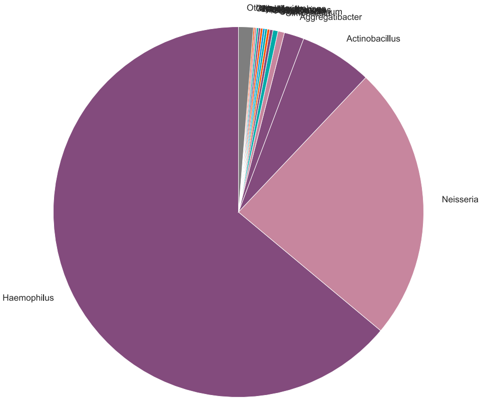

Image from:
https://pixabay.com/en/teeth-dentist-dental-mouth-tooth-1652976/

Topic #75

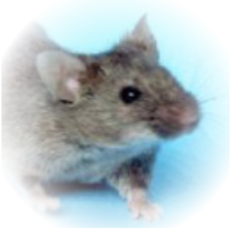

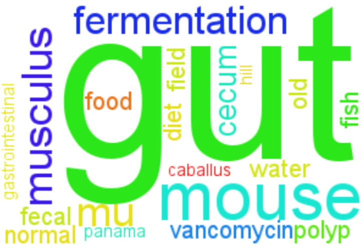

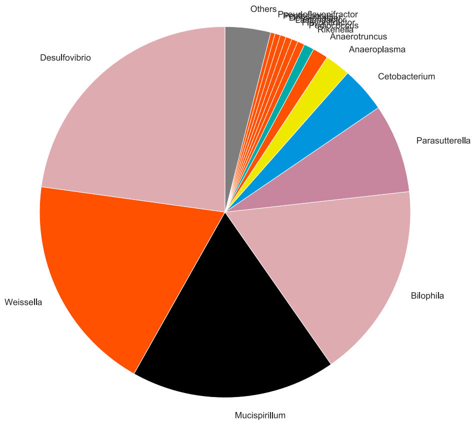

Image from:
https://pixabay.com/en/teeth-dentist-dental-mouth-tooth-1652976/

Topic #76

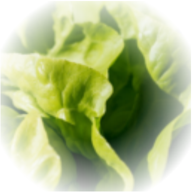

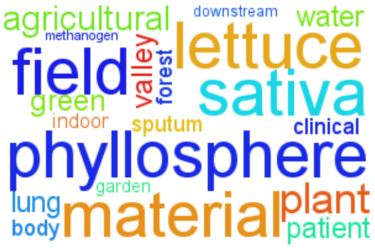

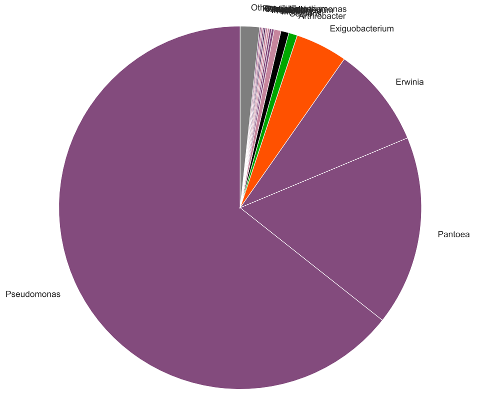

Image from:
https://pixabay.com/en/salad-green-leaf-lettuce-garden-1710328/

Topic #77

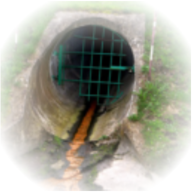

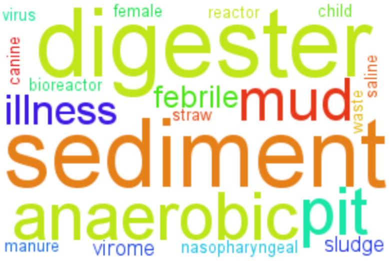

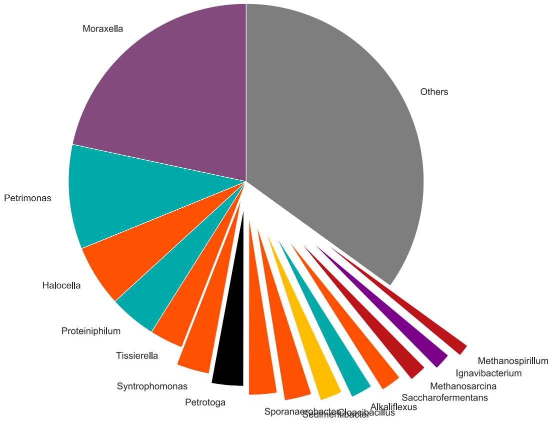

Image from:
https://pixabay.com/en/channel-sewage-sludge-unsanitary-1692671/

Topic #78

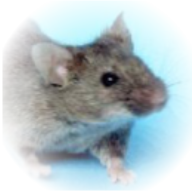

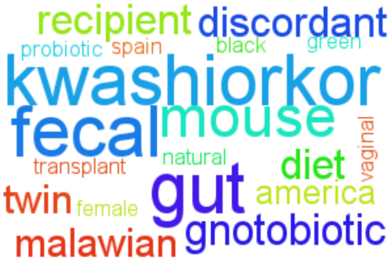

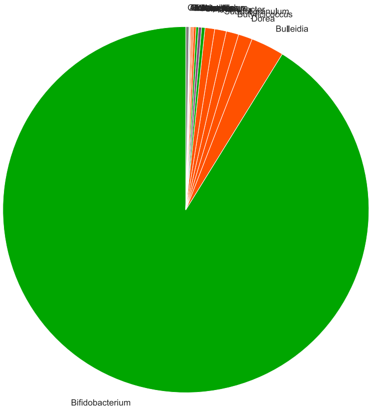

Image from:
https://commons.wikimedia.org/wiki/File%3AHouse_mouse.jpg

Topic #79

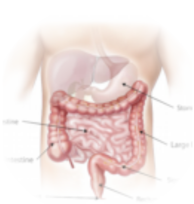

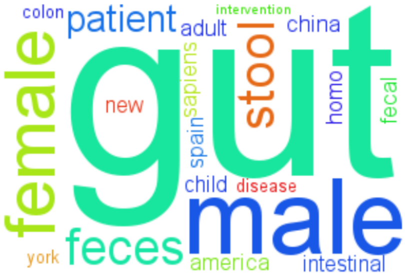

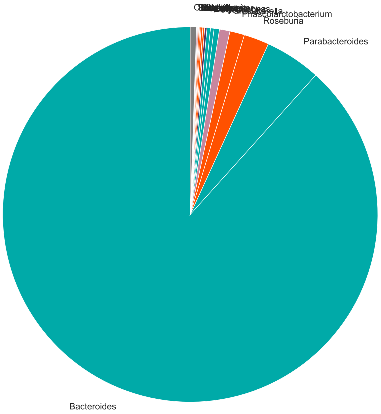

Image from:
https://pixabay.com/en/abdomen-intestine-large-small-1698565/
