# Supplementary material for: Latent environment allocation of microbial community data
Source: PLoS Comput Biol. 2018 Jun 6;14(6):e1006143. doi: 10.1371/journal.pcbi.1006143 (PMC6005635; doi:10.1371/journal.pcbi.1006143)
Supplement: S4 Fig — LEA mapping of the time series of gut microbiome samples from subjects A and B on the global map. The maps of the daily gut microbial community structures retrieved from two individuals are shown (data from David et al.; PRJEB6518). Most of the samples were mapped to “the area of the gut” as learned by pre-existing samples. (Top) The color gradation represents when the samples were taken from the gut of each subject. (Bottom) The samples (colored dots) on the map are colored according to their associated times. Samples during a particular event (traveling and infection) are highlighted by the star-shaped plots. The trajectory of samples is indicated by a dotted line. The dark gray dots on the map represent the samples used for training. (A) The map of the gut microbiome of subject A. The gut environment of subject A was very stable and had a similar taxonomic composition and topic composition over much of the year. However, this stability was disturbed when subject A traveled from his home in an urban area in the United States to Southeast Asia where he stayed from day 72 to day 122 and ate the local diet. Most of the samples that lay outside the cluster are those collected during the subject’s stay in Southeast Asia. We assume that the disturbance of the gut microbiome of the subject during his stay in Southeast Asia reflects his exposure to the local diet and environment. As a comparison, gut microbiomes taken from 106 healthy Japanese subjects are mapped as pink dots (data from Nishijima et al.; PRJDB3601). Interestingly, at the beginning of subject A's stay in Southeast Asia, the positions of some of his samples were near those of the Japanese subjects (Day 87). (B) The map of the gut microbiome of subject B. The topic composition of the subject was stable and near topic #28 during the first half of the year. However, because subject B was infected with Salmonella between days 151 and 159, the microbial sample taken on the day 155 mapped near topic #68. After re [file pcbi.1006143.s004.pdf]

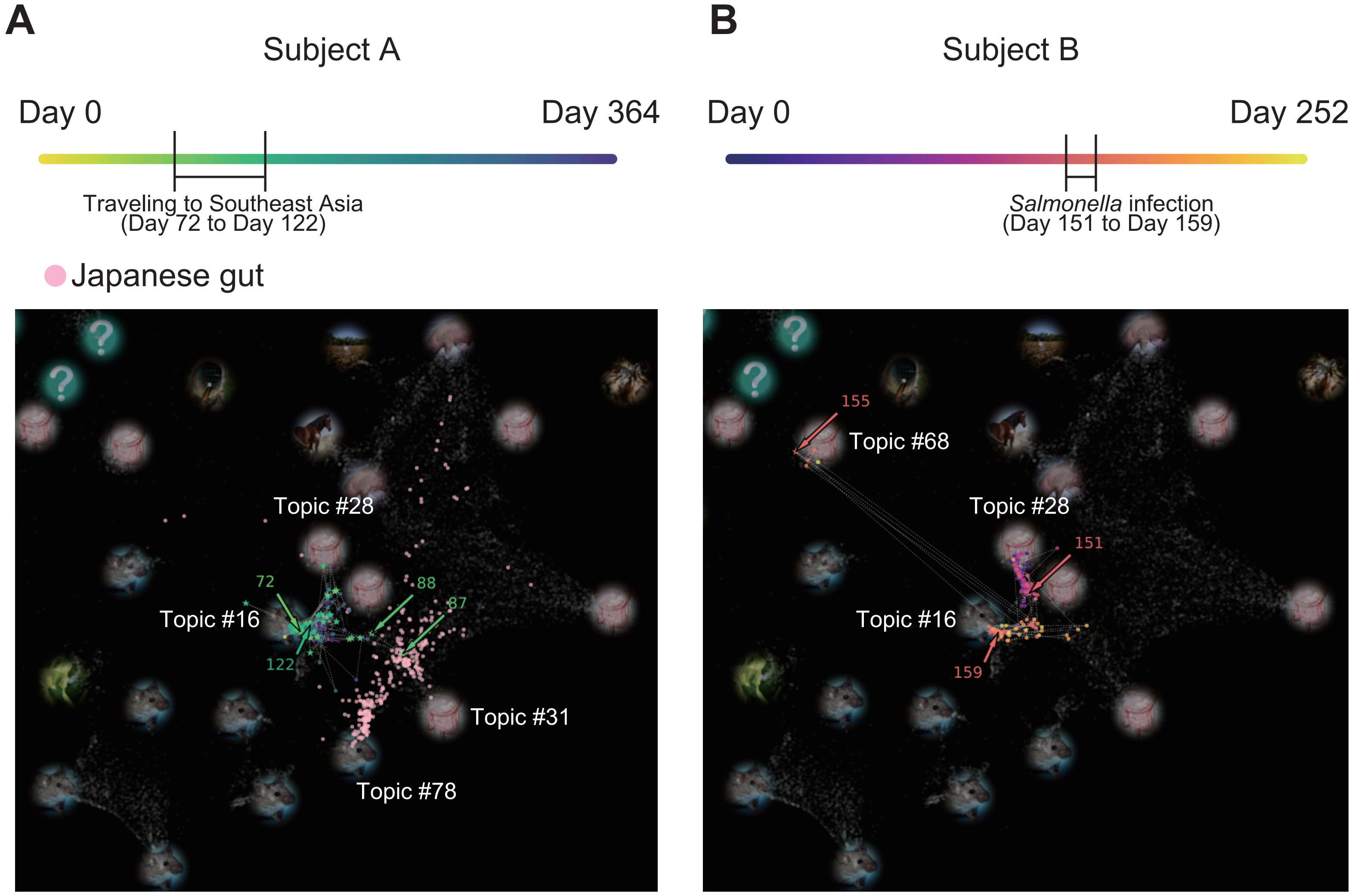
A
Subject A
Day 0
Day 364
Traveling to Southeast Asia
(Day 72 to Day 122)
Japanese gut
Topic #28
Topic #16
Topic #31
Topic #78
72
88
87
122
B
Subject B
Day 0
Day 252
Salmonella infection
(Day 151 to Day 159)
155
Topic #68
Topic #28
151
Topic #16
159
