# Supplementary material for: Latent environment allocation of microbial community data
Source: PLoS Comput Biol. 2018 Jun 6;14(6):e1006143. doi: 10.1371/journal.pcbi.1006143 (PMC6005635; doi:10.1371/journal.pcbi.1006143)
Supplement: S9 Fig — The vertical axis shows the proportion of environment-associated topics per sample, and the horizontal axis shows 30,718 samples. Samples were ordered by the proportion of the corresponding topics. (PDF) [file pcbi.1006143.s009.pdf]

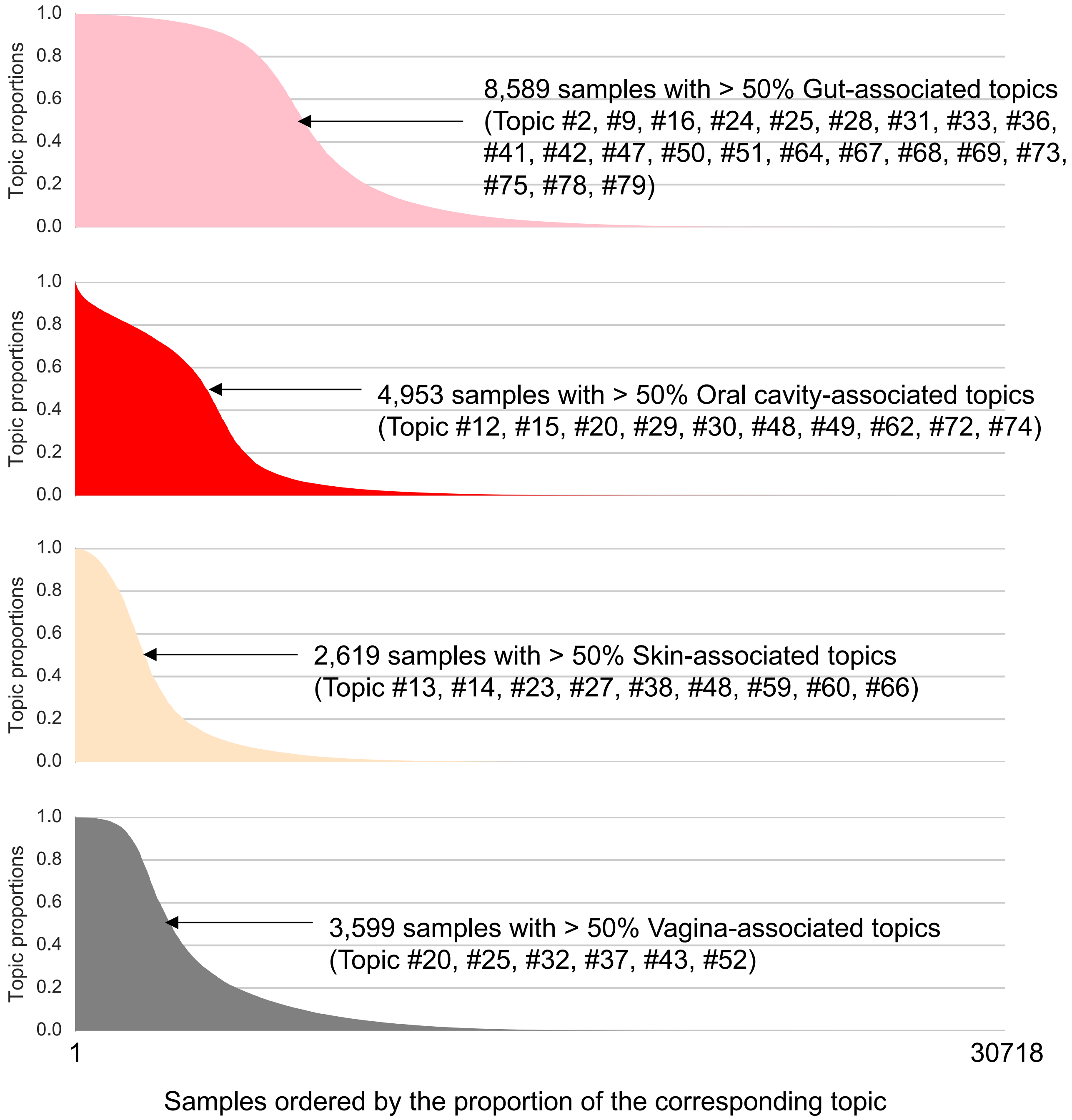

Topic proportions
1.0
0.8
0.6
0.4
0.2
0.0
8,589 samples with > 50% Gut-associated topics
(Topic #2, #9, #16, #24, #25, #28, #31, #33, #36,
#41, #42, #47, #50, #51, #64, #67, #68, #69, #73,
#75, #78, #79)
4,953 samples with > 50% Oral cavity-associated topics
(Topic #12, #15, #20, #29, #30, #48, #49, #62, #72, #74)
2,619 samples with > 50% Skin-associated topics
(Topic #13, #14, #23, #27, #38, #48, #59, #60, #66)
3,599 samples with > 50% Vagina-associated topics
(Topic #20, #25, #32, #37, #43, #52)
1
30718
Samples ordered by the proportion of the corresponding topic

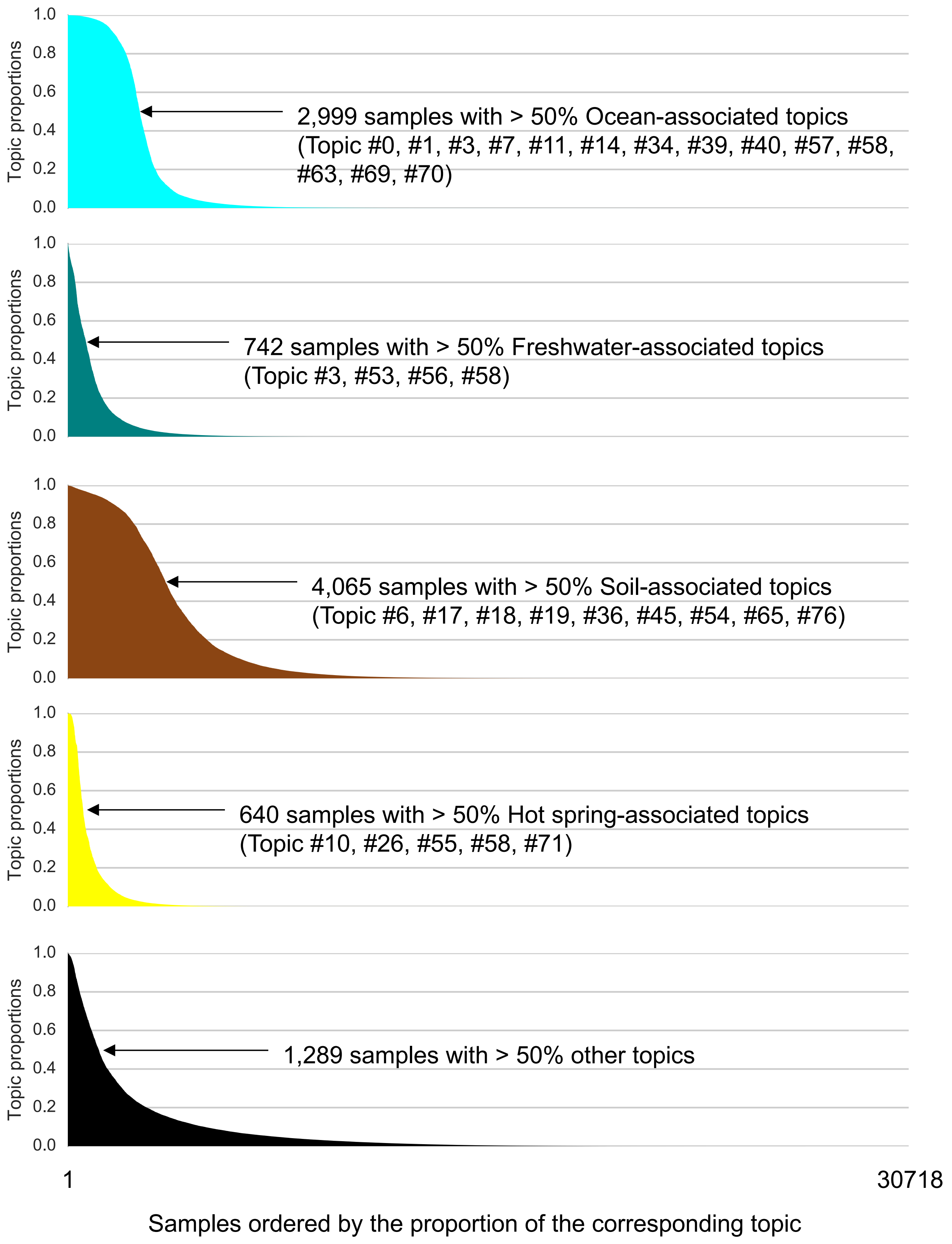

1.0
0.8
0.6
0.4
0.2
0.0
Topic proportions
2,999 samples with > 50% Ocean-associated topics
(Topic #0, #1, #3, #7, #11, #14, #34, #39, #40, #57, #58,
#63, #69, #70)
742 samples with > 50% Freshwater-associated topics
(Topic #3, #53, #56, #58)
4,065 samples with > 50% Soil-associated topics
(Topic #6, #17, #18, #19, #36, #45, #54, #65, #76)
640 samples with > 50% Hot spring-associated topics
(Topic #10, #26, #55, #58, #71)
1,289 samples with > 50% other topics
1
30718
Samples ordered by the proportion of the corresponding topic
