# Supplementary material for: Latent environment allocation of microbial community data
Source: PLoS Comput Biol. 2018 Jun 6;14(6):e1006143. doi: 10.1371/journal.pcbi.1006143 (PMC6005635; doi:10.1371/journal.pcbi.1006143)

A

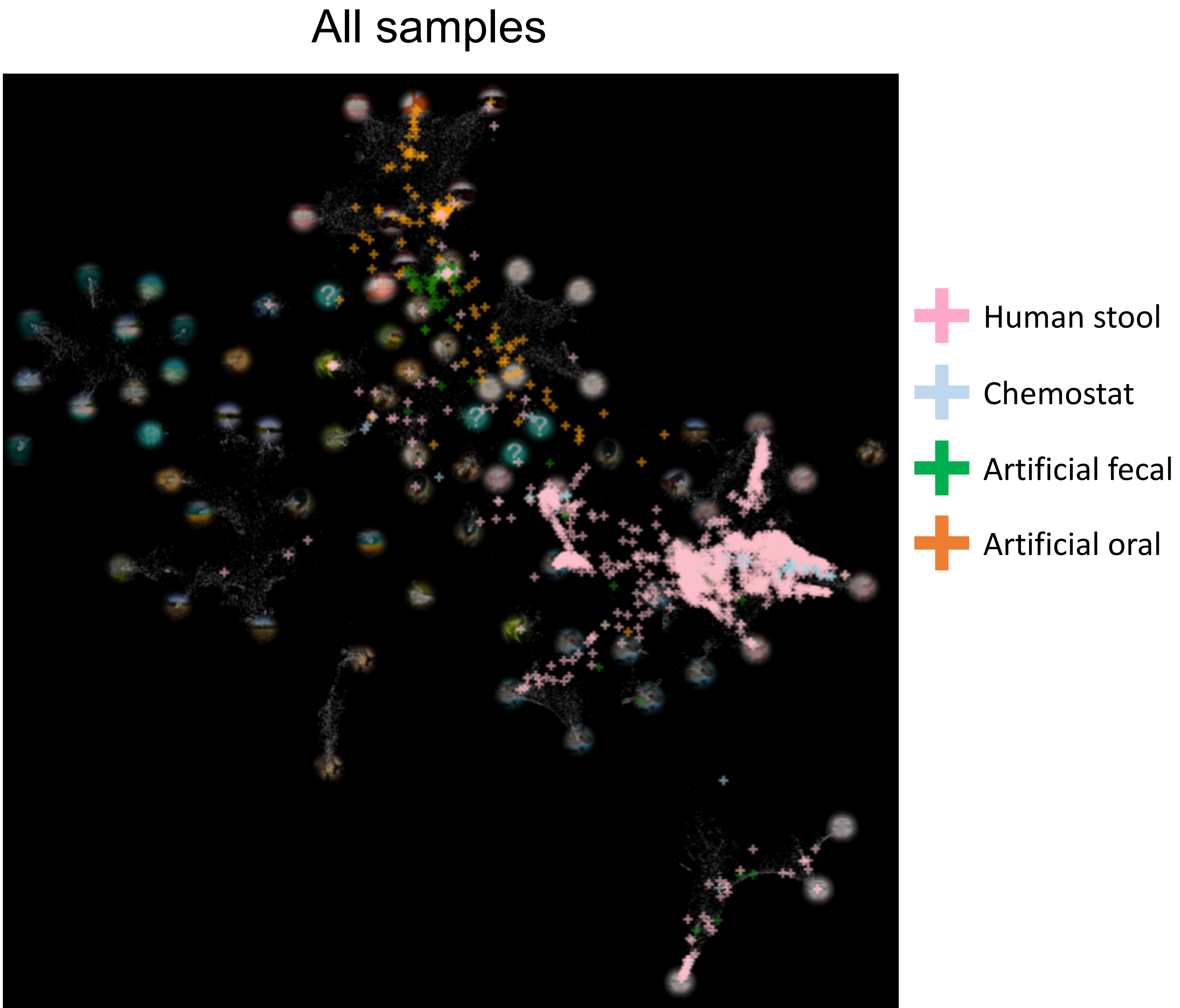
All samples
Human stool
Chemostat
Artificial fecal
Artificial oral

B

Subject D2301

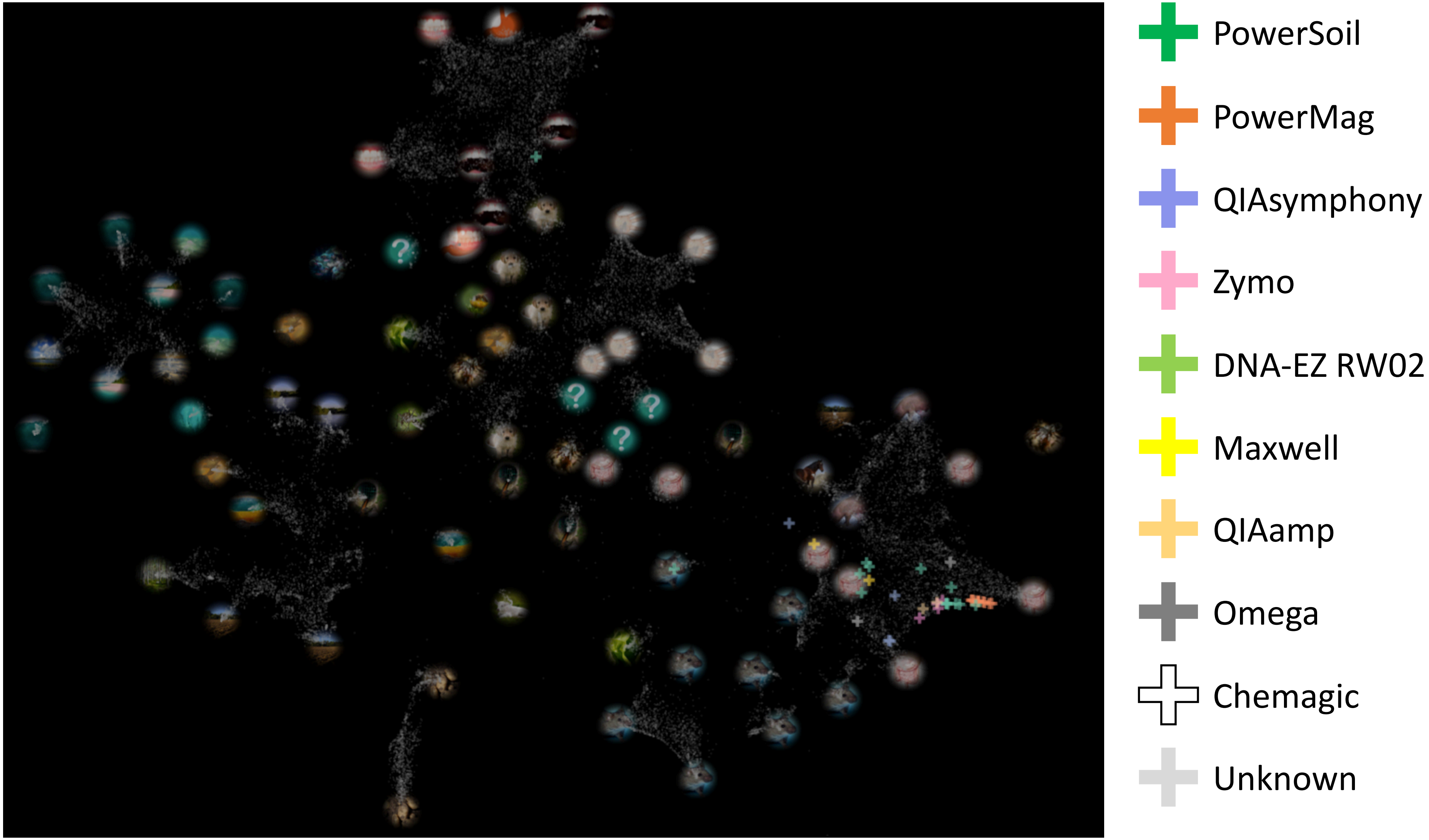

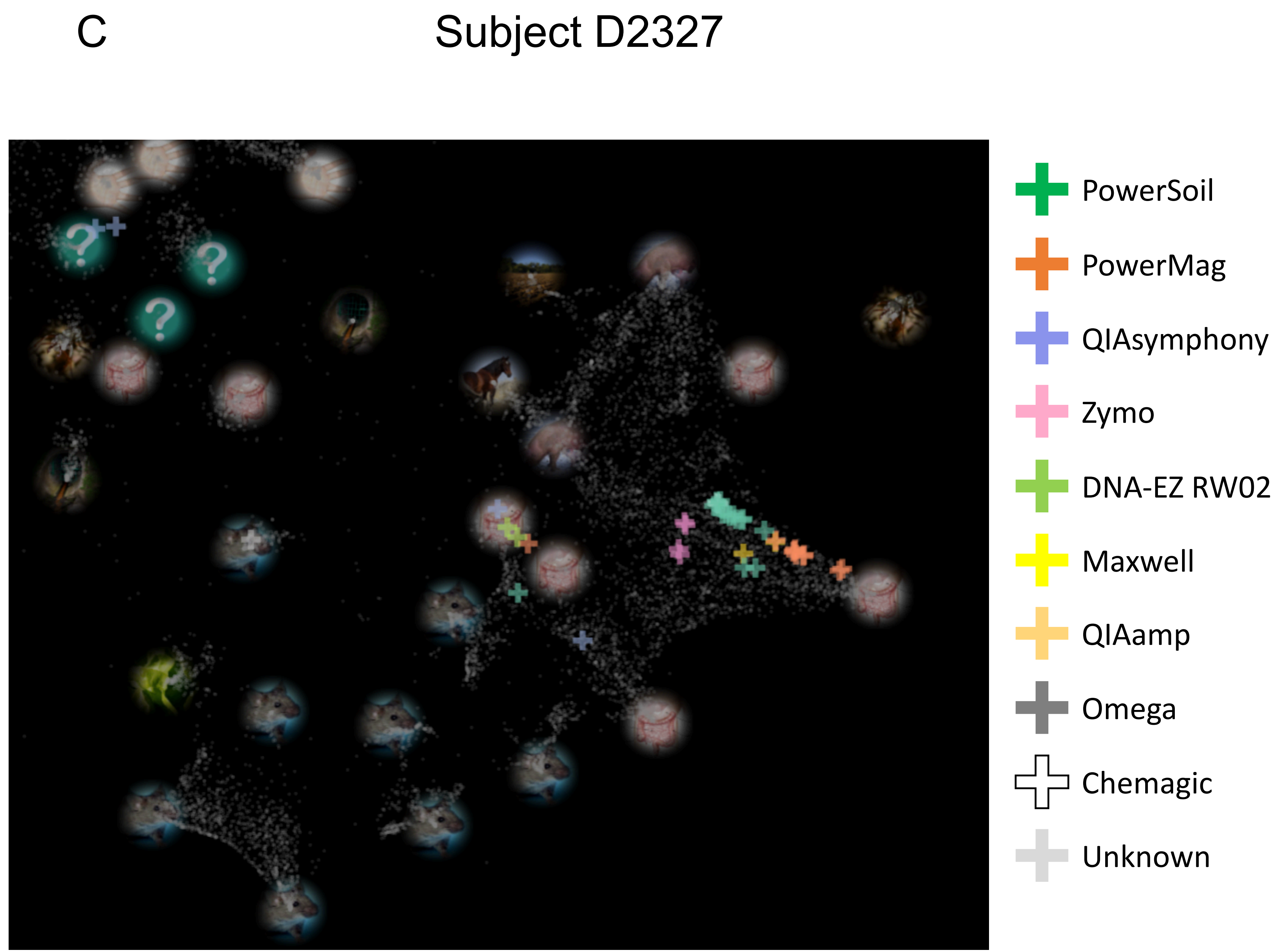
C
Subject D2327
PowerSoil
PowerMag
QIAsymphony
Zymo
DNA-EZ RW02
Maxwell
QIAamp
Omega
Chemagic
Unknown

D

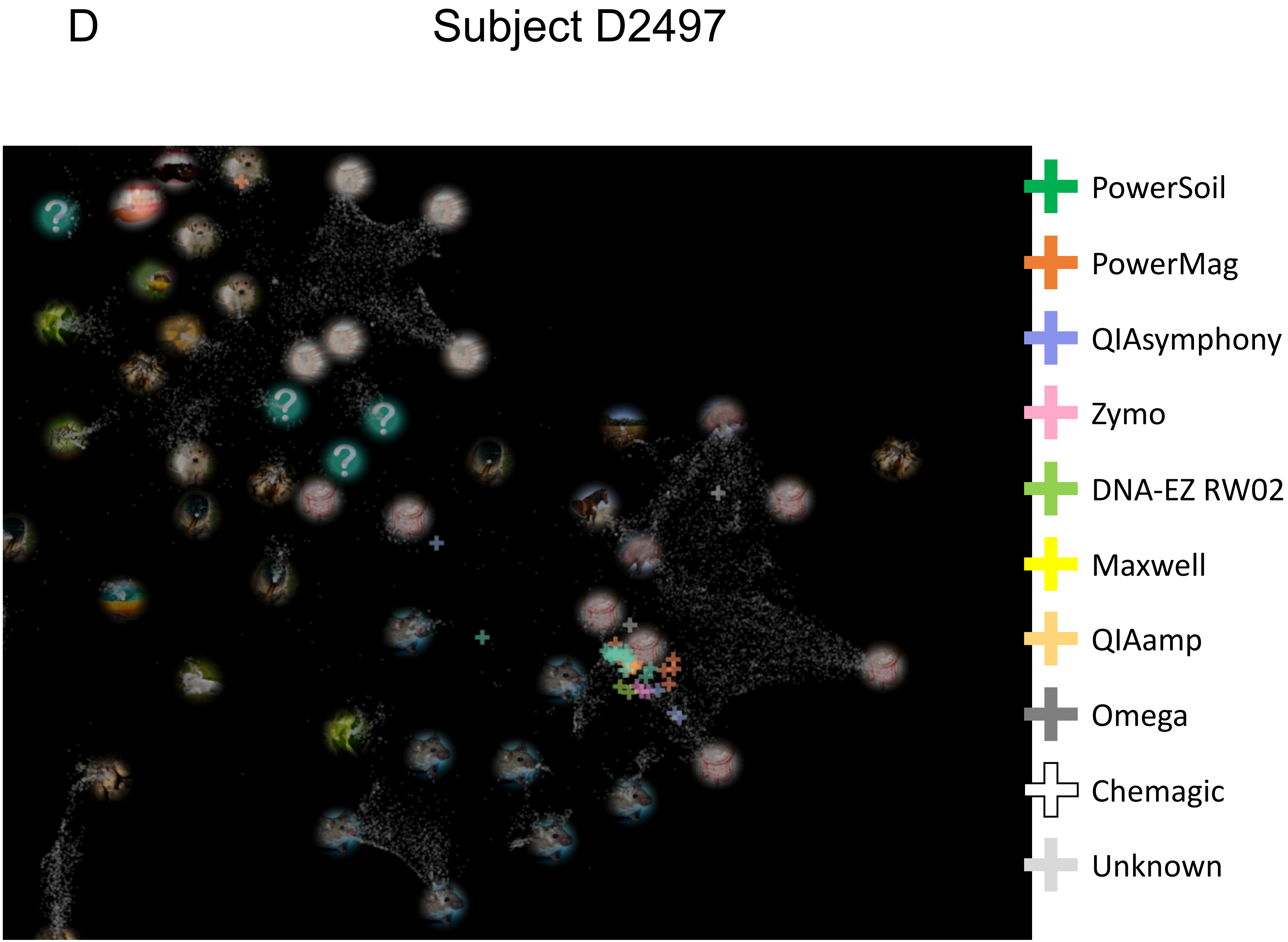

Subject D2497
PowerSoil
PowerMag
QIAsymphony
Zymo
DNA-EZ RW02
Maxwell
QIAamp
Omega
Chemagic
Unknown

E

Subject D2561

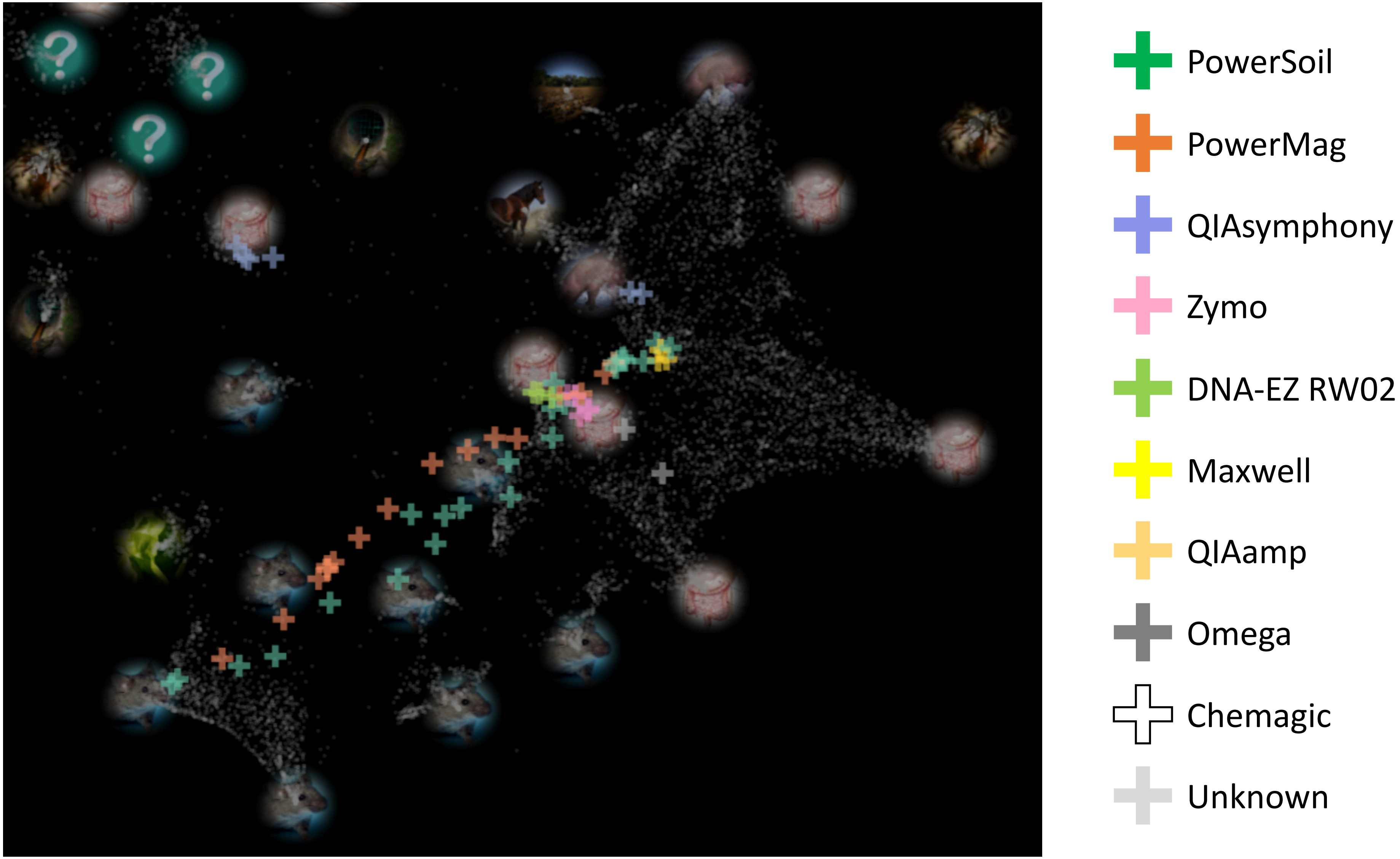

F

Subject D2590

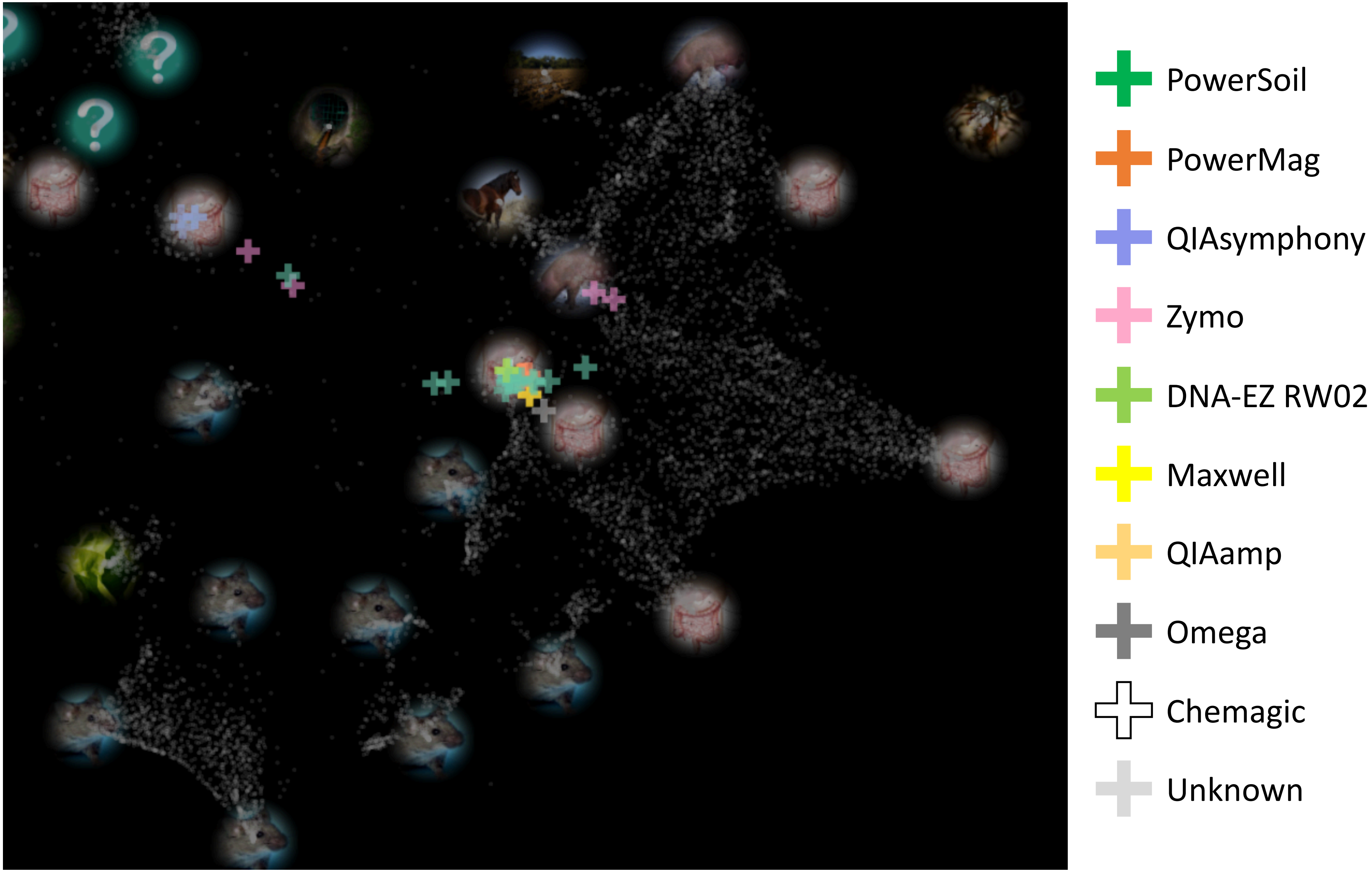

G

Subject D2696

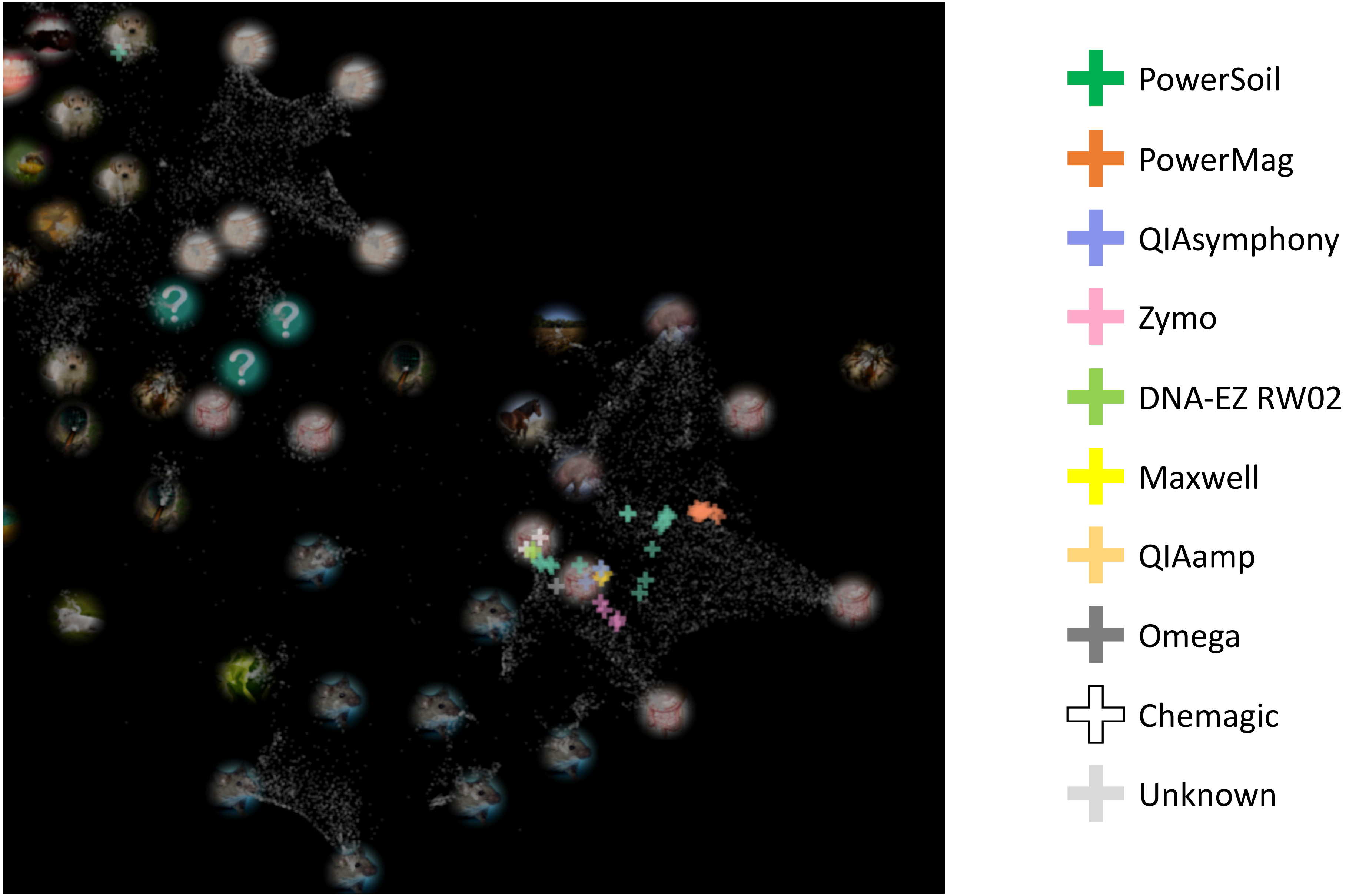

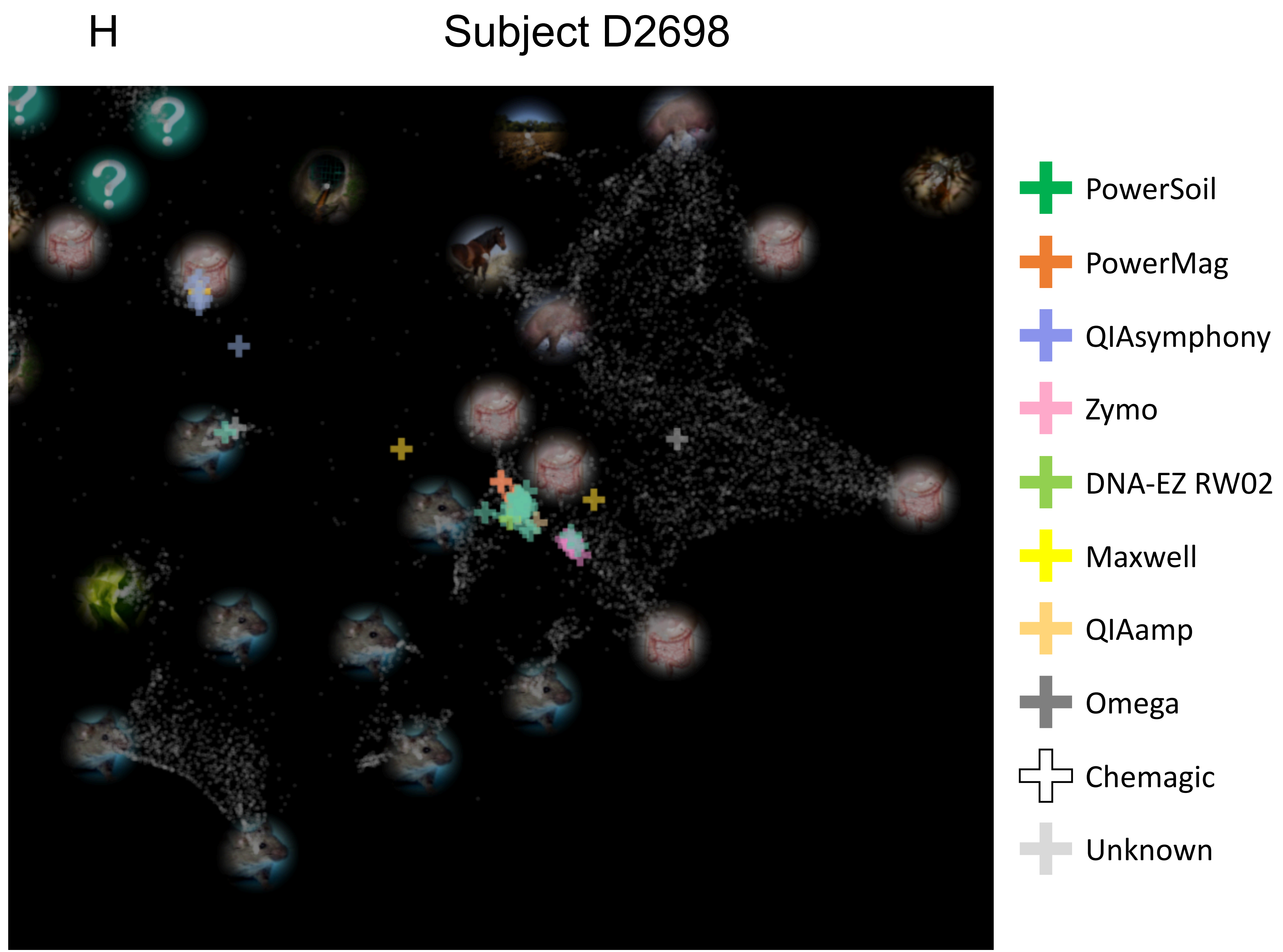
H
Subject D2698
PowerSoil
PowerMag
QIAsymphony
Zymo
DNA-EZ RW02
Maxwell
QIAamp
Omega
Chemagic
Unknown

I

Subject DZ15291

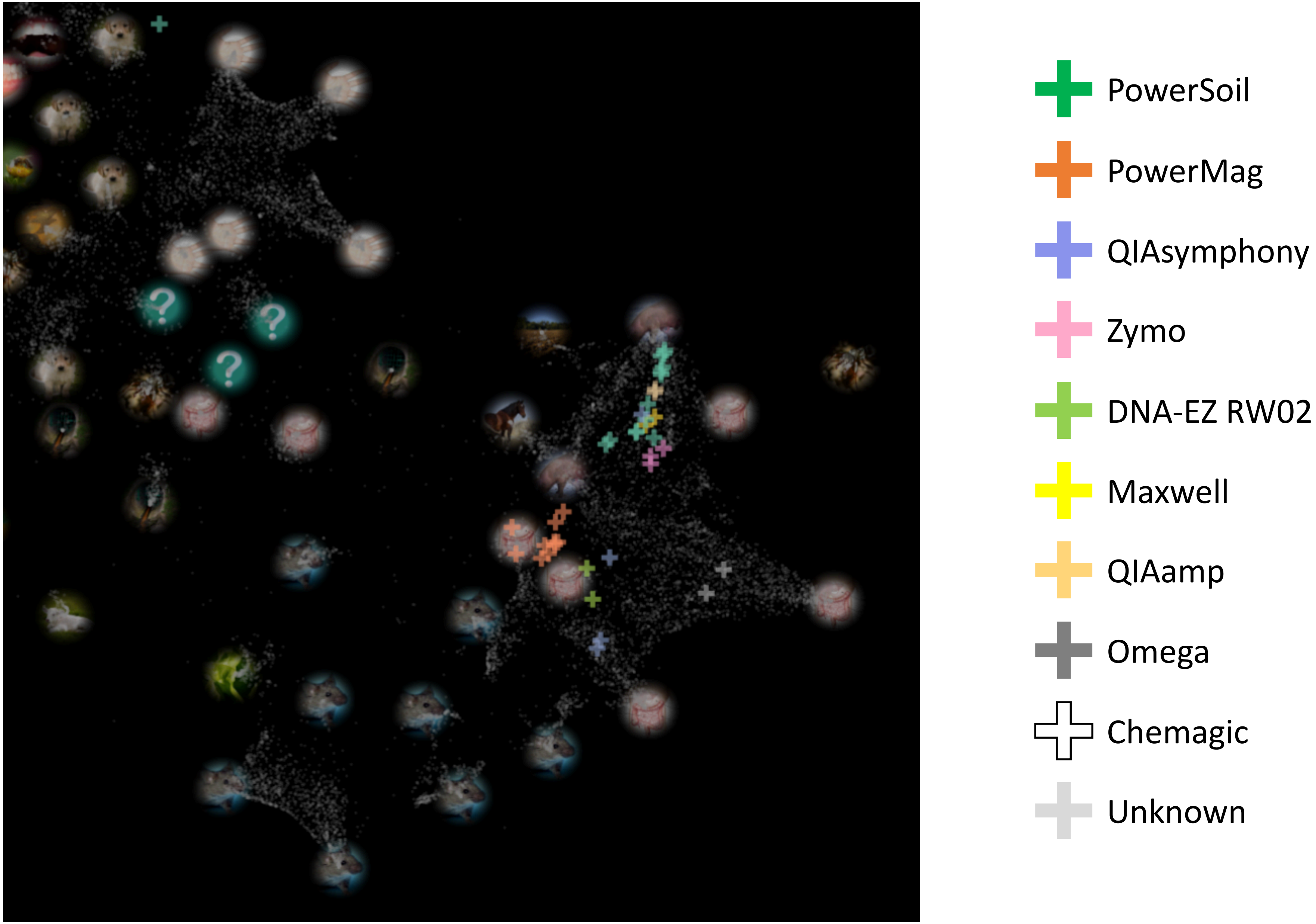

J

Subject DZ15292

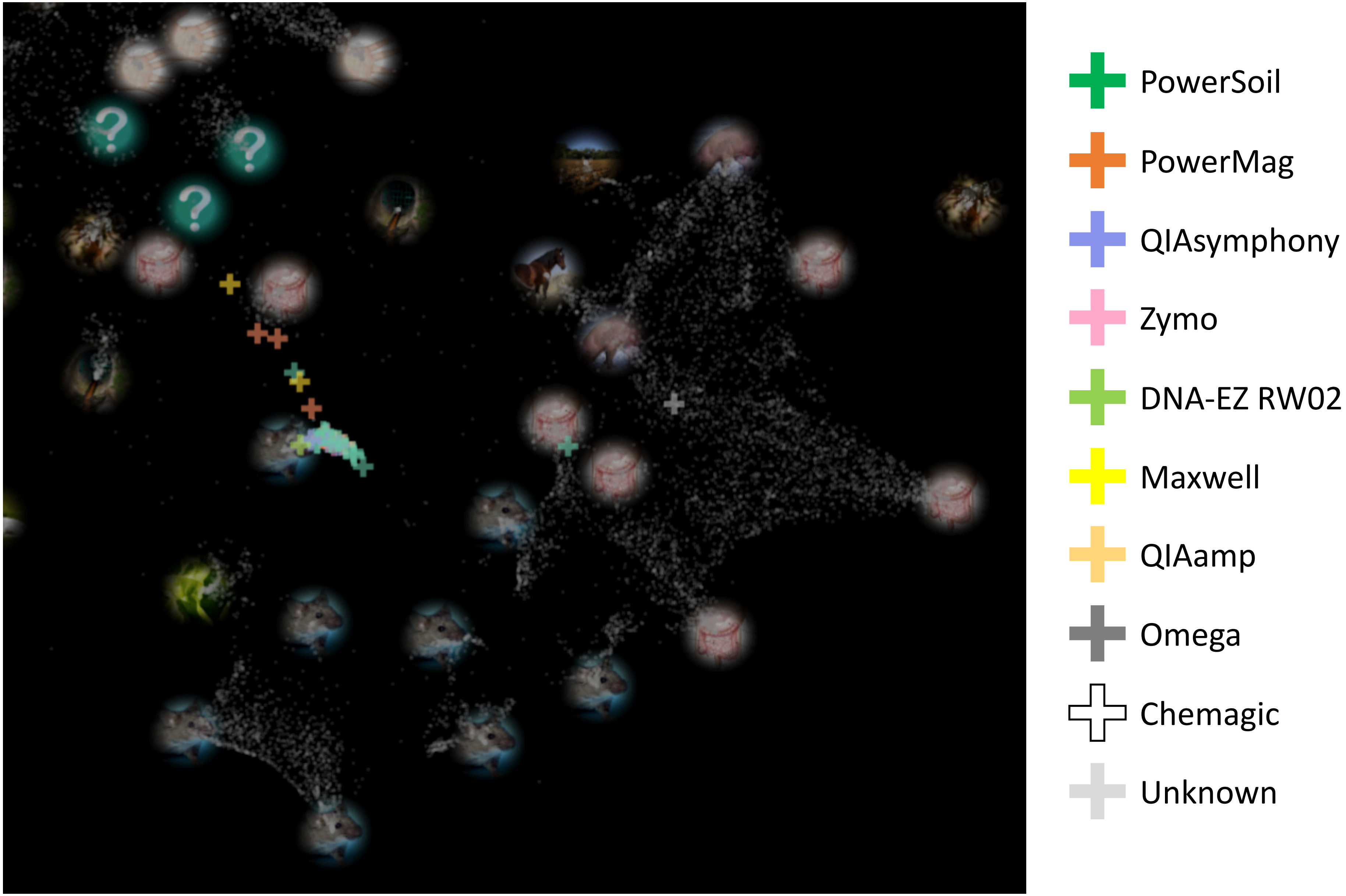

K

Subject DZ15293

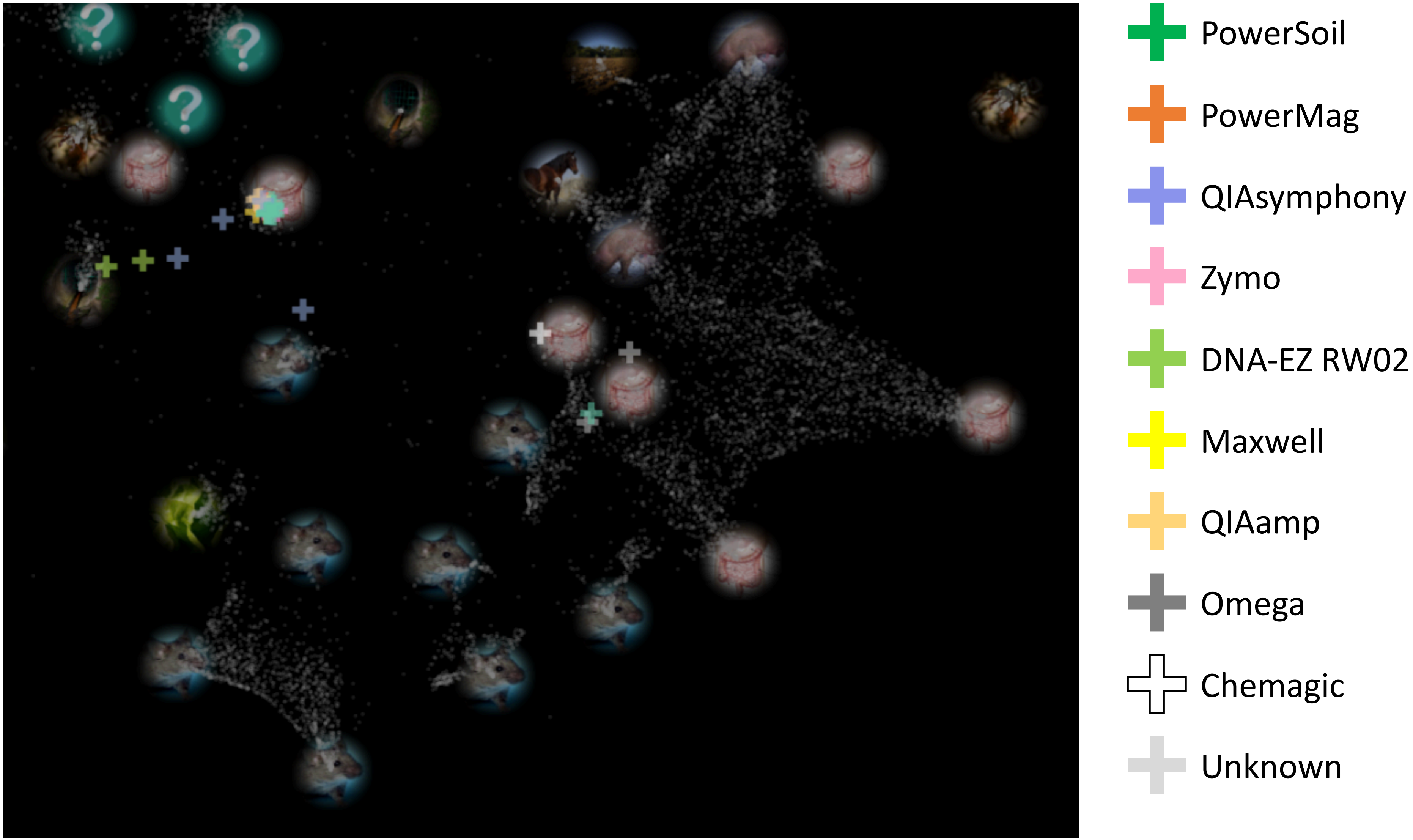

L

Subject DZ15294

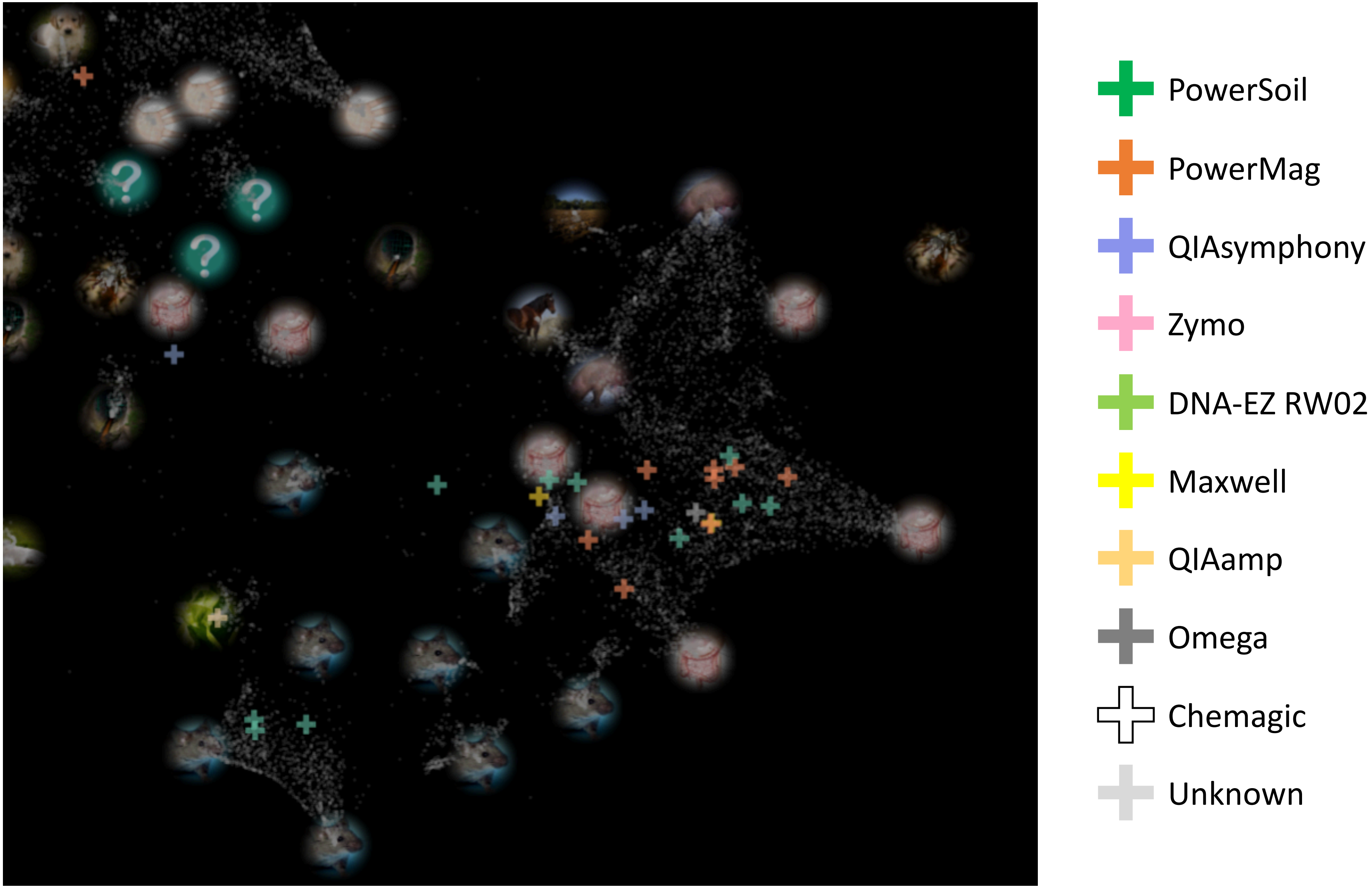

M

Subject DZ15295

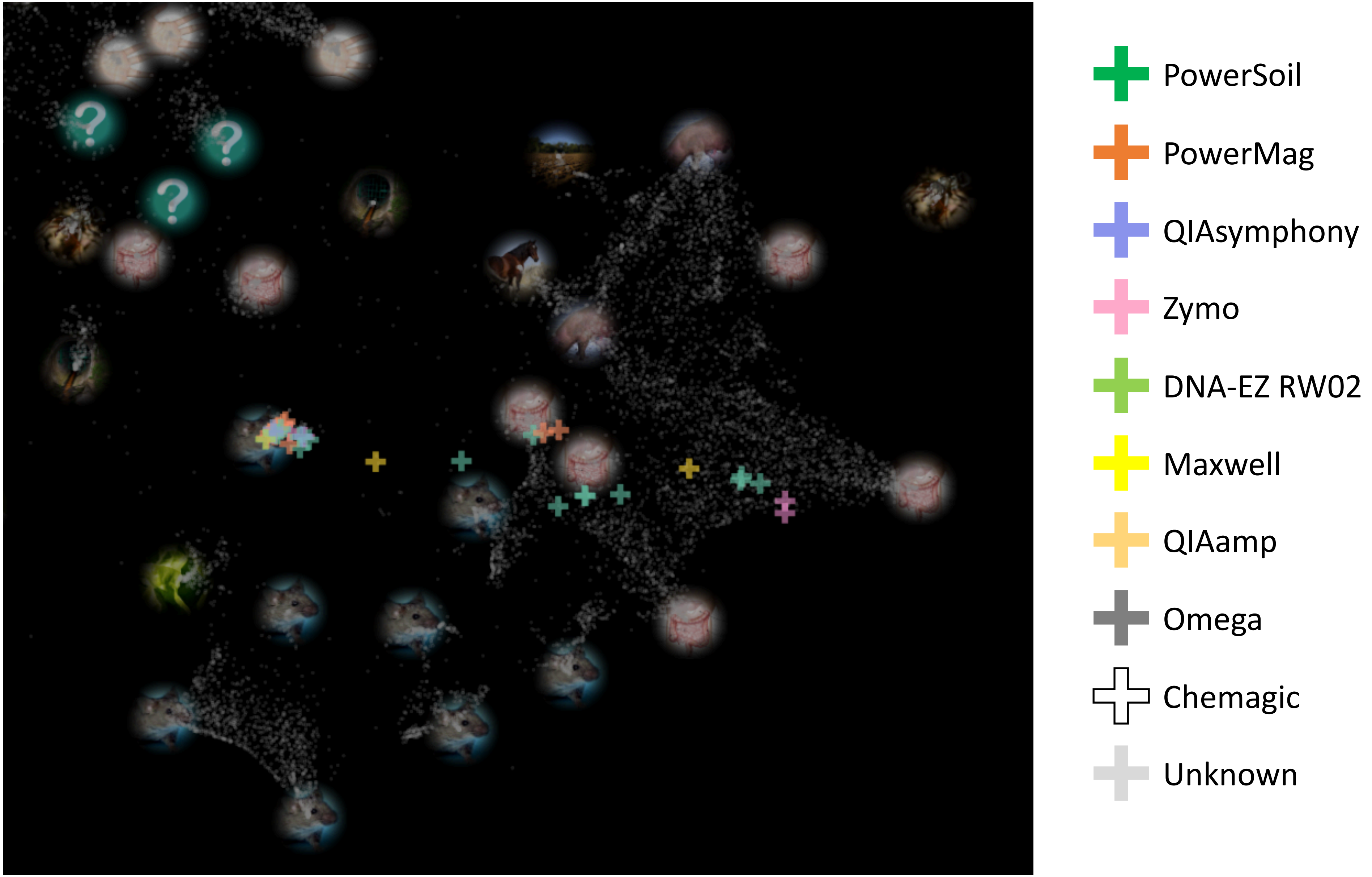

N

Subject DZ15296

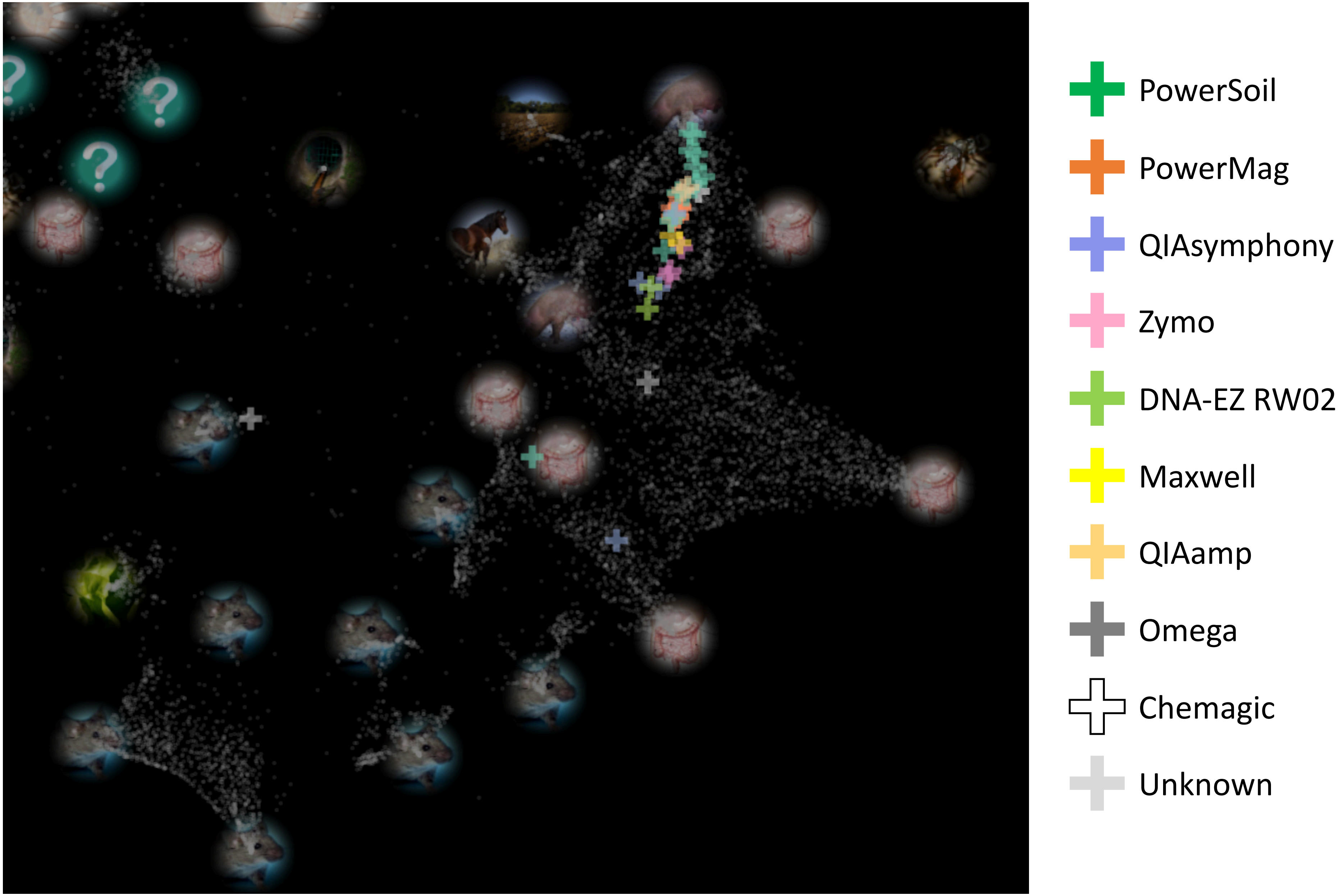

O

Subject DZ15298

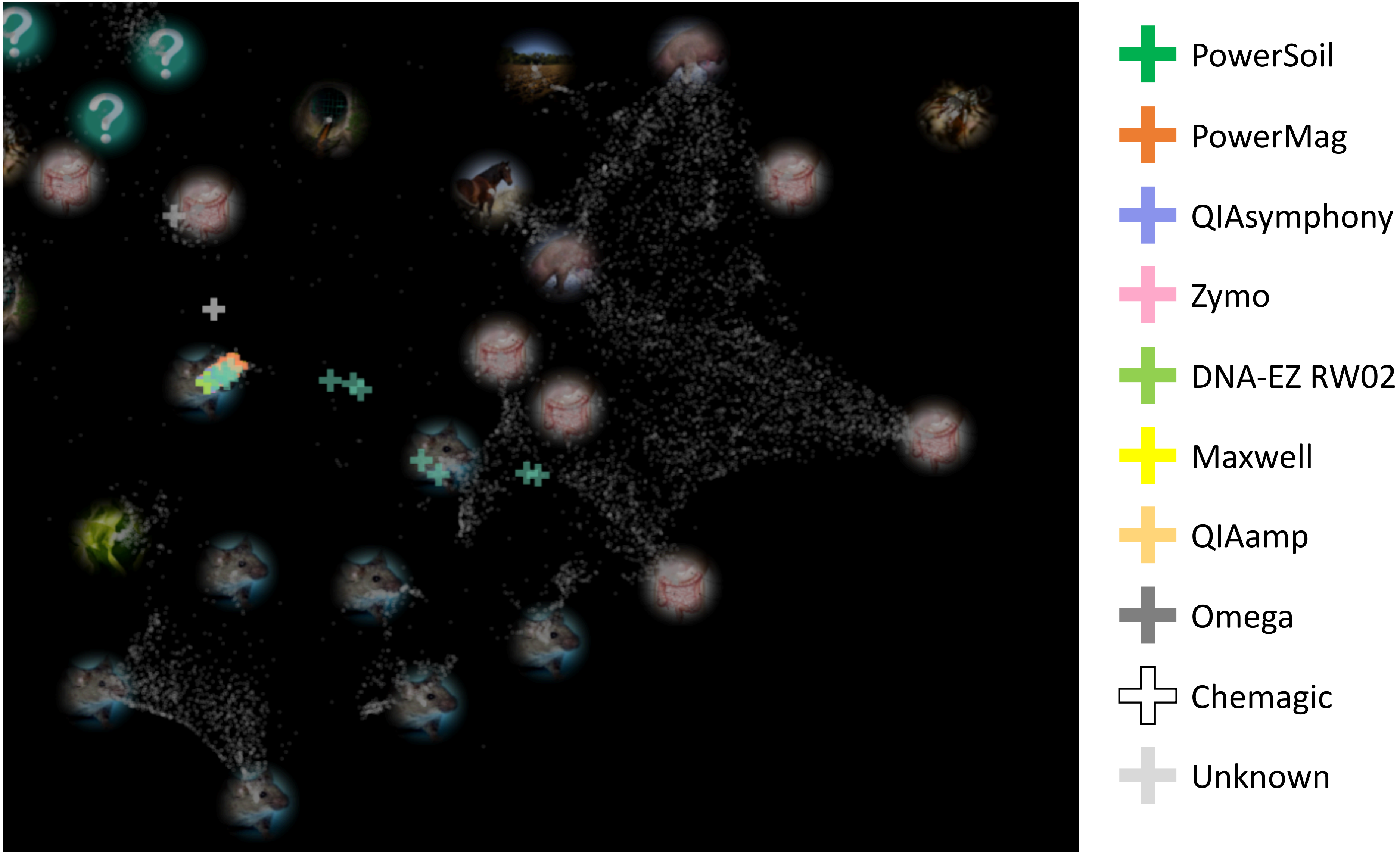

P

Subject DZ15300

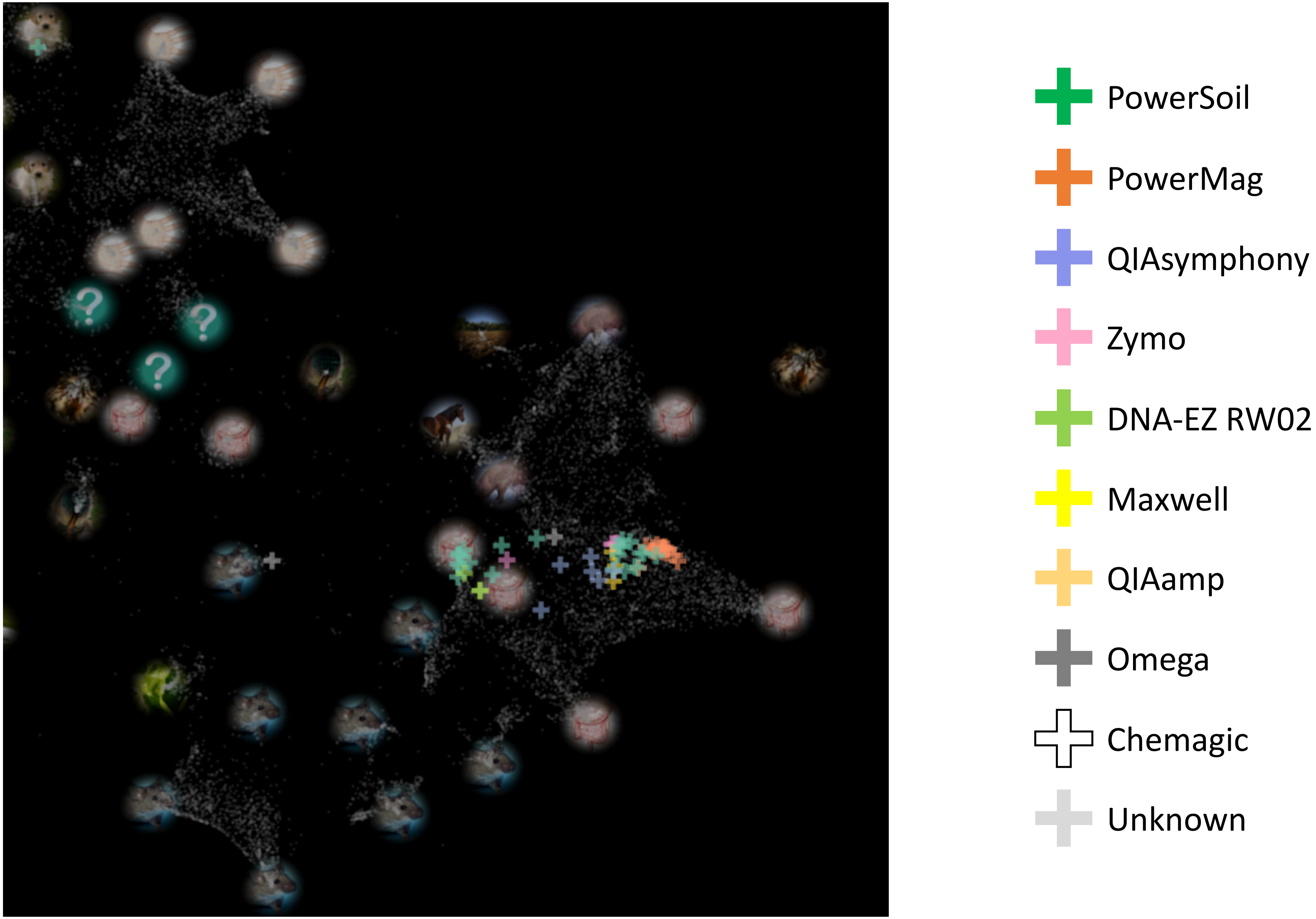

Q

Subject DZ15302

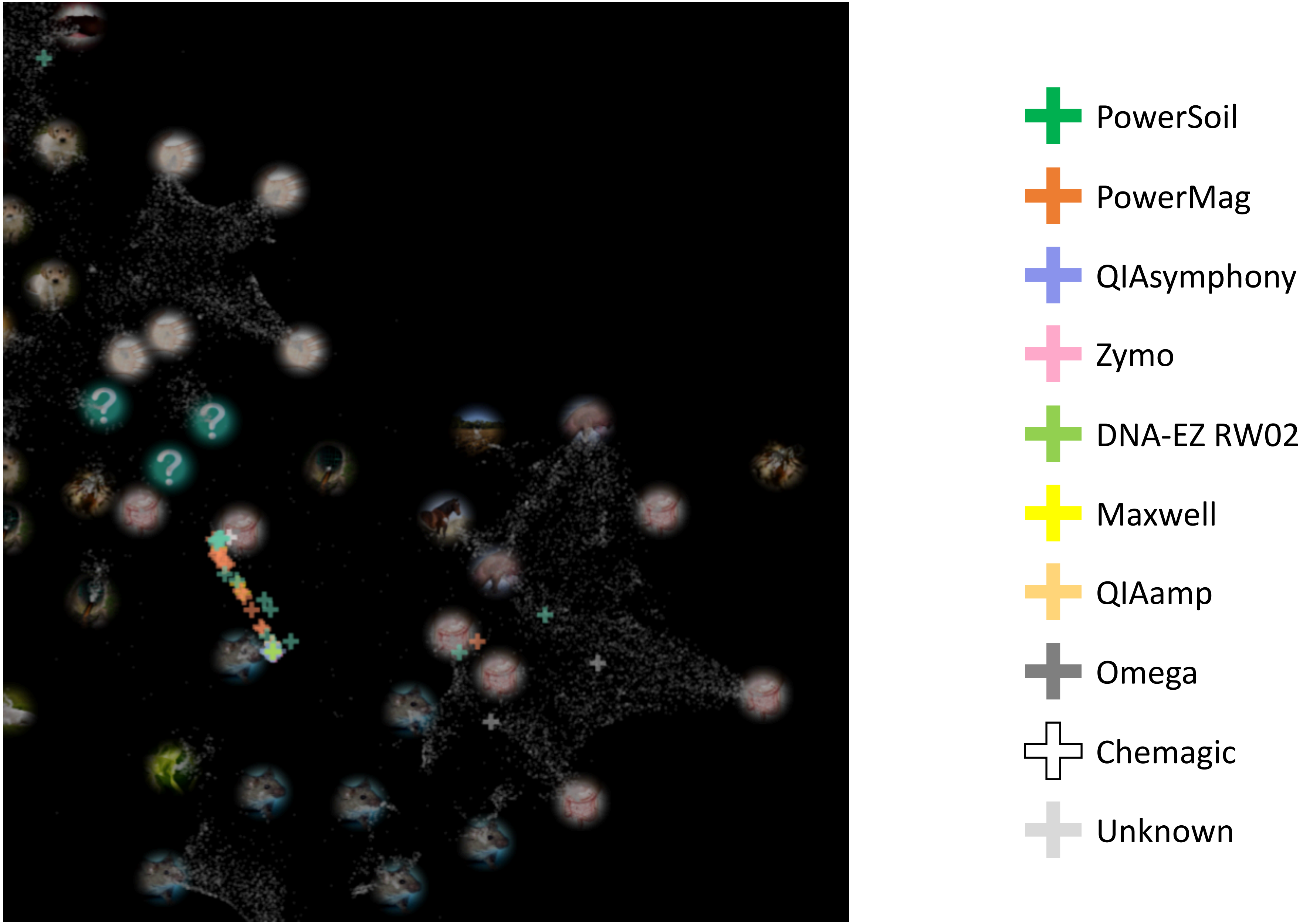

R

Subject DZ15303

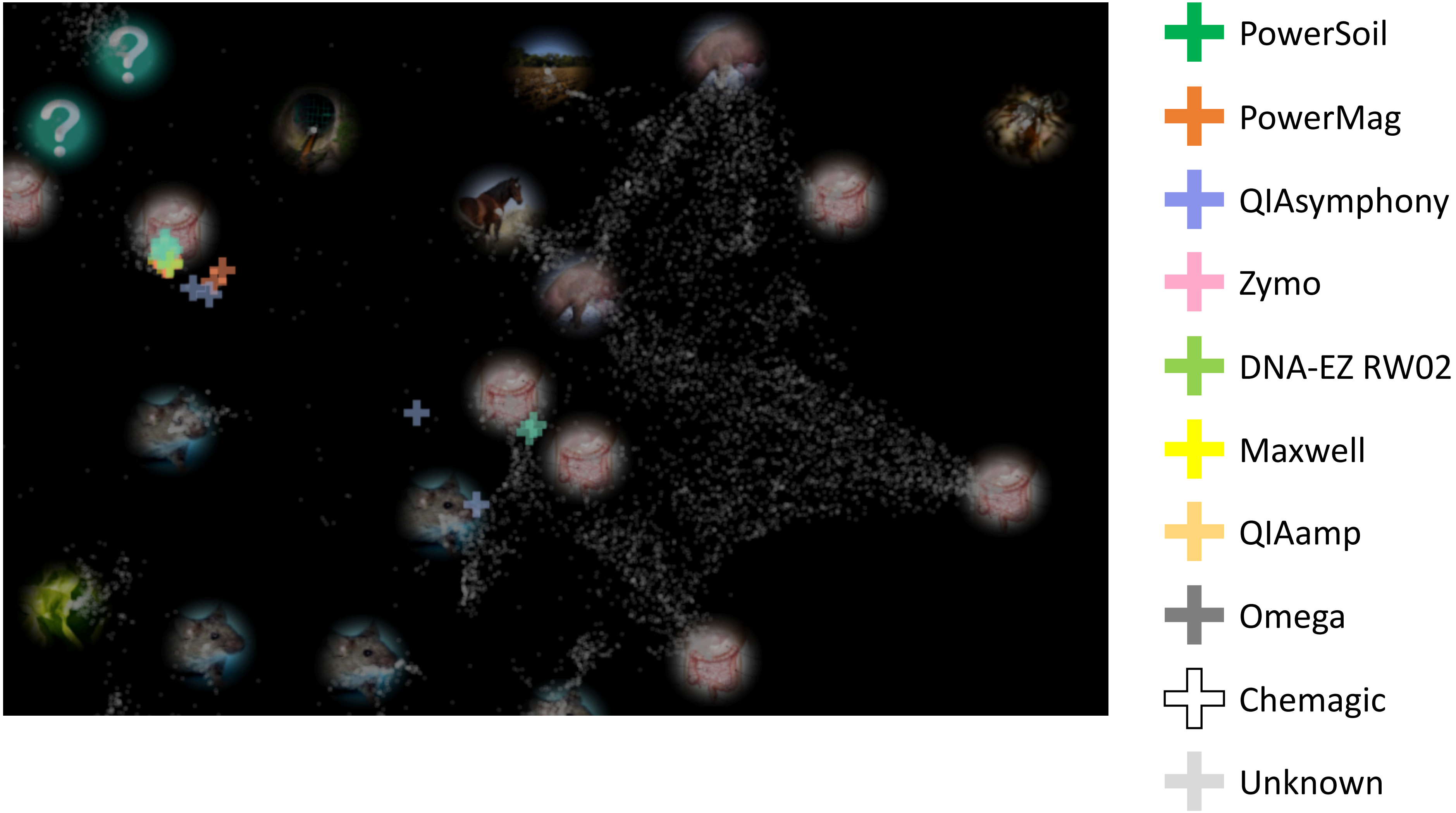

S

Subject DZ15304

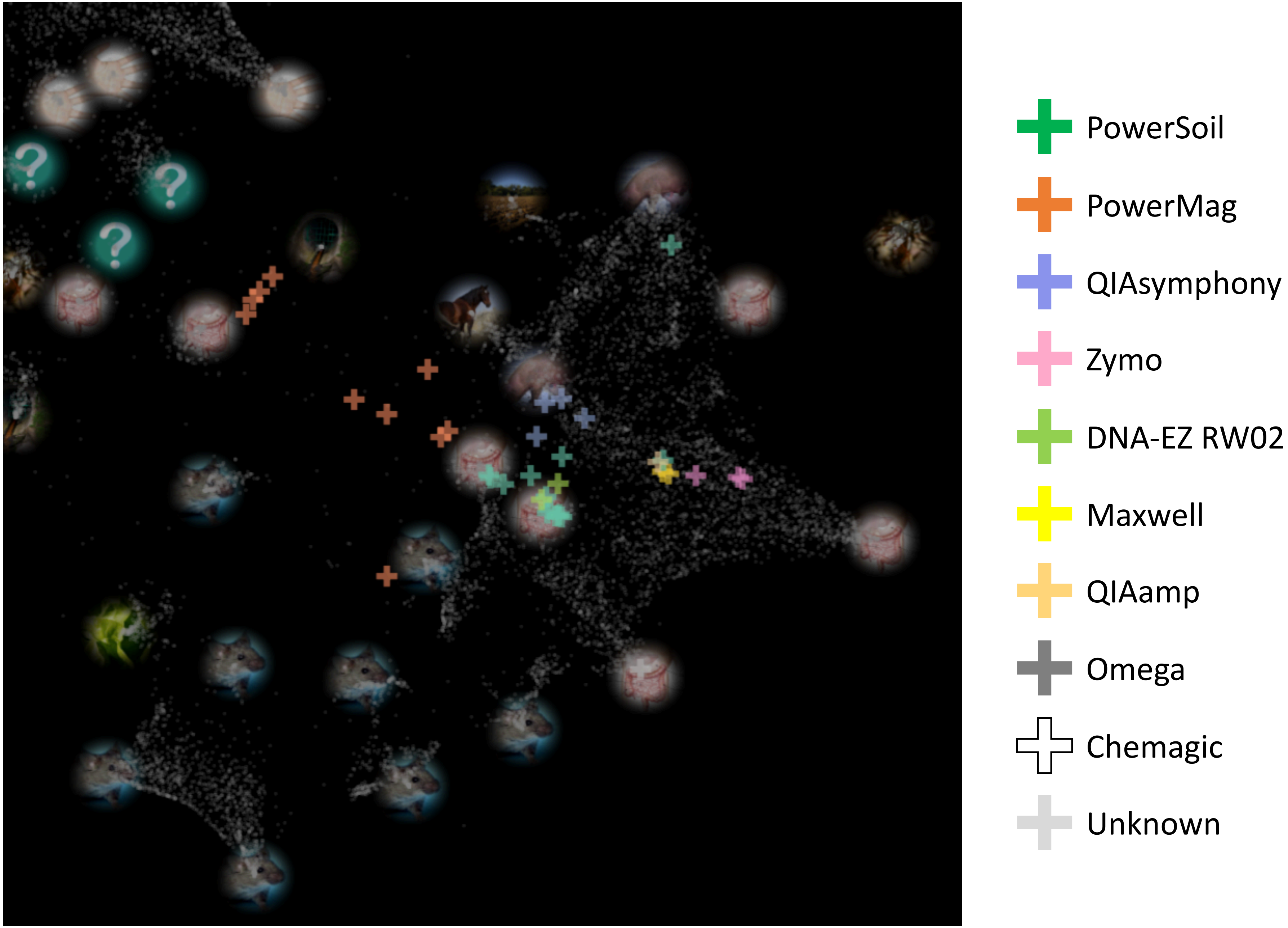

Supplement: S10 Fig — LEA mapping of Microbiome Quality Control (MBQC) dataset. (A) Colored crosses represent all 2,049 samples of Microbiome Quality Control Project on the LEA global map. These contain human-derived samples (pink), chemostat samples (light blue), artificial fecal communities (green), and artificial oral communities (orange). (B-S) LEA mapping results of samples for each subject. Colored crosses indicate samples derived from each subject. Different colors indicate the samples obtained with different DNA extraction methods. (PDF) [file pcbi.1006143.s010.pdf]
