# Supplementary material for: Latent environment allocation of microbial community data
Source: PLoS Comput Biol. 2018 Jun 6;14(6):e1006143. doi: 10.1371/journal.pcbi.1006143 (PMC6005635; doi:10.1371/journal.pcbi.1006143)

A

EMPO-Level 3 “Water (saline)” … e.g. salt water from ocean, sea, estuary, mangrove, coral reef (>5 psu)

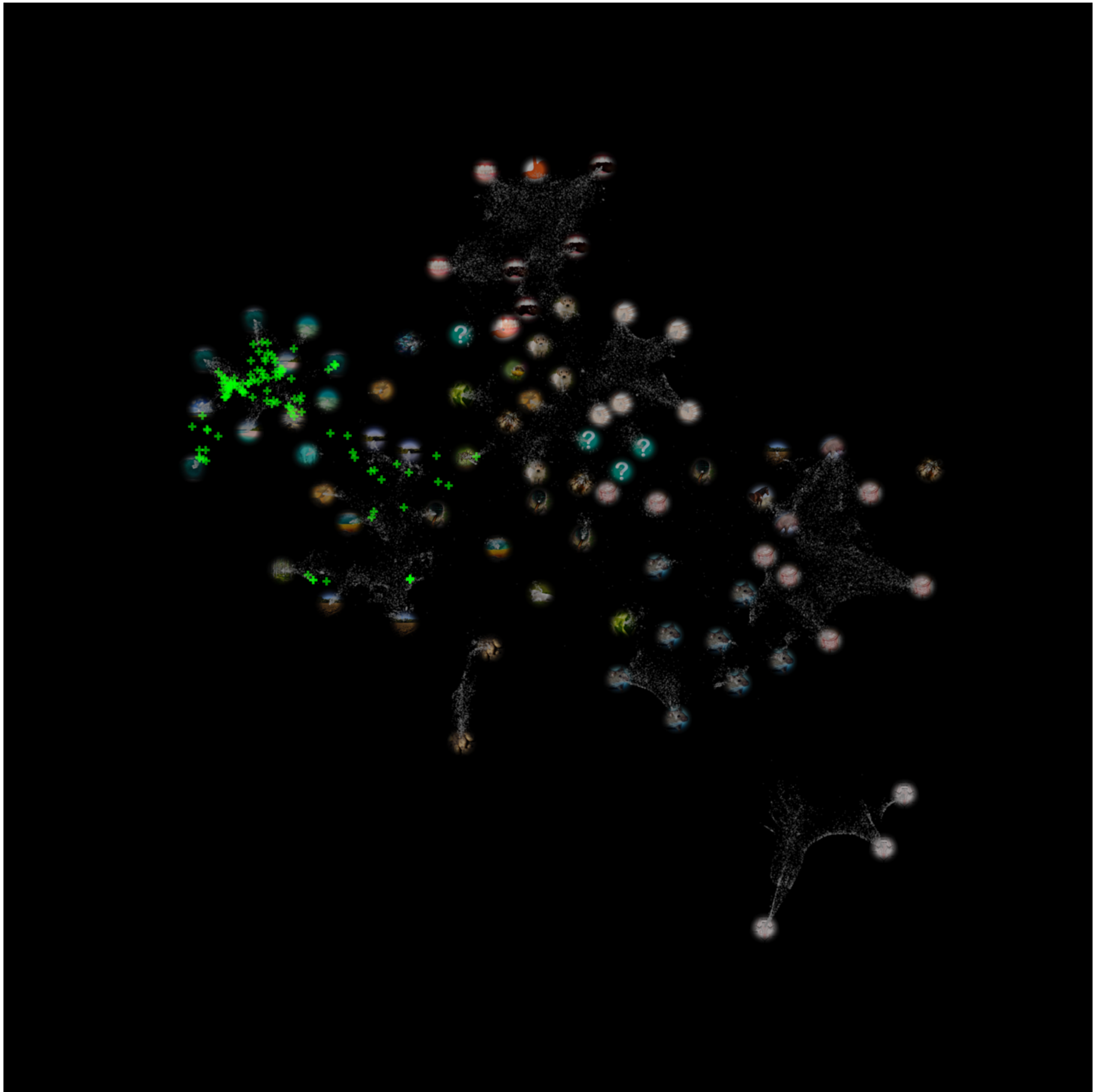

B

EMPO-Level 3 “Water (non-saline)” … e.g. fresh water from lake, pond, river (<5 psu)

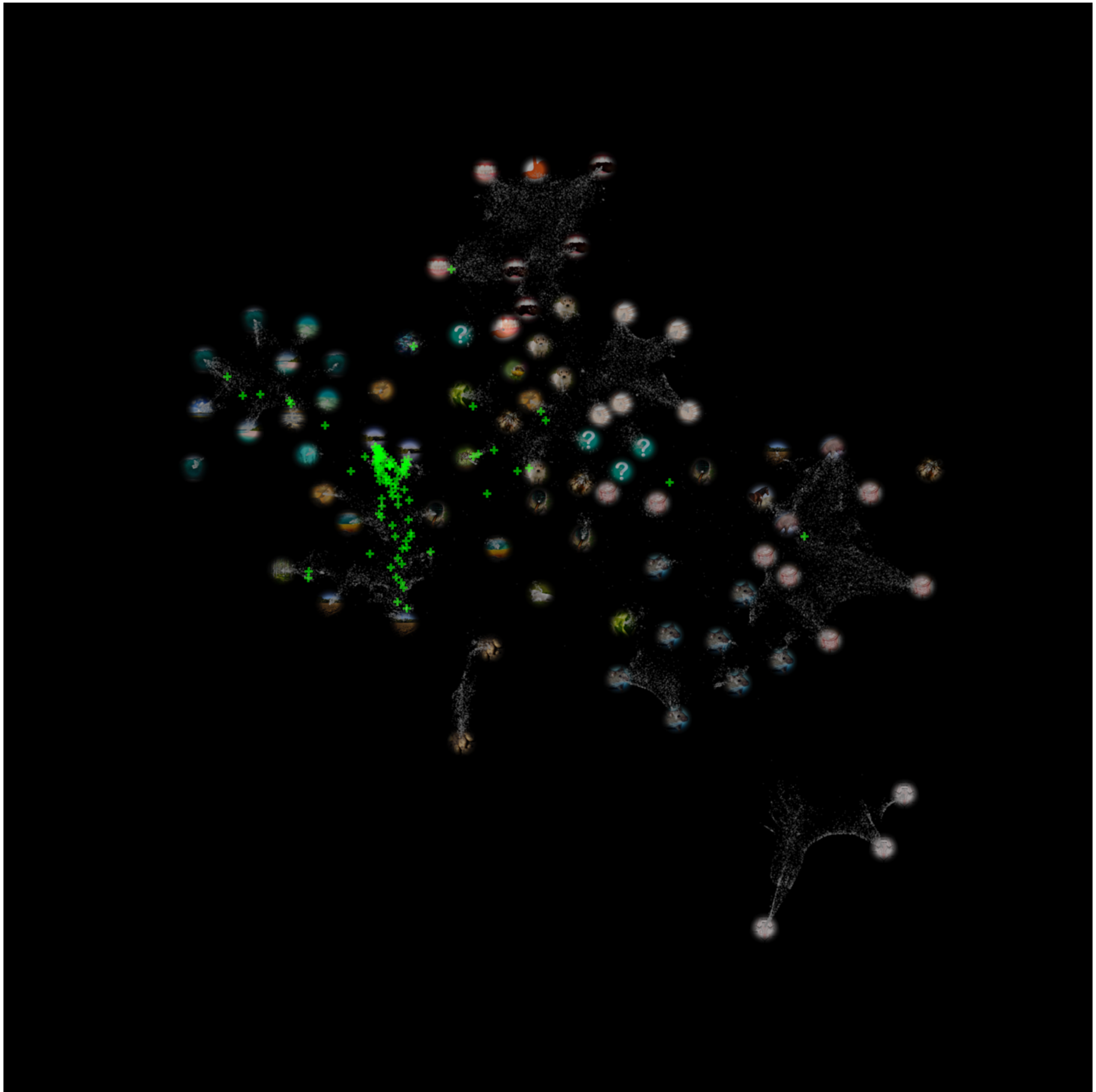

C

EMPO-Level 3 “Sediment (saline)” … e.g. sediment from ocean, sea, estuary, mangrove, beach (>5 psu)

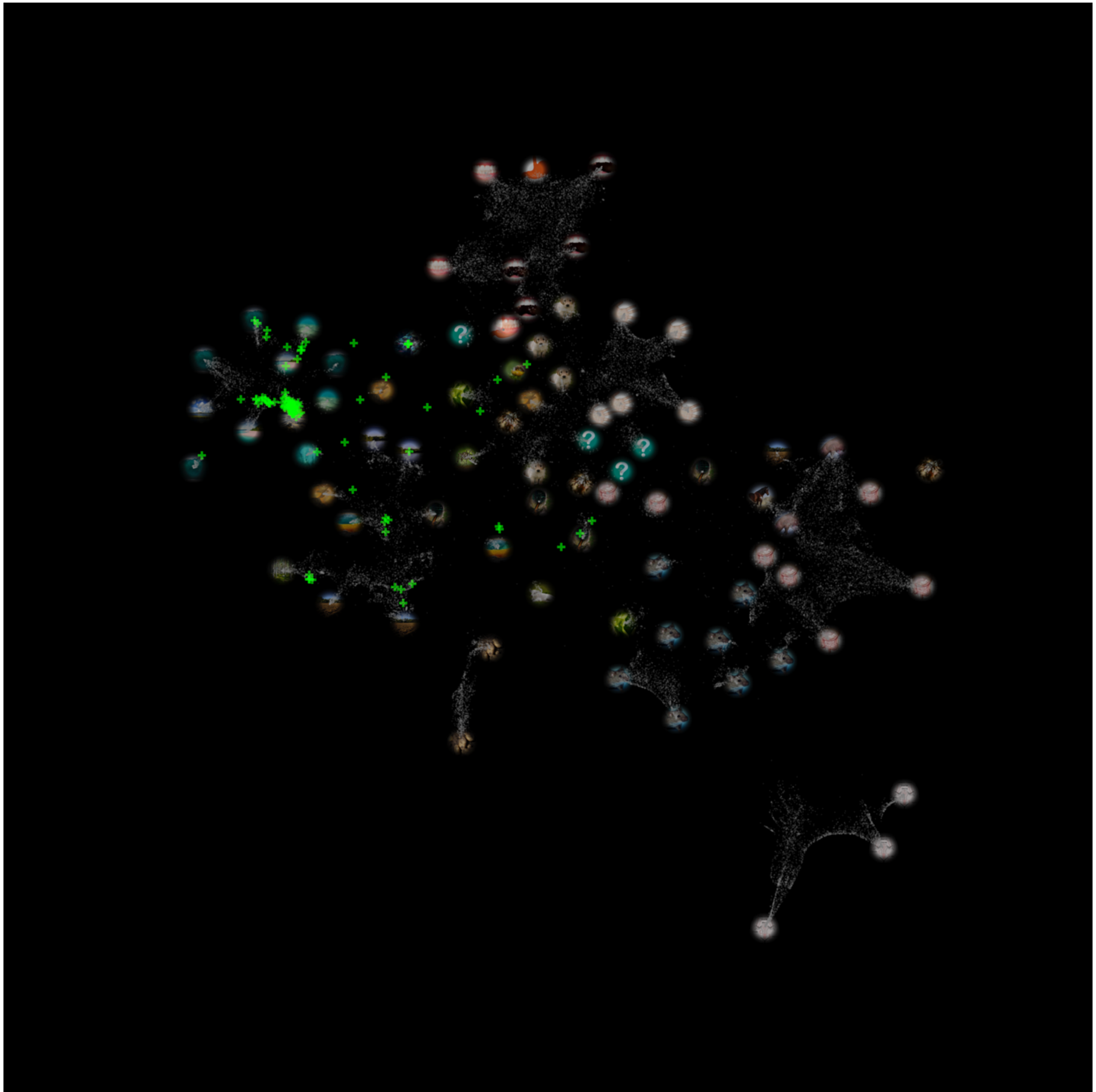

D

EMPO-Level 3 “Sediment (non-saline)” … e.g. sediment from lake, pond, river (<5 psu)

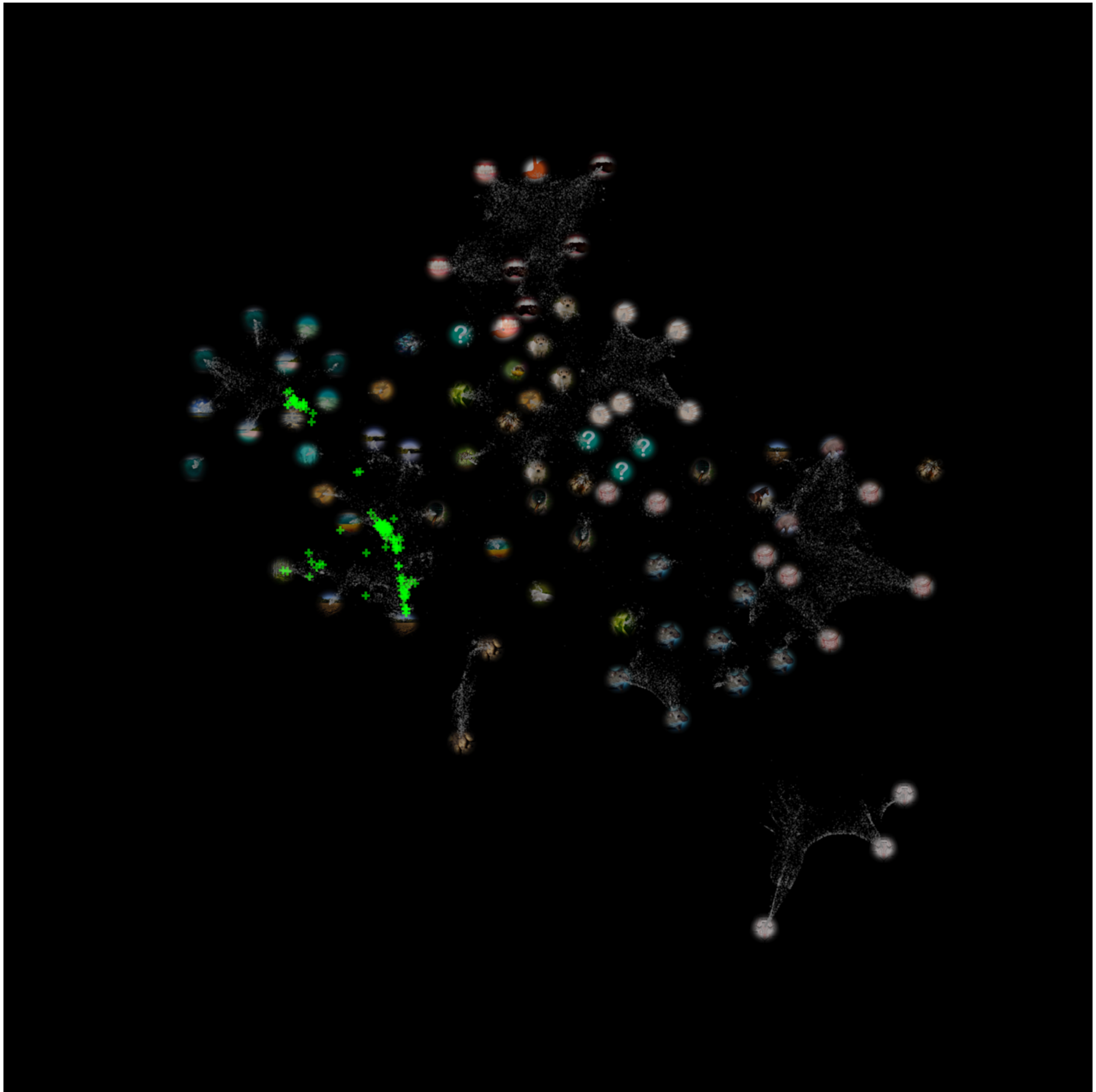

E

EMPO-Level 3 “Hypersaline (saline)” … e.g. water from hypersaline sample or brine (>50 psu)

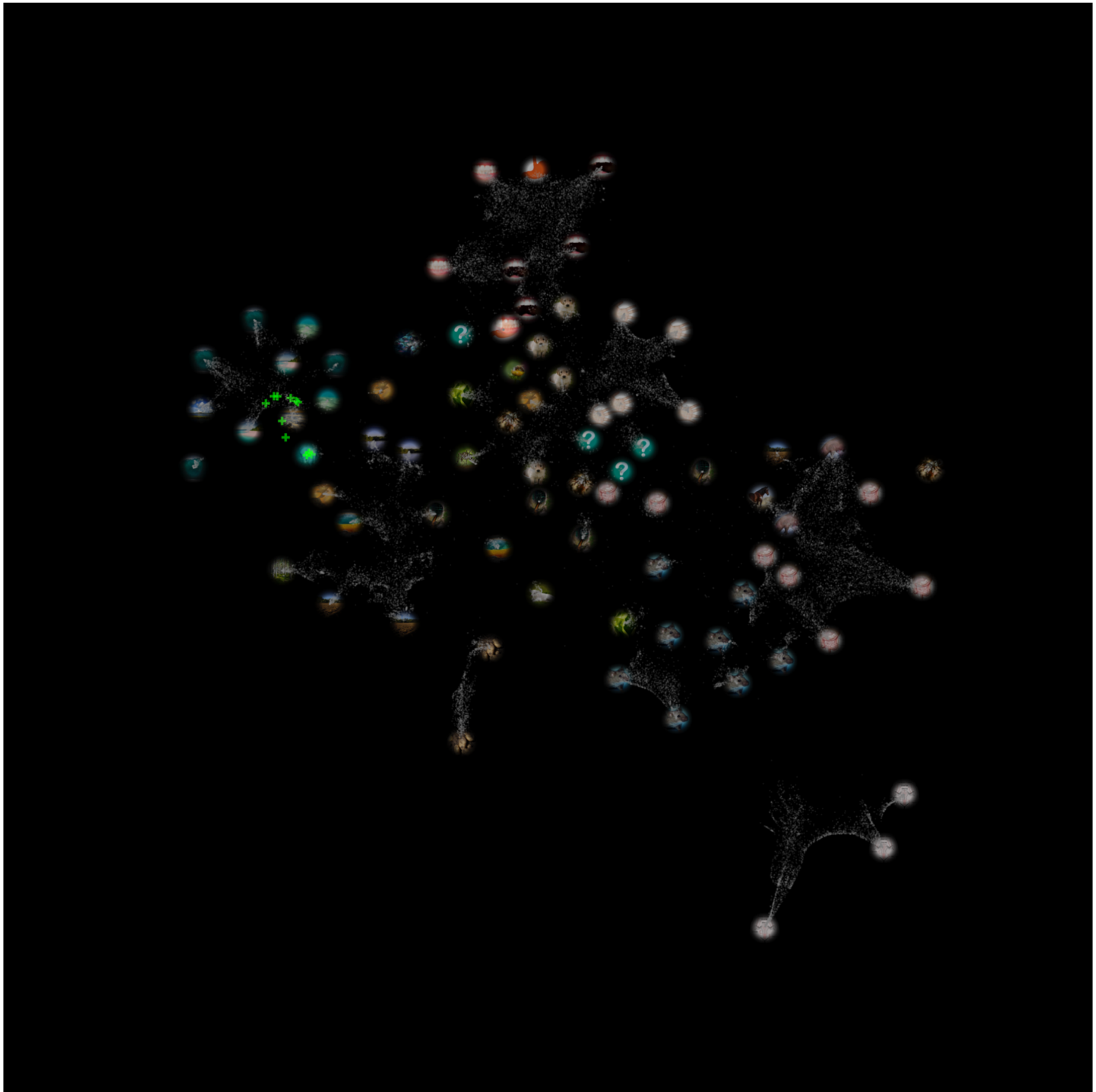

F

EMPO-Level 3 “Soil (non-saline)” … e.g. soil from forest, grassland, tundra, desert.

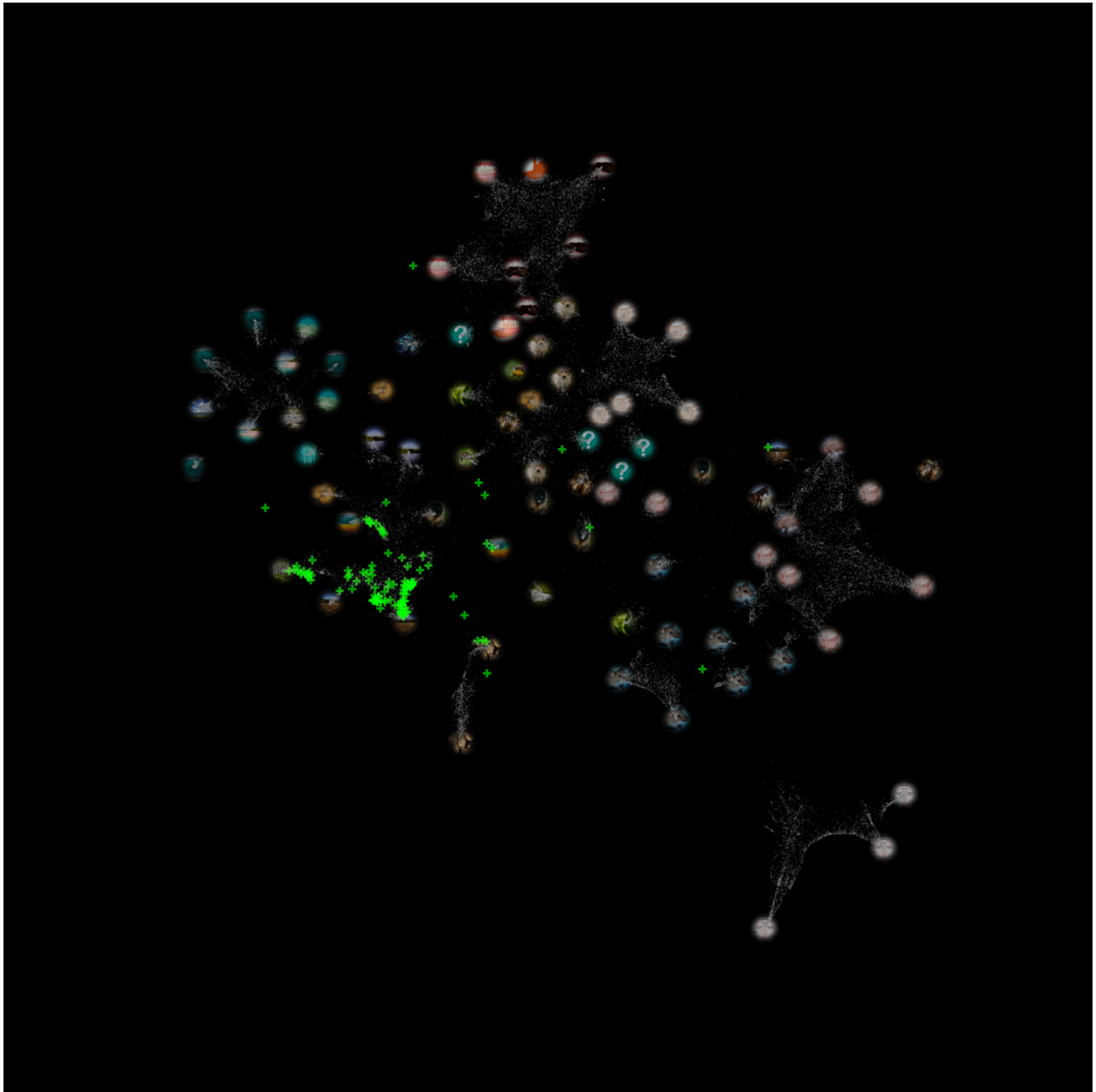

G

EMPO-Level 3 “Surface (saline)” … e.g. biofilm from wet or underwater surface or microbial mat (>5 psu)

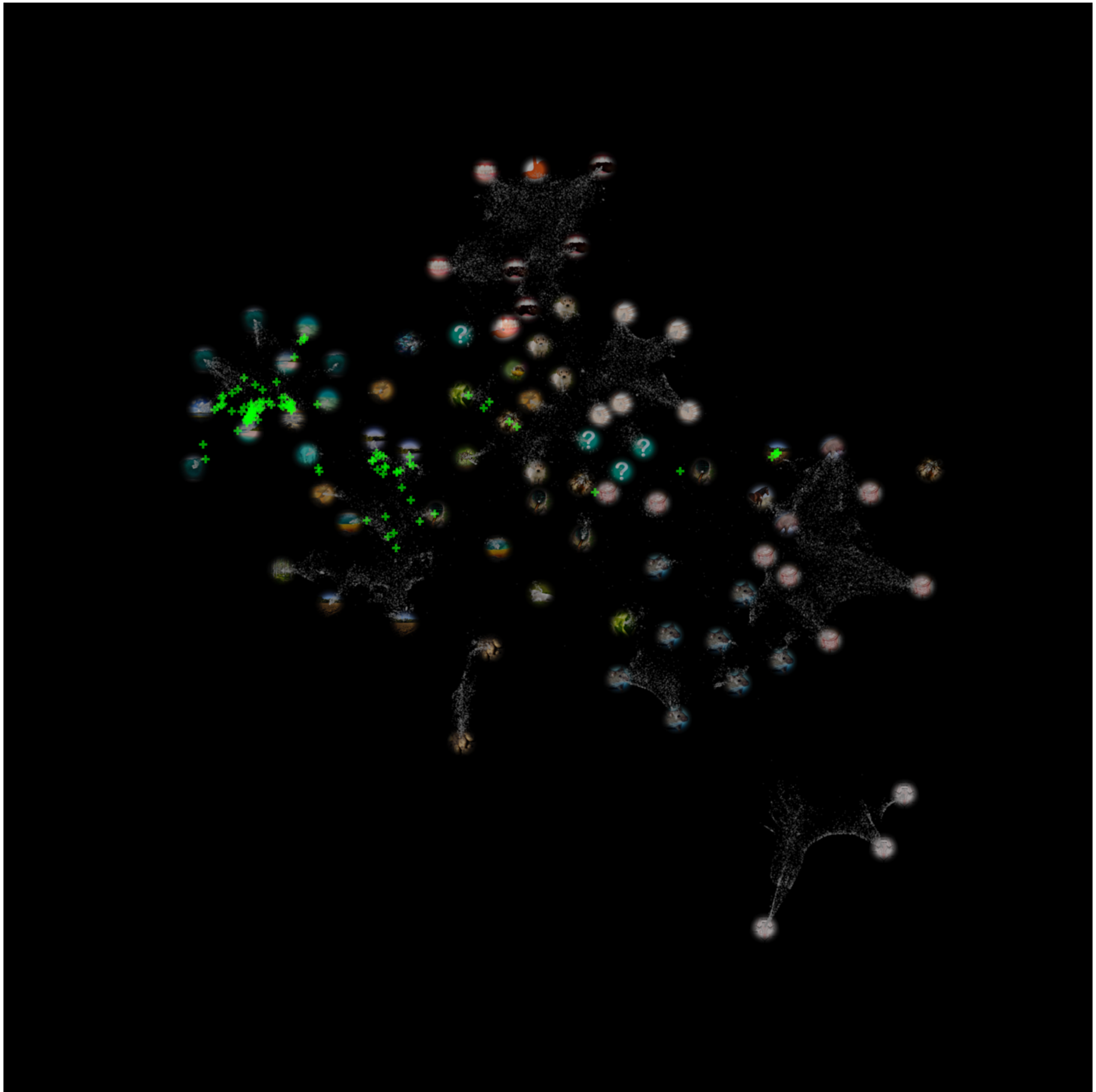

H

EMPO-Level 3 “Surface (non-saline)” … e.g. biofilm from wet (<5 psu) or dry surface, wood, dust, microbial mat

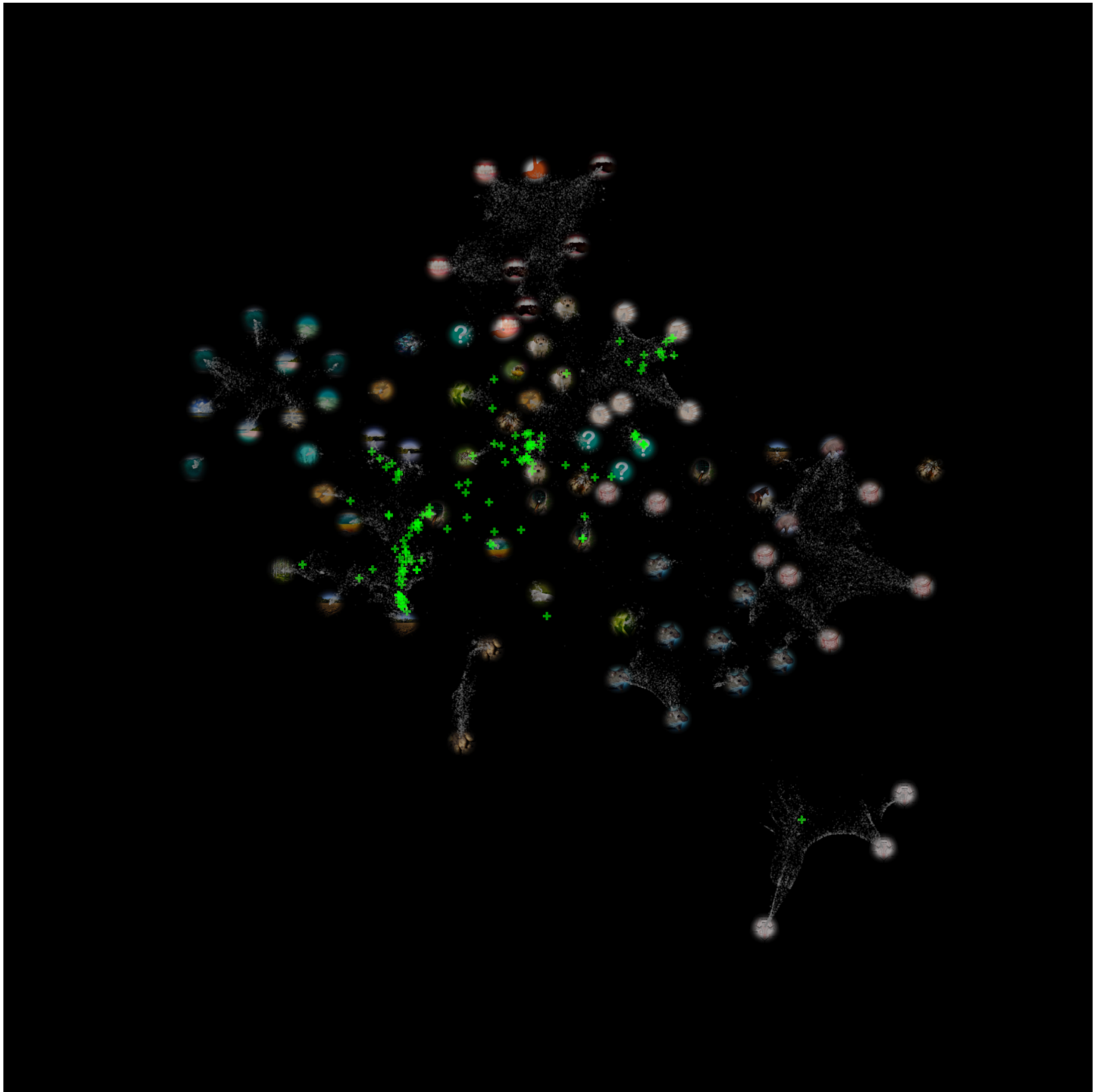

I

EMPO-Level 3 “Aerosol (non-saline)” … e.g. aerosolized dust or liquid

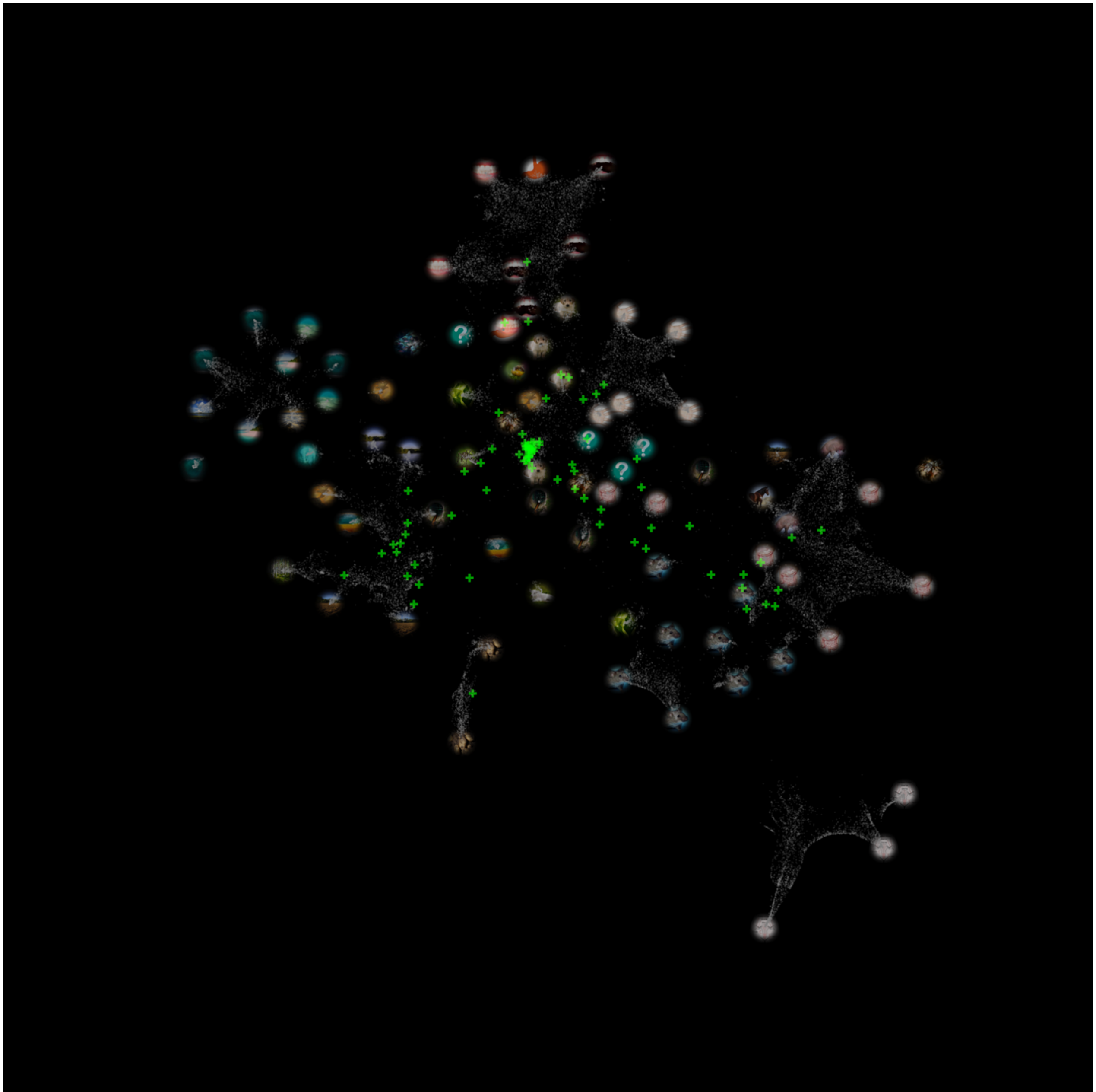

J

EMPO-Level 3 “Plant surface” … e.g. leaf or kelp surface biofilm

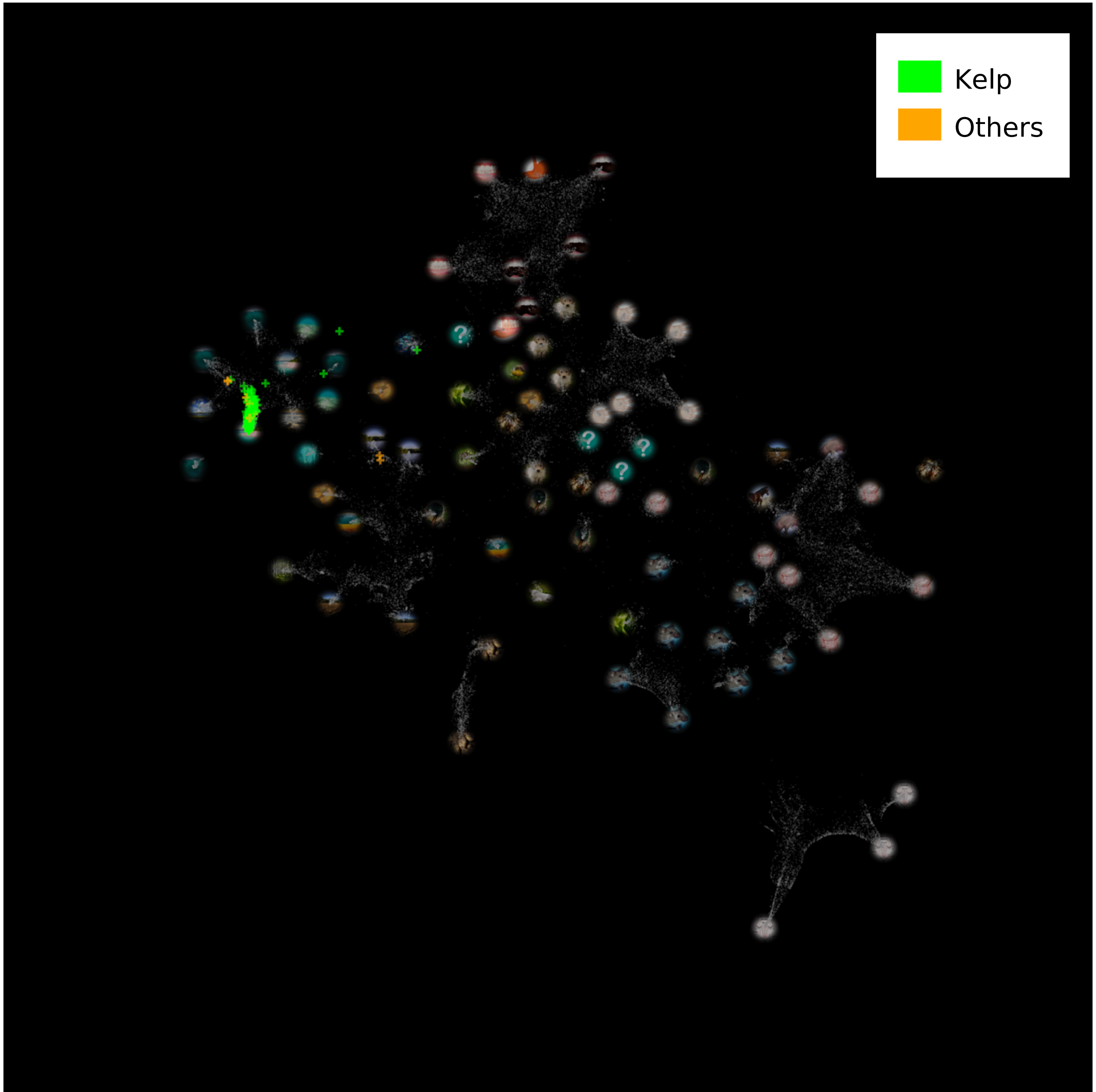

K

EMPO-Level 3 “Plant rhizosphere” … e.g. plant root system, may include some soil

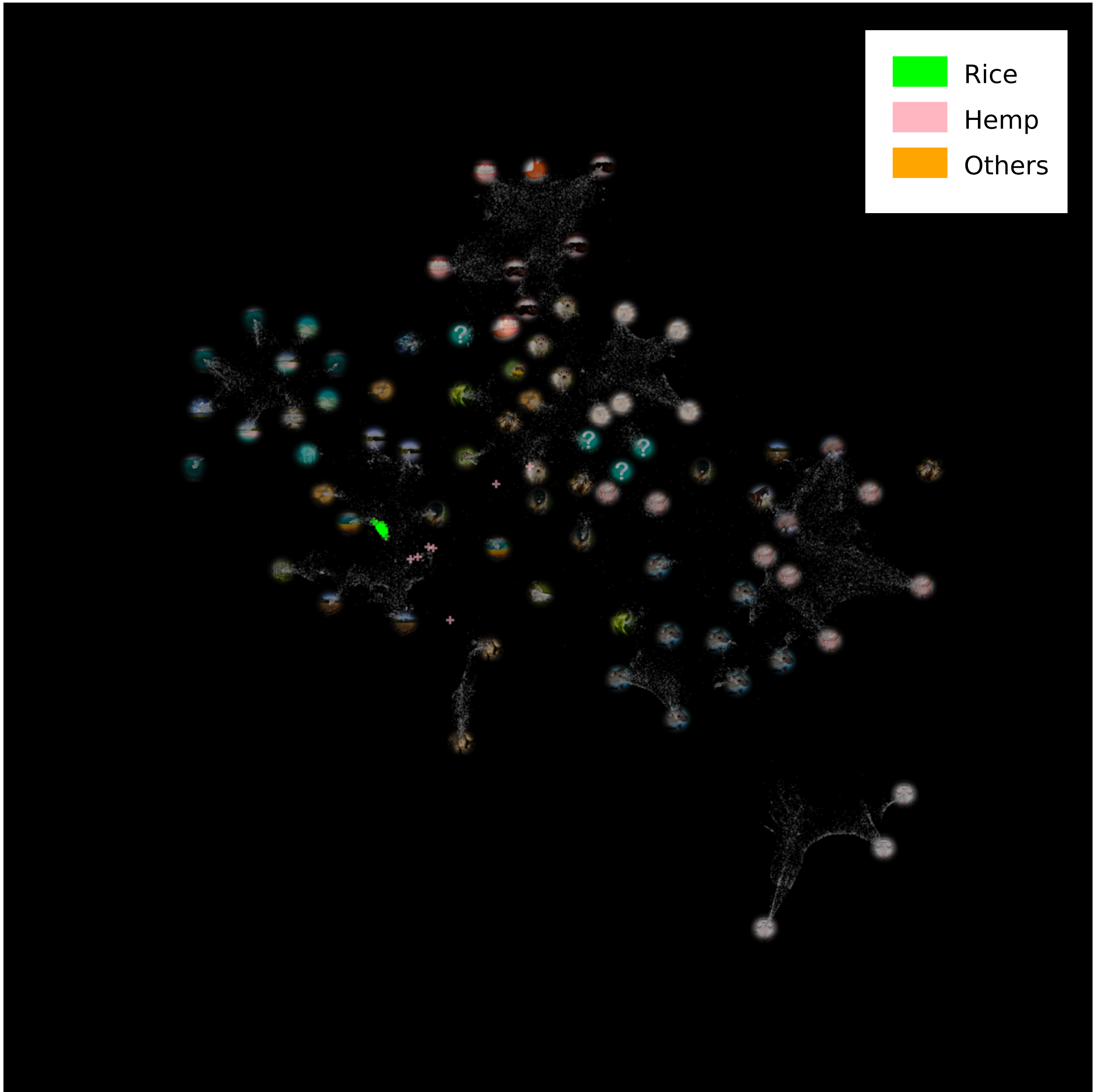

L

EMPO-Level 3 “Plant corpus” … e.g. tissue of leaf, stem, fruit, algae

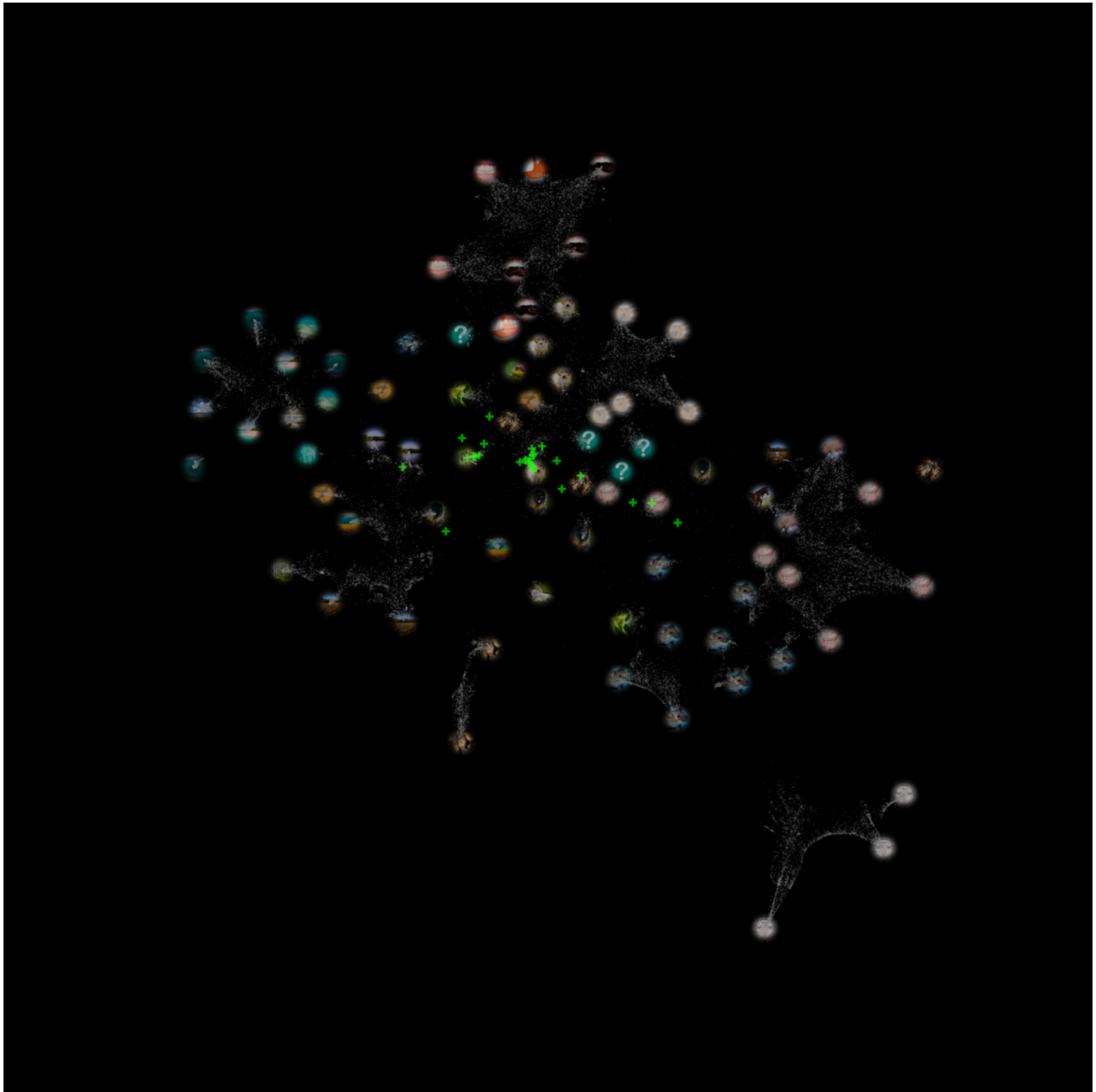

M

EMPO-Level 3 “Animal distal gut” … e.g. feces, stool

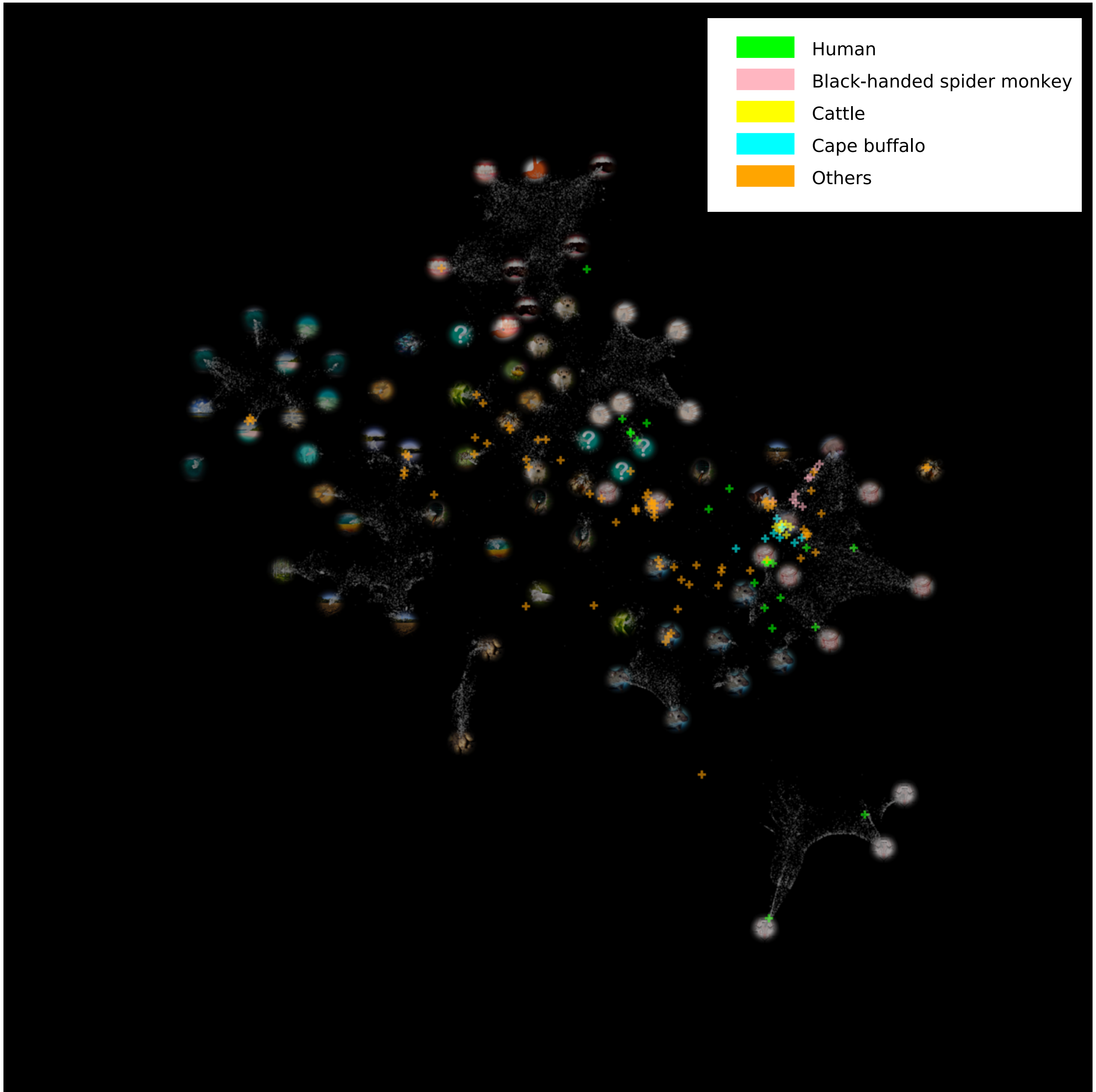

N

EMPO-Level 3 “Animal proximal gut” … e.g. gut intestine, gizzard, crop, lumen, mucosa

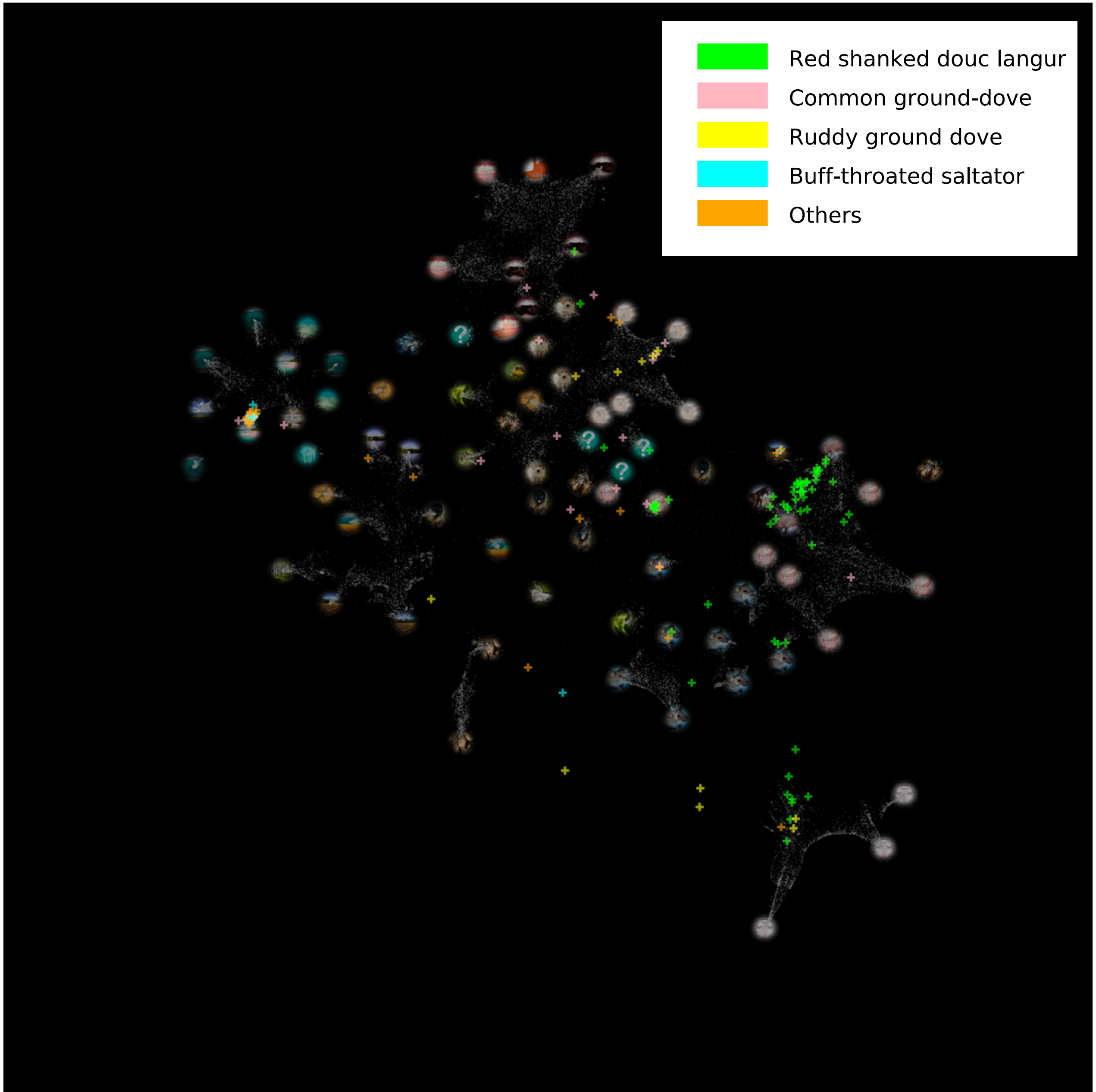

O

EMPO-Level 3 “Animal secretion” … e.g. saliva, breast milk, vaginal secretion

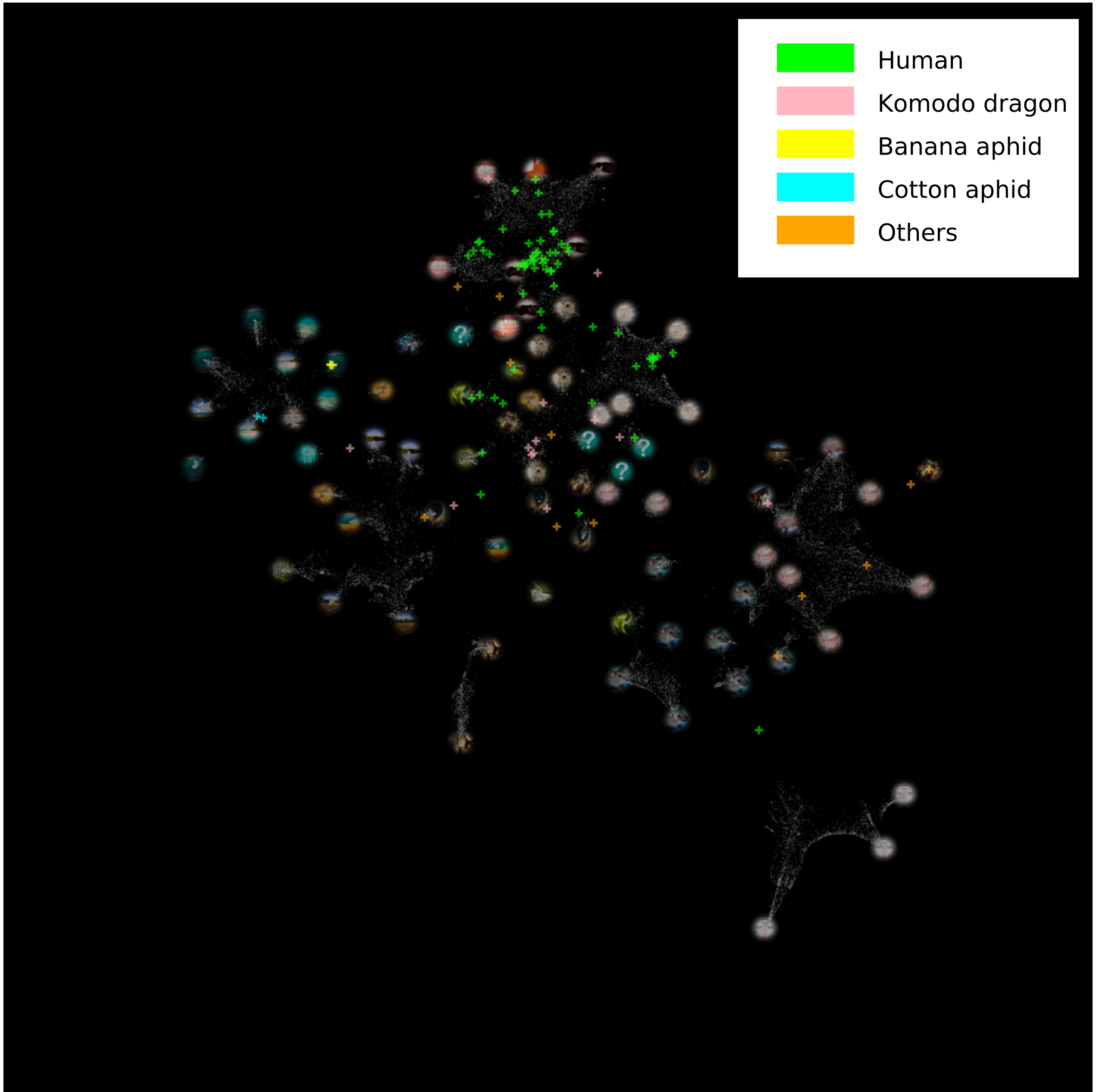

P

EMPO-Level 3 “Animal surface” … e.g. skin, sebum, mucus, slime

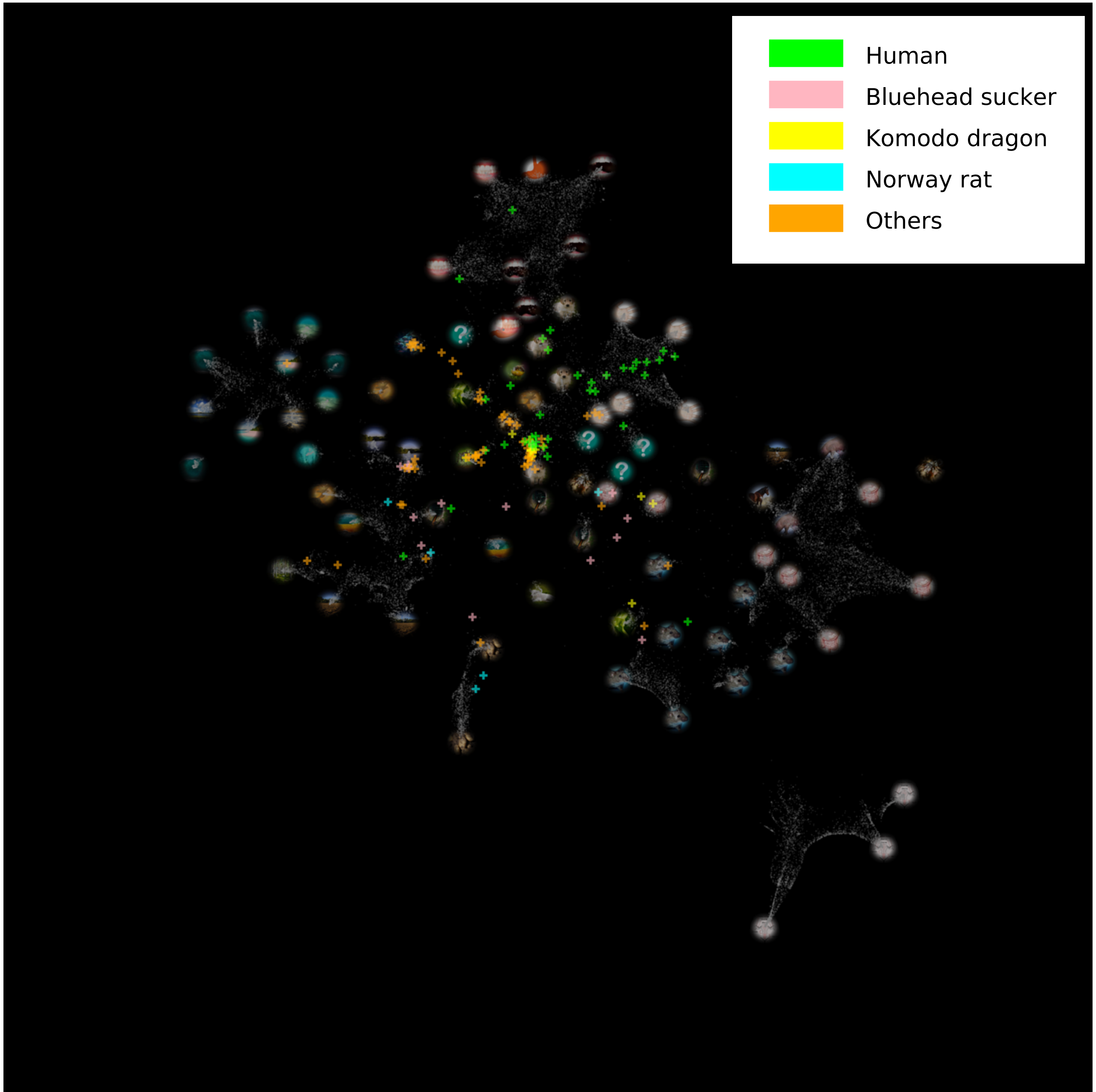

Q

EMPO-Level 3 “Animal corpus” … e.g. tissue of sponge, coral, gill, siphon, carcass, etc. or whole small animal

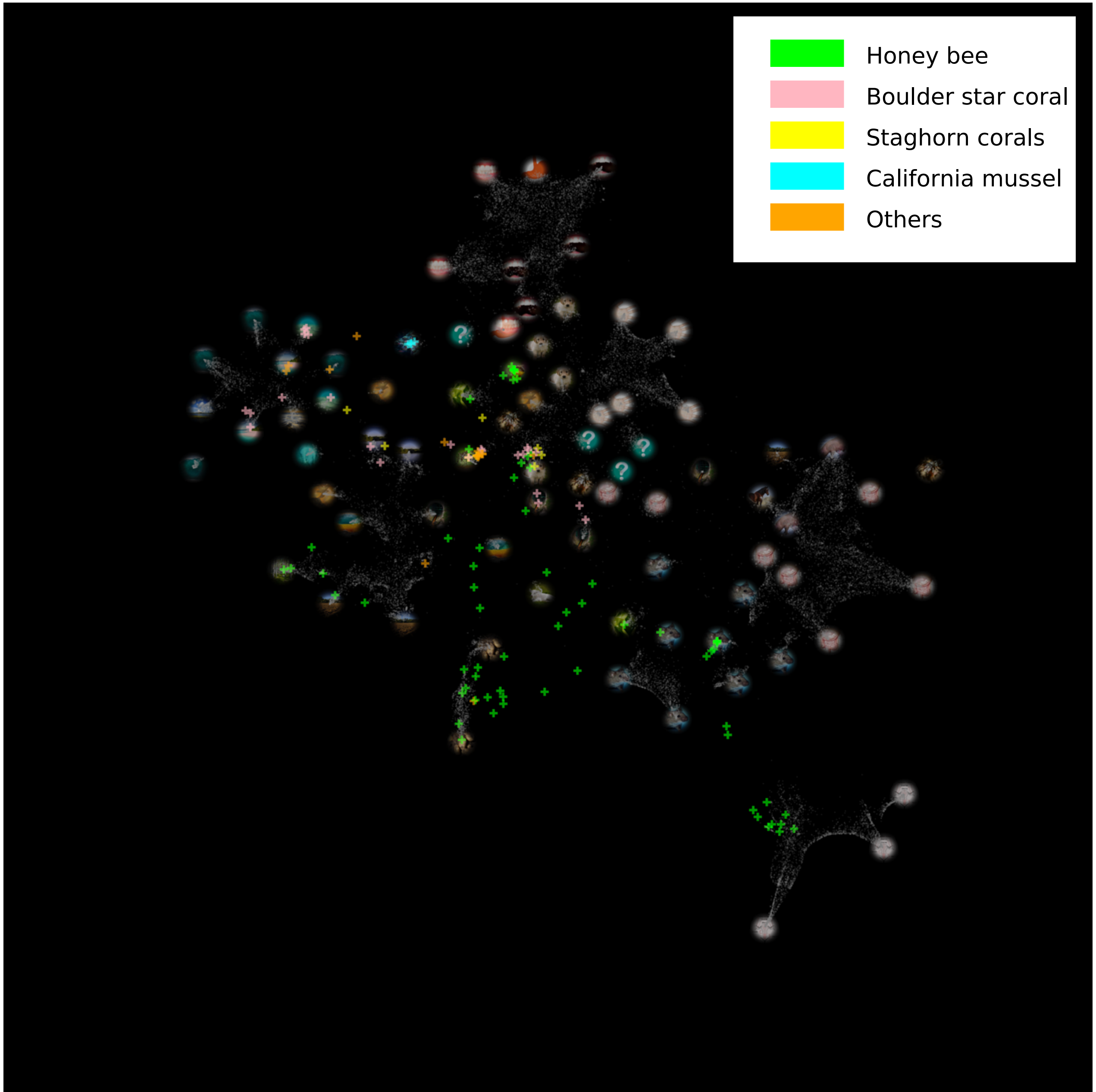

Supplement: S11 Fig — LEA mapping results of 2,000 subset of Earth Microbiome Project (EMP) dataset. Groups of samples were separately displayed according to their EMP Ontology-Level 3 labels. (A-Q) Colored crosses indicate EMP samples. In panels J, K, M, N, O, P, and Q, samples were colored by their host species. (PDF) [file pcbi.1006143.s011.pdf]
